# Exposure to Household Air Pollution From Biomass Cooking and Severe Pneumonia in Infants

**DOI:** 10.1001/jamanetworkopen.2025.38721

**Published:** 2025-10-29

**Authors:** John P. McCracken, Eric D. McCollum, Kyle Steenland, Laura Nicolaou, Miles A. Kirby, Laura M. Grajeda, Ghislaine Rosa, Alexie Mukeshimana, Florein Ndagijimana, Kalpana Balakrishnan, Gurusamy Thangavel, Sarada S. Garg, Sankar Sambandam, Krishnendu Mukhopadhyay, Vigneswari Aravindalochanan, Anaité Díaz-Artiga, Adly Castañaza, Lisa M. Thompson, Marilú Chiang, Stella M. Hartinger, Suzanne M. Simkovich, Lance A. Waller, Howard H. Chang, Shirin Jabbarzadeh, Jiantong Wang, Yunyun Chen, Luke P. Naeher, Ajay Pillarisetti, Michael Johnson, Kendra N. Williams, Lindsay J. Underhill, Aris T. Papageorghiou, Thomas F. Clasen, Jennifer L. Peel, William Checkley

**Affiliations:** 1 Department of Epidemiology and Biostatistics, College of Public Health, University of Georgia, Athens; 2 Center for Health Studies, Universidad del Valle de Guatemala, Guatemala City, Guatemala; 3 Global Program in Pediatric Respiratory Sciences, Eudowood Division of Pediatric Respiratory Sciences, School of Medicine, Johns Hopkins University, Baltimore, Maryland; 4 Center for Global Non-Communicable Disease Research and Training, School of Medicine, Johns Hopkins University, Baltimore, Maryland; 5 Gangarosa Department of Environmental Health, Rollins School of Public Health, Emory University, Atlanta, Georgia; 6 Division of Pulmonary and Critical Care, School of Medicine, Johns Hopkins University, Baltimore, Maryland; 7 Department of Global Health and Population, Harvard T.H. Chan School of Public Health, Boston, Massachusetts; 8 Department of Public Health, Policy and Systems, University of Liverpool, Liverpool, United Kingdom; 9 Eagle Research Center, Kigali, Rwanda; 10 Department of Environmental Health Engineering, Sri Ramachandra Institute for Higher Education and Research, Chennai, India; 11 Department of Family Health Care Nursing, University of California, San Francisco; 12 Biomedical Research Unit, A.B. PRISMA, Lima, Peru; 13 Facultad de Salud Pública y Administración, Universidad Peruana Cayetano Heredia, Lima, Peru; 14 Division of Healthcare Delivery Research, MedStar Health Research Institute, Columbia, Maryland; 15 Division of Pulmonary and Critical Care Medicine, Georgetown University, Washington, DC; 16 Department of Biostatistics and Bioinformatics, Rollins School of Public Health, Emory University, Atlanta, Georgia; 17 Department of Environmental Health Science, College of Public Health, University of Georgia, Athens; 18 Division of Environmental Health Sciences, School of Public Health, University of California, Berkeley; 19 Berkeley Air Monitoring Group, Berkeley, California; 20 Program in Social and Behavioral Interventions, Bloomberg School of Public Health, Johns Hopkins University, Baltimore, Maryland; 21 Cardiovascular Division, Department of Medicine, Washington University in St Louis, St Louis, Missouri; 22 Nuffield Department of Women’s and Reproductive Health, University of Oxford, Oxford, United Kingdom; 23 Department of Environmental and Radiological Health Sciences, Colorado State University, Fort Collins

## Abstract

**Importance:**

Household air pollution from biomass cooking is considered an important risk factor for child pneumonia.

**Objective:**

To evaluate the longitudinal association between exposure to particulate matter with a diameter of less than or equal to 2.5 µm (PM_2.5_) or carbon monoxide (CO) and severe pneumonia in infants.

**Design, Setting, and Participants:**

This cohort study included infants (aged ≤12 months) whose mothers participated in a 4-country randomized clinical trial. Conducted from May 2018 to September 2021, the trial tested whether an 18-month liquefied petroleum gas stove and fuel distribution intervention reduced the incidence of severe pneumonia in offspring during infancy when compared with biomass cooking. The trial was conducted in communities where residents cooked primarily with biomass fuels in Guatemala, India, Peru, and Rwanda. Data analysis was conducted from December 2024 to July 2025.

**Exposures:**

Twenty-four hour personal exposure to PM_2.5_ and CO was measured 3 times during pregnancy and 3 times during infancy.

**Main Outcome and Measures:**

In this exposure-response analysis, severe pneumonia cases were identified using respiratory signs and symptoms with confirmation of consolidation by imaging and hypoxemia by pulse oximetry. The longitudinal association between severe pneumonia in infants and PM_2.5_ or CO exposures by infant-quarters, adjusted for confounders, was modeled.

**Results:**

Overall, 3061 infants (48.2% girls; mean [SD] gestational age at birth, 39.3 [1.7] weeks) contributed 11 996 infant-quarters and 13 910 measurements of personal PM_2.5_ exposures (range, 5.4-1182.0 µg/m^3^). A total of 175 episodes of severe pneumonia were identified in 160 infants. Those with at least 1 episode of severe pneumonia had similar mean (SD) prenatal (101 [100] µg/m^3^ vs 88 [80] µg/m^3^; *P* = .11) and postnatal (70 [78] µg/m^3 ^vs 67 [93] µg/m^3^; *P* = .68) PM_2.5_ exposures when compared with infants without severe pneumonia. There were no associations between prenatal (adjusted risk ratio [RR], 1.03; 95% CI, 0.94-1.13) or postnatal (adjusted RR, 0.97; 95% CI, 0.87-1.09) PM_2.5_ exposures and severe pneumonia or between CO exposures and severe pneumonia.

**Conclusions and Relevance:**

In this cohort study with exposure-response analysis, there was no evidence of an association between longitudinal PM_2.5_ or CO exposures and severe pneumonia in infants. Taken with the intention-to-treat analysis from the randomized clinical trial, these results challenge prior research suggesting that PM_2.5_ or CO exposures from biomass cooking are an important risk factor for severe pneumonia in infants.

## Key Points

**Question:**

Are personal exposures to fine particulate matter and carbon monoxide during pregnancy and infancy associated with increased risk of severe pneumonia in infants?

**Findings:**

This cohort study included 3061 infants, with 11 996 infant-quarters of active health care facility surveillance and 13 910 personal exposure assessments in 4 low- and middle-income countries. No associations between prenatal or postnatal fine particulate matter exposures and severe pneumonia or between personal carbon monoxide exposures and severe pneumonia were found.

**Meaning:**

The findings of this study suggest that household-level mitigation strategies to reduce fine particulate matter and carbon monoxide exposures with cleaner cooking may not have as meaningful an impact on severe pneumonia in infants as previously estimated.

## Teaser

This cohort study evaluates whether prenatal and postnatal exposures to fine particulate matter and carbon monoxide due to biomass cooking are associated with the risk of severe pneumonia in infants in low- and middle-income settings.

## Introduction

Lower respiratory infections (LRIs), including pneumonia, remain the largest infectious causes of death in children, with most deaths occurring in low- and middle-income countries (LMICs) and among infants.^1^ In 2021, LRIs caused 38 million episodes and 502 000 deaths in children younger than 5 years worldwide.^2^ While the incidence and mortality from pneumonia has declined substantially between 1990 and 2021, it is still responsible for 14% of global deaths in children younger than 5 years.^2,3^ Infants are an especially vulnerable population, with nearly 60% of all child pneumonia deaths occurring during infancy.^4^ While vaccines are recognized as the cornerstone of pneumonia prevention strategies, there remain major gaps in our understanding of the importance of other risk factors, including air pollution.^5^

Air pollution has been identified as an important risk factor for pneumonia and other LRIs in children. This is supported by several early observational studies of the association between household air pollution (HAP) and pneumonia.^6,7^ A recent meta-analysis^8^ found that HAP from solid fuel use was associated with a 39% higher risk of having an acute respiratory infection in children. Evidence from randomized clinical trials (RCTs), however, does not support the association. Intention-to-treat analysis from several moderate-to-large sized RCTs in Guatemala, Nepal, Malawi, and Ghana that tested improved biomass burning stoves or cleaner fuels, such as liquefied petroleum gas (LPG), did not find an effect on child pneumonia or LRIs.^9,10,11,12^ Interventions used in these trials did not achieve the exposure contrasts between study groups believed to be necessary to have a meaningful impact on child pneumonia outcomes. This was the main motivation for conducting the Household Air Pollution Intervention Network (HAPIN) trial, an 18-month RCT conducted among pregnant women in 4 low- and middle-income countries to test whether an intervention consisting of an LPG stove, continuous fuel distribution, and behavioral messaging to encourage LPG use improved multiple health outcomes—including a reduction in the incidence of severe pneumonia in infants—when compared to cooking with biomass fuels.^13^

The HAPIN intervention achieved an approximately 60% reduction in personal exposures to fine particulate matter with a diameter of less than or equal to 2.5 µm (PM_2.5_) in both the prenatal and postnatal periods,^14,15^ but consistent with other trials, the intervention did not reduce the incidence of severe pneumonia in infants.^16^ While intention-to-treat analyses are the gold standard to make recommendations for policy or practice decisions about an intervention, the global burden of disease estimates of air pollution risk are based predominantly on observational studies.^8,17^ Moreover, large reductions in the global burden of disease from air pollution have been achieved by policies informed chiefly from exposure-response analyses. We conducted an exposure-response analysis of the association between PM_2.5_ or carbon monoxide (CO) exposures and severe pneumonia in infants using data from the HAPIN trial.

## Methods

### Study Design and Setting

For this cohort study, we conducted an exposure-response analysis using data from a 4-country RCT (May 2018 to September 2021) involving 3200 pregnant women, designed to assess whether an LPG stove intervention reduced severe pneumonia in infants compared with biomass cooking. Details of the trial were published elsewhere.^13^ Participants were pregnant women (aged 18-34 years, with 9-19 weeks of gestation confirmed by ultrasonography) from Guatemala, India, Peru, and Rwanda, all using biomass fuels at baseline.

Participants were randomized equally to receive either an LPG stove with continuous fuel supply and behavioral messaging or to continue usual biomass cooking. Women and their offspring were followed up until the infant’s first birthday. Active surveillance for severe pneumonia occurred at sentinel health care facilities, and personal exposures to PM_2.5_ and CO were measured 3 times during pregnancy and 3 times in infancy. The study protocol and analyses presented in this article were reviewed and approved by the institutional review boards and ethics committees at Emory University, Johns Hopkins University, Sri Ramachandra Institute of Higher Education and Research, the Indian Council of Medical Research–Health Ministry Screening Committee, Universidad del Valle de Guatemala, the Guatemalan Ministry of Health National Ethics Committee, A.B. PRISMA, the London School of Hygiene and Tropical Medicine, the Rwandan National Ethics Committee, and Washington University in St Louis. All participants provided written informed consent. This report adheres to the Strengthening the Reporting of Observational Studies in Epidemiology (STROBE) reporting guideline for cohort studies.

### Personal Exposure Assessment

We assessed 24-hour personal exposures to PM_2.5_ and CO during pregnancy at baseline [BL], and at 24 to 28 weeks (P1) and 32 to 36 weeks (P2) of gestation. Infant exposures were measured at 3 (B1), 6 (B2), and 12 (B4) months.^18^ We used Enhanced Children’s MicroPEM devices for PM_2.5_ and Lascar EL-USB-CO monitors for CO. Pregnant women wore devices in locally adapted vests or aprons. As infants could not carry monitors, we used indirect methods^19^: mothers wore monitors, and area concentrations were measured in kitchens, patios, and infant sleeping areas. Infants wore proximity beacons to track their time spent near the mother or in specific microenvironments. Personal exposure estimates for infants were derived from these data.^18,19,20^ We report additional details for personal exposure assessment in the eMethods in Supplement 1.

### Reducing Missing Exposure Data

We addressed missing personal exposure data (either PM_2.5_ or CO) via replacement or imputation. For missing postnatal data, we substituted the mother’s concurrent exposure data. For missing prenatal data, intervention group participants with missing baseline values had them imputed as D_country_ + (P1 + P2)/2, where D_country_ denotes the mean of BL − (P1 + P2)/2, calculated for each country using complete sets (BL, P1, P2). Participant-specific prenatal exposures were calculated as the mean of BL (observed or imputed) and the mean of observed postbaseline values (P1 and/or P2). For controls, we used the mean of all available prenatal exposure values. Postnatal exposure was divided into 4 quarters. For the first quarter, we used the mean of P2 and B1 (if both were missing, we used B2; if B2 was missing, we used B4); for the second and third quarters, we used the mean of P2, B1, and B2; and for the fourth quarter, we used the mean of P2, B1, B2, and B4.

### Outcome Assessment

The primary outcome was severe pneumonia during infancy,^21,22^ ascertained using active surveillance at sentinel health facilities.^23^ Severe pneumonia was defined by health care–seeking for cough and/or difficulty breathing plus a general or neonatal danger sign (eMethods in Supplement 1) and confirmed lung consolidation on ultrasonography or chest radiography (if available); presence of hypoxemia (oxygen saturation measured by pulse oximetry [SpO_2_] ≤92% at <2500 m elevation or ≤86% at ≥2500 m elevation)^24^; or confirmed pneumonia-related death via verbal autopsy. We used the World Health Organization (WHO) Integrated Management of Childhood Illness (IMCI) definition for severe pneumonia^25^ as a secondary outcome.

We conducted a geographic and resource availability survey at all health care facilities in our study areas to identify sentinel facilities where infants with severe pneumonia would receive care.^23^ We then posted study personnel at these facilities to surveil all infants admitted with severe pneumonia (eMethods in Supplement 1). All suspected pneumonia cases were imaged using lung ultrasonography^26^; radiographs were included when ultrasonography was not available. Images were interpreted by blinded adjudication panels.^27^ Most signs and symptoms were collected directly by study staff; medical records were secondary sources. Verbal autopsies were reviewed by trained adjudication panels for infant deaths. All infants were included, regardless of birth defects, which were rare at 1.6%.^28^

### Statistical Analysis

The statistical analysis plan was finalized before unblinding and is publicly available (NCT02944682). Analyses were independently replicated. We used log-binomial regression to estimate the risk ratio (RR) of severe pneumonia per infant-quarter associated with PM_2.5_ or CO exposures. We used infant-quarters (3-month intervals) as the unit of person-time to capture recurrent pneumonia episodes and align with exposure measurements. The occurrence of severe pneumonia in an infant-quarter was the primary end point. Models adjusted for quarter of follow-up, household crowding (quartiles), infant sex, birth weight, winter season indicator, COVID-19 pandemic period indicator, socioeconomic wealth index (quartiles),^29^ pneumococcal conjugate vaccine (PCV) status, and country indicators (see directed acyclic graph in eMethods in Supplement 1). Exposure variables (PM_2.5_ and CO), season, COVID-19 period, and PCV status were time-dependent. We describe calculation of the socioeconomic wealth index in the eMethods in Supplement 1. We used generalized estimating equations with a compound symmetry structure and robust variance.^30^ Prenatal and postnatal exposures to PM_2.5_ or CO were modeled both continuously (scaled to IQR) and in quartiles. We tested for effect modification by sex, season, socioeconomic wealth index greater than or equal to the median value vs less than the median, and PCV status by including interaction terms. We used 1000 bootstrap replications to estimate 95% CIs for subgroup analyses. Bootstrap techniques may provide more accurate and robust estimates of SEs and confidence intervals, especially in small samples or when the working correlation structure is not known. We did not analyze interactions by country because there were too few severe pneumonia episodes. *P *values and 95% CIs are presented throughout the Results section. We did not prespecify a level of statistical significance.

We conducted a time-to-event sensitivity analysis using Andersen-Gill models^31^ to account for recurrent pneumonia episodes. Weekly intervals were used to model hazard ratios for associations with PM_2.5_, adjusting for the same covariates as in the primary analysis. We applied robust variance estimation to account for within-infant correlation.^32^

Analyses were conducted using R version 4.2.2 (R Project for Statistical Analysis)^33^ and replicated in SAS version 9.4 (SAS Institute). We provide the statistical code in R used to generate our results in the eAppendix in Supplement 1.

## Results

### Participant Characteristics

Of 3195 eligible pregnant women, 3061 (95.8%) had live births (48.2% girls; mean [SD] gestational age at birth, 39.3 [1.7] weeks). Follow-up was near complete during infancy: 3061 (100% of live births) contributed to follow-up in the first quarter, 2993 (97.7%) in the second, 2978 (97.2%) in the third, and 2964 (96.8%) in the fourth. These 3061 infants contributed 11 996 infant-quarters of a possible 12 244 (98.0%). Incomplete follow-up occurred because 62 infants died, 27 moved out of the study area, and 20 withdrew from the study.

### Personal Exposure Assessments

The 3061 infants had 13 910 personal PM_2.5_ measurements of a possible 18 366 assessments (75.7%). While there were 3892 missing PM_2.5_ infant exposure measurements of 7110 attempted assessments (54.7%) during the postnatal period, this number was reduced to 873 (12.3%) when the missing values were replaced with the mother’s exposure obtained in the same visit. The Spearman correlation between child and mother personal PM_2.5_ exposures obtained on the same day was 0.89 (95% CI, 0.88-0.90). We also observed moderate correlations ranging from 0.44 to 0.52 between prenatal and postnatal exposures to PM_2.5_ (eResults and eFigure 1 in Supplement 1).

A total of 3025 infants (98.8%) had prenatal PM_2.5_ exposure measurements, with a mean (SD) of 88.6 (81.1) µg/m^3^ and ranging between 10.7 µg/m^3^ and 1090.0 µg/m^3^; 2879 infants (94.0%) had postnatal PM_2.5_ exposure estimates, with a mean (SD) of 67.5 (92.1) µg/m^3^ and ranging between 5.4 µg/m^3^ and 1182.0 µg/m^3^. A total of 3037 infants (99.2%) had prenatal CO exposure measurements with a mean (SD) of 1.9 (2.9) parts per million (ppm) and ranging from 0 ppm to 46.4 ppm; 2822 infants (92.2%) had postnatal CO exposure estimates, with a mean (SD) of 1.7 (3.5) ppm and ranging from 0 ppm to 70 ppm. We summarized baseline characteristics by quartiles of mean prenatal and postnatal PM_2.5_ exposures in Table 1.

**Table 1.** Baseline Characteristics by Prenatal PM_2.5_ and Postnatal PM_2.5_ Exposures, Stratified by Quarters

Exposure	Sample size, No.	Mean (SD)				No. (%)			People sleeping in the house, median (IQR), No.	Mean (SD)	
		Maternal age, y	Maternal height, cm	GA at randomization, wk		Girls	Boys			Food insecurity score	Socioeconomic wealth index
**Prenatal PM_2.5_, µg/m^3^**											
≥10.7 to ≤39.6	756	25.4 (4.4)	152.9 (5.9)		15.5 (3.3)	365 (48.3)	391 (51.7)	4 (3-5)		1.1 (1.8)	0.43 (0.18)
>39.6 to ≤65.9	758	25.4 (4.3)	152.3 (6.2)		15.6 (3.0)	368 (48.6)	390 (51.4)	4 (3-5)		1.4 (2.1)	0.38 (0.22)
>65.9 to ≤109.0	738	25.3 (4.6)	152.3 (6.1)		15.4 (3.1)	352 (47.7)	386 (52.3)	4 (3-5)		1.5 (2.2)	0.34 (0.22)
>109.0 to 1090.0	758	25.3 (4.5)	151.3 (6.4)		15.1 (3.1)	367 (48.4)	391 (51.6)	4 (3-5)		1.5 (2.2)	0.34 (0.22)
**Postnatal PM_2.5_ for quarter 1, µg/m^3^**											
>9.8 to 21.4	823	24.9 (4.3)	151.9 (5.2)		15.4 (3.3)	395 (48.0)	428 (52.0)	4 (3-5)		1.1 (1.8)	0.43 (0.17)
>21.4 to ≤37.8	748	25.5 (4.4)	152.6 (6.4)		15.5 (3.1)	370 (49.5)	378 (50.5)	4 (3-5)		1.3 (2.1)	0.37 (0.22)
>37.8 to ≤74.5	698	25.7 (4.5)	152.9 (6.5)		15.5 (3.0)	329 (47.1)	369 (52.9)	4 (3-5)		1.6 (2.2)	0.33 (0.22)
>74.5 to ≤1208.4	734	25.4 (4.6)	151.3 (6.4)		15.1 (3.0)	351 (47.8)	383 (52.2)	4 (3-5)		1.6 (2.2)	0.34 (0.23)
**Postnatal PM_2.5_ for quarter 2, µg/m^3^**											
>9.8 to ≤21.4	723	25.0 (4.3)	151.8 (5.1)		15.4 (3.2)	343 (47.4)	380 (52.6)	4 (3-5)		1.1 (1.8)	0.42 (0.16)
>21.4 to ≤37.8	726	25.4 (4.4)	152.5 (6.5)		15.5 (3.2)	363 (50.0)	363 (50.0)	4 (3-5)		1.2 (2.1)	0.38 (0.21)
>37.8 to ≤74.5	734	25.6 (4.5)	153.3 (6.5)		15.5 (3.0)	353 (48.1)	381 (51.9)	4 (3-5)		1.6 (2.3)	0.33 (0.23)
>74.5 to ≤1208.4	716	25.5 (4.6)	151.1 (6.1)		15.1 (3.0)	341 (47.6)	375 (52.4)	4 (3-5)		1.6 (2.2)	0.35 (0.23)
**Postnatal PM_2.5_ for quarter 3, µg/m^3^**											
>9.8 to ≤21.4	719	25.0 (4.2)	151.9 (5.1)		15.4 (3.2)	342 (47.6)	377 (52.4)	4 (3-5)		1.1 (1.8)	0.43 (0.16)
>21.4 to ≤37.8	725	25.4 (4.4)	152.5 (6.5)		15.5 (3.2)	363 (50.1)	362 (49.9)	4 (3-5)		1.2 (2.1)	0.38 (0.21)
>37.8 to ≤74.5	733	25.6 (4.5)	153.3 (6.5)		15.5 (3.0)	352 (48.0)	381 (52.0)	4 (3-5)		1.6 (2.3)	0.33 (0.23)
>74.5 to ≤1208.4	714	25.5 (4.6)	151.1 (6.1)		15.1 (3.0)	340 (47.6)	374 (52.4)	4 (3-5)		1.6 (2.2)	0.35 (0.23)
**Postnatal PM_2.5_ for quarter 4, µg/m^3^**											
>9.8 to ≤21.4	669	25.3 (4.4)	152.1 (5.0)		15.4 (3.3)	325 (48.6)	344 (51.4)	4 (3-6)		1.2 (1.8)	0.41 (0.15)
>21.4 to ≤37.8	736	25.3 (4.3)	152.4 (6.5)		15.6 (3.2)	370 (50.3)	366 (49.7)	4 (3-5)		1.3 (2.1)	0.39 (0.22)
>37.8 to ≤74.5	766	25.7 (4.5)	153.2 (6.5)		15.4 (3.0)	355 (46.3)	411 (53.7)	4 (3-5)		1.5 (2.2)	0.34 (0.23)
>74.5 to ≤1208.4	770	25.2 (4.6)	151.1 (6.1)		15.2 (3.1)	367 (47.7)	403 (52.3)	4 (3-5)		1.5 (2.2)	0.37 (0.23)

Abbreviations: GA, gestational age; PM_2.5_, fine particulate matter with a diameter less than or equal to 2.5 µm.

### Episodes of Severe Pneumonia During Infancy

There were 175 episodes of severe pneumonia during infancy (primary outcome) in 11 996 infant-quarters for a mean of 5.8 episodes for every 100 infant-years. Of the 160 infants with at least 1 episode of severe pneumonia, 147 (91.9%) had 1 episode, 12 (7.5%) had 2 episodes, and 1 (0.6%) had 4 episodes. We provide a summary of severe IMCI pneumonia episodes in eResults in Supplement 1. We summarized characteristics of severe pneumonia episodes during infancy in eTable 1 in Supplement 1. Infants with at least 1 episode of severe pneumonia had similar prenatal and postnatal exposures to PM_2.5_ as did infants without severe pneumonia (mean [SD] prenatal exposure: 101 [100] µg/m^3^ vs 88 [80] µg/m^3^; difference, 13 [95% CI, −3 to 29] µg/m^3^; *P* = .11; mean [SD] postnatal exposure: 70 [78] µg/m^3^ vs 67 [93] µg/m^3^; difference, 3 [95% CI, −11 to 17] µg/m^3^; *P* = .68). There were no discernable differences in the distributions of prenatal and postnatal exposures to PM_2.5_ by severe pneumonia status during infancy at any quarter (Figure).

**Figure.**
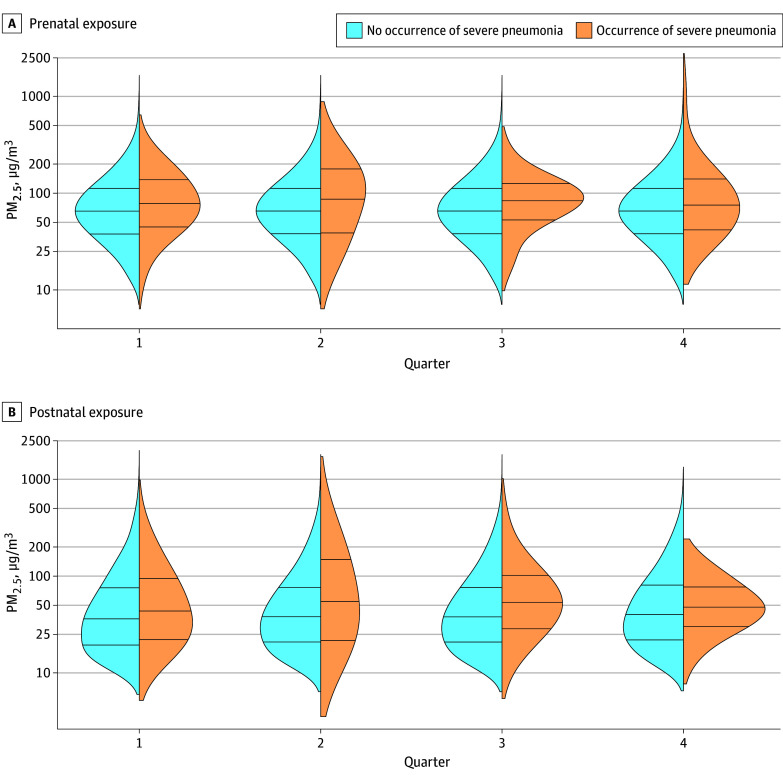
Density Plots of Prenatal and Postnatal Exposures to Fine Particulate Matter With a Diameter of Less Than or Equal to 2.5 µm (PM_2.5_) by Infant-Quarter and Severe Pneumonia Status During Infancy The horizontal lines inside the violin plots indicate the 25th, 50th, and 75th percentiles of personal exposures.

### Association Between PM_2.5_ Exposures and Severe Pneumonia During Infancy

A total of 170 infant-quarters of 11 996 had an occurrence of severe pneumonia during infancy. We plotted the risk of severe pneumonia during infancy per 100 infant-years by deciles of mean prenatal and postnatal exposures to PM_2.5_ in eFigure 2 in Supplement 1. While infants with higher prenatal and postnatal PM_2.5_ exposures showed a numerically greater risk of severe pneumonia, multivariable analyses did not identify an association between prenatal or postnatal PM_2.5_ exposures and severe pneumonia during infancy when assessed as continuous variables (prenatal: adjusted risk ratio [RR], 1.03; 95% CI, 0.94-1.13; postnatal: adjusted RR, 0.97; 95% CI, 0.87-1.09) (Table 2) or as categorical variables (Table 3). Subgroup analyses did not provide any evidence of effect modification by sex, winter season, socioeconomic wealth index, or PCV status (Table 4). In secondary analyses, we did not find an association between PM_2.5_ or CO exposures and severe IMCI infant pneumonia (eResults and eTable 2 in Supplement 1) or an association between CO exposures and severe pneumonia during infancy when evaluated as continuous (Table 2) or categorical (Table 3) variables. In sensitivity analysis, we did not find an association with either prenatal (adjusted hazard ratio, per change in IQR, 1.06; 95% CI, 0.97-1.15) or postnatal exposure to PM_2.5_ (adjusted hazard ratio per change in IQR, 0.97; 95% CI, 0.87-1.08) when using the Andersen-Gill counting process to account for multiple episodes of severe pneumonia during infancy.

**Table 2.** Unadjusted and Adjusted RRs for Personal Exposures to PM_2.5_ or CO and Severe Pneumonia During Infancy

Exposure	RR (95% CI)	
	Unadjusted	Adjusted^a^
Prenatal exposure to PM_2.5_, per IQR µg/m^3^	1.11 (1.02-1.20)	1.03 (0.94-1.13)
Postnatal exposure to PM_2.5_, per IQR µg/m^3^	1.03 (0.96-1.10)	0.97 (0.87-1.09)
Prenatal exposure to CO, per IQR ppm	0.94 (0.85-1.05)	1.02 (0.90-1.16)
Postnatal exposure to CO, per IQR ppm	0.89 (0.79-1.00)	0.92 (0.81-1.05)

Abbreviations: CO, carbon monoxide; PM_2.5_, fine particulate matter with a diameter less than or equal to 2.5 µm; RR, risk ratio.

^a^ Models were adjusted for quarter, quartiles of number of people sleeping in the house, sex, birth weight, an indicator for winter season, an indicator for the COVID-19 pandemic, quartiles of socioeconomic wealth index, indicator for any pneumococcal vaccination, and country. The interquartile values for prenatal exposure to PM_2.5_ were 39.5 µg/m^3^ for the 25th percentile and 108.8 µg/m^3^ for the 75th percentile; for postnatal exposure to PM_2.5_, 21.4 µg/m^3^ and 74.5 µg/m^3^; for prenatal exposure to CO, 0.53 parts per million (ppm) and 2.16 ppm; and for postnatal exposure to CO, 0.2 ppm and 1.75 ppm.

**Table 3.** Unadjusted and Adjusted RRs Between Quartiles of Personal Exposures to Air Pollutants and Severe Pneumonia During Infancy

Exposure	Incidence, episodes per 100 infant-years^a^	RR (95% CI)	
		Unadjusted	Adjusted^b^
**Prenatal exposure to PM_2.5_, µg/m^3^**			
≥10.7 to ≤39.5	3.78	1 [Reference]	1 [Reference]
>39.5 to ≤66.0	4.86	1.29 (0.79-2.13)	0.98 (0.59-1.65)
>66.0 to ≤109.0	6.21	1.64 (1.02-2.63)	1.11 (0.67-1.85)
>109.0 to ≤1090.0	7.97	2.10 (1.33-3.30)	1.33 (0.78-2.28)
**Postnatal exposure to PM_2.5_, µg/m^3^**			
≥9.8 to ≤21.4	3.54	1 [Reference]	1 [Reference]
>21.4 to ≤37.8	5.86	1.72 (1.06-2.78)	1.06 (0.62-1.80)
>37.8 to ≤74.5	6.28	1.83 (1.14-2.95)	0.91 (0.51-1.62)
>74.5 to ≤1208.4	6.82	1.97 (1.23-3.16)	0.91 (0.51-1.65)
**Prenatal exposure to CO, ppm**			
≥0 to 0.531	4.03	1 [Reference]	1 [Reference]
>0.531 to ≤1.070	8.20	2.09 (1.33-3.27)	1.93 (1.22-3.03)
>1.070 to ≤2.160	5.64	1.44 (0.89-2.34)	1.41 (0.85-2.32)
>2.160 to ≤46.400	4.97	1.25 (0.75-2.08)	1.31 (0.73-2.38)
**Postnatal exposure to CO, ppm**			
>0 to ≤0.204	5.42	1 [Reference]	1 [Reference]
>0.204 to ≤0.717	5.85	1.15 (0.76-1.75)	0.90 (0.59-1.37)
>0.717 to ≤1.750	6.96	1.37 (0.90-2.09)	1.10 (0.71-1.71)
>1.750 to 98.500	4.45	0.87 (0.54-1.39)	0.92 (0.54-1.59)

Abbreviations: CO, carbon monoxide; PM_2.5_, fine particulate matter with a diameter less than or equal to 2.5 µm; ppm, parts per million; RR, risk ratio.

^a^ The measure of 100 infant-years was approximated as 400 infant-quarters.

^b^ Models were adjusted for quarter, quartiles of number of people sleeping in the house, sex, birth weight, an indicator for winter season, an indicator for the COVID-19 pandemic, quartiles of socioeconomic wealth index, an indicator for any pneumococcal vaccination, and country.

**Table 4.** Effect Modification of the Association Between Personal Exposures to PM_2.5_ and Severe Pneumonia During Infancy by Sex, Winter Season, Socioeconomic Wealth Index, and Pneumococcal Conjugate Vaccine Status

Variable	Exposure to PM_2.5_, RR (95% CI)^a^	
	Prenatal	Postnatal
Sex		
Boys	1.07 (0.81-1.33)	0.84 (0.60-1.04)
Girls	1.02 (0.82-1.09)	1.05 (0.92-1.15)
Season		
Winter	0.88 (0.65-1.13)	1.05 (0.85-1.19)
Nonwinter	1.07 (0.94-1.16)	0.93 (0.71-1.06)
Socioeconomic Index		
≥Median	0.75 (0.43-1.01)	1.02 (0.67-1.20)
<Median	1.06 (0.92-1.16)	0.96 (0.82-1.08)
Pneumococcal conjugate vaccine status		
Received ≥1 doses	1.11 (0.90-1.20)	0.93 (0.78-1.06)
None	0.86 (0.64-1.04)	1.07 (0.80-1.28)

Abbreviations: PM_2.5_, fine particulate matter with a diameter less than or equal to 2.5 µm; RR, risk ratio.

^a^ RRs, scaled to the IQR, were adjusted for quarter, quartiles of number of people sleeping in the house, sex, birth weight, an indicator for winter season, an indicator for the COVID-19 pandemic, quartiles of socioeconomic wealth index, an indicator for any pneumococcal vaccination, and country.

## Discussion

We did not find an association between either prenatal or postnatal personal exposures to PM_2.5_ or CO and severe pneumonia during infancy, consistent with our intention-to-treat analysis.^16^ Our findings challenge the evidence generated by observational studies that have found strong associations between higher concentrations of household air pollution from solid fuel use and a greater risk of child pneumonia.

Although earlier studies have reported associations between categorical indicators of HAP exposure and LRIs in children,^8^ few have examined the exposure-response relationship using quantitative measures of exposure, and only one other study^34^ evaluated associations between prenatal and postnatal personal exposures to CO and severe pneumonia during infancy. An exposure-response analysis of a study conducted in Kenya^6^ modeled personal exposure to particles less than or equal to 10 µm in diameter (PM_10_) and found a higher odds of acute respiratory infections in children younger than 5 years across a range of 6 exposure categories ranging from less than 200 µg/m^3^ to greater than 3500 µg/m^3^. An exposure-response analysis of an RCT conducted in Guatemala^11^ found that a 50% exposure reduction in personal exposure to CO was associated with an 18% reduction in physician-diagnosed pneumonia and a 28% reduction in severe hypoxemic physician-diagnosed pneumonia in 518 children younger than 18 months^11^; however, these studies were conducted before the widespread use of *Haemophilus influenzae b* and pneumococcal vaccines. The third exposure-response analysis^10^ used data from an RCT conducted in Malawi and did not find an association between CO exposure and pneumonia in 1805 children younger than 5 years. Finally, an exposure-response analysis of the Ghana Randomized Air Pollution and Health Study (GRAPHS)^34^ found that a 1-ppm increase in prenatal exposure to CO was associated with a 10% and 15% higher risk of pneumonia and severe pneumonia, respectively, in 1141 infants. Consistent with our findings, GRAPHS did not find associations between either prenatal or postnatal PM_2.5_ exposures and severe pneumonia during infancy in a convenience sample of approximately 600 pregnant mothers who had PM_2.5_ exposures assessed during and after pregnancy.

A meaningful comparison of these previous studies with the findings from our current study requires consideration of differences in the exposure, outcome, target population, and sources of bias. The range of exposures compared is one important consideration, as the slope of the exposure-response association has been found to be shallower at higher exposure levels, eg, the risk ratio of severe pneumonia during infancy is less per unit of pollution when personal exposures to PM_2.5_ decrease from 200 µg/m^3^ to 100 µg/m^3^ than when they decrease from 60 µg/m^3^ to 30 µg/m^3^.^6,11,34^ This can explain the lack of association in the Malawi study, in which the intervention was not effective at reducing exposure, but it does not explain the lack of an association in our current study, in which the LPG intervention created a strong contrast in personal PM_2.5_ exposures over 24 hours,^14,15^ ranging from 5.4 µg/m^3^ to 1182 µg/m^3^. Regarding the definition used for severe pneumonia, previous studies show that the effects of HAP may be greater for more severe forms of lower respiratory disease.^11,34^ A lack of association in our current study is not explained by inclusion of nonsevere disease because our case definitions were specific for severe pneumonia during infancy. When comparing the populations studied, the landscape of risk factors of pneumonia and etiological causes of pneumonia has changed over the time when these studies were conducted.^2^ In 1990, when the first studies linking HAP exposures to pneumonia were conducted, pneumonia in children younger than 5 years was responsible for 1.9 million deaths per year.^2^ In 2021, annual deaths from pneumonia in children younger than 5 years decreased to 502 000,^2^ largely thanks to immunization programs against common bacteria like *Streptococcus pneumoniae* and *Hemophilus influenzae*.

The heterogeneity in findings from exposure-response studies of HAP and pneumonia in LMICs may be partly due to differences in bias. Our study design and conduct addressed several of these potential biases. First, confounding—either because of unmeasured variables or inadequate adjustment—is a major concern when studying the association between HAP exposure and pneumonia in observational studies, particularly among those that did not adequately control for socioeconomic status.^6^ To minimize confounding, we conducted a careful evaluation of potential confounders collected in our study using a directed acyclic graph. Second, we measured personal exposure using a wearable device that measured PM_2.5_ both gravimetrically and nephelometrically. PM_2.5_ is generally considered to represent the health-damaging constituents of HAP^35^; however, earlier studies were only able to evaluate associations between CO concentrations and infant pneumonia.^11,34^ The only previous study to analyze the exposure-response association with particles was based on modeling of kitchen concentrations and reported activity patterns and included coarser particles that are less likely to impact the lower airways.^6^ Third, our study required cases to either have consolidation on imaging or hypoxemia by pulse oximetry, both of which are signs specific to pneumonia. None of the earlier studies included imaging as part of the primary end point, and 1 study^11^ that included chest radiography in secondary outcomes had about 20% of missing images. GRAPHS did not include imaging as part of their case definition, and while hypoxemia was used, their case definition did not require its presence. Furthermore, most cases of severe pneumonia in GRAPHS had chest indrawing as the only criterion for severity, which has not been considered a danger sign by the WHO since 2014, and when it is identified in isolation, it has a very low risk of mortality.^25^ Previous research demonstrated that IMCI guidelines have low specificity, with only approximately 50% of severe cases confirmed to have lower respiratory disease. It is therefore likely that a high proportion of infants in GRAPHS did not have lower respiratory disease consistent with severe pneumonia. Our definition of severe pneumonia during infancy was both highly sensitive and specific, making misclassification of severe pneumonia unlikely among those we screened. Finally, our study population is likely more representative of the target population of residents in low- and middle-income countries where biomass fuels are used today, whereas earlier studies included participants from only one study setting.

### Limitations

Our study has limitations. First, despite our careful evaluation of causal assumptions, our study may have residual confounding due to unmeasured, poorly measured, or mismodeled confounders. The intervention may have also influenced pneumonia risk through other pathways, such as changes in disposable income, food quality, or fuel collection and cooking time; however, data on these factors were not collected. Second, exposure measurement error is another concern because we had a small number of repeated measures per child and we estimated infant exposures indirectly using a method only validated in adults.^19,36^ Third, the LPG stove in our study was unvented, and we cannot rule out the possibility of toxic effects resulting from LPG combustion.^37^ Fourth, we may have failed to identify all cases of severe pneumonia that did not seek care or reach one of the sentinel health care facilities. Our surveillance was limited to public, government-run health care facilities in our study area. This approach may have resulted in missed cases if infants were taken to private clinics or hospitals. Additionally, surveillance could not be conducted 24 hours a day, 7 days a week at some health care facilities, and we faced restrictions in access during the COVID-19 pandemic; however, we asked about any hospital visits for severe pneumonia during quarterly home visits and conducted verbal autopsies of any infant deaths to identify missed cases. Medical records were often poor, limiting data abstraction when our research staff could not directly evaluate the infant. We previously discussed the advantages and disadvantages of active home surveillance vs active hospital surveillance for severe pneumonia during infancy.^21,22^ While low health care seeking may affect identification of severe pneumonia cases,^38^ we opted for health care facility surveillance because home surveillance may lead to the identification of less severe LRIs. Fifth, we cannot rule out the possibility of differential case ascertainment in an unblinded intervention; however, our research staff determined case status without knowledge of the intervention arm status. Sixth, our findings may also be affected by the overall low incidence of severe pneumonia during infancy in our population. The COVID-19 pandemic started midway through our trial, affecting the overall incidence of severe pneumonia during infancy and our ability to actively ascertain cases in our study settings. Additionally, we cannot rule out the possibility that our study was underpowered to detect an association between household air pollutants and severe pneumonia in infants when controlling for multiple factors, as the HAPIN trial was powered around the intention-to-treat analysis.

## Conclusions

In conclusion, our study did not provide evidence of an association between personal exposures to PM_2.5_ or CO and severe pneumonia during infancy. Taken together with recent intention-to-treat analyses,^9,16^ our results suggest that neither PM_2.5_ nor CO exposures from biomass cooking is a strong risk factor for severe pneumonia during infancy in low- and middle-income countries, and household-level mitigation strategies to reduce these pollutants with cleaner cooking are unlikely to have a meaningful impact on severe pneumonia during infancy.

## Notes

Supplement 1.
eMethods.

eResults.

eReferences.
**eTable 1.** Demographic and Clinical Characteristics of Severe Pneumonia and World Health Organization Severe Integrated Management of Childhood Illnesses Pneumonia Episodes During Infancy **eTable 2.** Unadjusted and Adjusted Risk Ratios Between Personal Exposures to PM_2.5_ and Severe World Health Organization Integrated Management of Childhood Illnesses Pneumonia During Infancy **eFigure 1.** Correlation Matrix of Prenatal and Postnatal Exposures to Fine Particulate Matter, Measured 3 Times During Pregnancy and 3 Times During Infancy **eFigure 2.** Risk of Severe Pneumonia During Infancy by Deciles of Prenatal and Postnatal Exposures to Fine Particulate Matter **eAppendix.** R Statistical Code

Supplement 2.
Nonauthor Collaborators

Supplement 3.
Data Sharing Statement
