# Supplementary material for: Exposure to Household Air Pollution From Biomass Cooking and Severe Pneumonia in Infants
Source: JAMA Netw Open. 2025 Oct 29;8(10):e2538721. doi: 10.1001/jamanetworkopen.2025.38721 (PMC12573034; doi:10.1001/jamanetworkopen.2025.38721)
Supplement: Supplement 1. — eMethods. eResults. eReferences. eTable 1. Demographic and Clinical Characteristics of Severe Pneumonia and World Health Organization Severe Integrated Management of Childhood Illnesses Pneumonia Episodes During Infancy eTable 2. Unadjusted and Adjusted Risk Ratios Between Personal Exposures to PM2.5 and Severe World Health Organization Integrated Management of Childhood Illnesses Pneumonia During Infancy eFigure 1. Correlation Matrix of Prenatal and Postnatal Exposures to Fine Particulate Matter, Measured 3 Times During Pregnancy and 3 Times During Infancy eFigure 2. Risk of Severe Pneumonia During Infancy by Deciles of Prenatal and Postnatal Exposures to Fine Particulate Matter eAppendix. R Statistical Code [file jamanetwopen-e2538721-s001.pdf]

# Supplemental Online Content

McCracken JP, McCollum ED, Steenland K, et al. Exposure to household pollution from biomass cooking and severe pneumonia in infants. *JAMA Netw Open*. 2025;8(10):e2538721. doi:10.1001/jamanetworkopen.2025.38721

**eMethods.**
**eResults.**
**eReferences.**
**eTable 1.** Demographic and Clinical Characteristics of Severe Pneumonia and World Health Organization Severe Integrated Management of Childhood Illnesses Pneumonia Episodes During Infancy
**eTable 2.** Unadjusted and Adjusted Risk Ratios Between Personal Exposures to PM2.5 and Severe World Health Organization Integrated Management of Childhood Illnesses Pneumonia During Infancy
**eFigure 1.** Correlation Matrix of Prenatal and Postnatal Exposures to Fine Particulate Matter, Measured 3 Times During Pregnancy and 3 Times During Infancy
**eFigure 2.** Risk of Severe Pneumonia During Infancy by Deciles of Prenatal and Postnatal Exposures to Fine Particulate Matter
**eAppendix.** R Statistical Code

This supplemental material has been provided by the authors to give readers additional information about their work.

© 2025 McCracken JP et al. *JAMA Network Open*.

**eMethods**

**Description of active case surveillance at health facilitiies**

A detailed description of the active health services surveillance was provided elsewhere (1). The facility-based surveillance involved a three-tiered approach. First, mothers/caregivers were asked to alert research staff by telephone if medical care was sought for the infant for any reason. We set up a telephone system monitored by our research staff twenty-four hours a day, seven days a week for this notification process. As a second-line strategy, on-site surveillance at sentinel hospitals was conducted by research staff in case mothers or caregivers did not notify them before the infant arrived at the hospital. Research staff were physically present at the sentinel facilities either during working hours and on-call after hours (Peru, Rwanda and selected facilities in India) or they were present 24 hours a day, seven days a week (Guatemala, selected facilities in India). Research staff worked with hospital staff to identify infants who sought care at their facility, including checking the rosters in Emergency Departments and inpatient wards. We also provided mothers or caregivers with items such as hats, bags or identification cards to help health care facility staff identify infants and study participants. During hours where research staff were not physically present at the health care facility, on-call research staff members were available once notified by the caregiver, mother, or facility personnel that an infant sought care. Emergency Department staff at each health care facility were also asked to notify on-call research staff by telephone when a study infant presented to their health care facility for any illness using the above-mentioned telephone system.

Infants presenting at sentinel health care facilities for any illness were evaluated by research staff trained and certified to conduct a respiratory examination according to a standard assessment procedure applied at all four study sites within twenty-four hours of arrival. Research staff were also trained not to interfere with usual care but interacted with staff at the health care facility about the infant's clinical diagnosis and treatment to enrich data collection. Research staff were blinded to randomization arm. We asked research staff to complete a data collection survey which was uniformly applied across countries. The admission examination and overall hospital course was also abstracted from the medical charts to assess key elements to the infant's presentation and hospitalization.

As a third line strategy, home visits by research staff were made at one, three, six, nine, and 12 months of age to identify potential gaps in our surveillance system. If the infant had a respiratory illness and/or visited any health care facility for any illness since the last home visit based on caregiver recall, research staff recorded the name of the health care facility and date, if known. Research staff then visited the health care facility and completed a chart review form to identify key variables such as oxygen saturation levels and obtain any available chest radiograph imaging. If the infant was ill during this home visit, the research staff were instructed to refer the infant to a health care facility.

**General and neonatal danger signs**

We followed recommendations from the World Health Organization (WHO) Integrated Management of Childhood Illnesses (IMCI) to identify danger signs (2). General danger signs include the inability to drink or breast-feed, convulsions, stridor at rest, lethargy, unconsciousness, or vomiting all ingested food, fluid, and medications. Neonatal danger signs include inability to feed well, not moving at all or movement only when stimulated, grunting, or severe indrawing of the lower chest wall.

**Methods for personal exposure assessment**

We used the Enhanced Children's MicroPEM (ECM, RTI International, Research Triangle Park, NC, USA) to measure both mass-based (gravimetric) and direct-read (nephelometric) $PM_{2.5}$ concentrations. To

© 2025 McCracken JP et al. *JAMA Network Open*.

measure mass, ECM draws in air continuously through a pump at 0·3 L/min; particles are separated by a 2·5 µm impactor and deposited into a pre-weighed 15-mm polytetrafluoroethylene filter (PT15-AN-PF02, MTL LLC., Minneapolis, MN, USA). We collected an additional 659 blank filters for blank-correction and 859 valid duplicate filters for measuring reliability. We set the ECM to direct read and log estimates for $PM_{2·5}$ concentrations every 10 seconds. We used the Lascar EL-USB-CO monitor (Lascar Electronics, Erie, PA, USA) to log CO concentrations in 1-minute intervals estimated using an electrochemical sensor.

Filters were pre- and post-weighed for $PM_{2·5}$ using microbalances (Sartorius Cubis, MSA6.6s-000-DF, Göttingen, Germany) with 1 µg precision at the University of Georgia (Guatemala, Peru and Rwanda) and at the Sri Ramachandra Institute for Higher Education and Research (India). We calculated mass-based personal exposures to $PM_{2·5}$ as the difference between post- and pre-weights corrected by the median of field blanks. We then corrected all direct-read measurements of $PM_{2·5}$ using mass-based concentrations.

**Variable definitions**

We defined winter season as November through February for Guatemala and India, and May through August for Peru (Rwanda had no assigned winter months); the COVID-19 pandemic as period starting on March 15, 2020; and pneumooccal vaccination as receiving at least one dose of a pneumococcal vaccine.

**Estimation of a socioeconomic wealth index**

We calculated a socioeconomic status index (based on self-reported indicators such as improved housing materials, access to improved water and sanitation, and ownership of durable assets like televisions, refrigerators, and computers) using iterative imputation for missing data and factor analysis for mixed data[3], where higher values indicate a higher socioeconomic status (we scaled the first component from 0 to 1, with a median of 0.41 and a range from 0.12 to 0.74).

**Directed acyclic graph**

We plotted the directed acyclic graph (DAG) for effect of household air pollution exposure on severe pneumonia within a specific country below. Country indicator variables will be added to any adjustment set. Potential confounders were selected from literature and knowledge of the study design.

The variables were first placed in temporal order from left to right to clarify the possible causal relationships. The coauthors reached a consensus on the direct causal relations that are assumed to exist (arrow between nodes) and where the causal null hypothesis can be assumed (no arrow between a node and any future node). Green arrows represent the causal relationship of interest, and pink arrows represent biasing pathways. Pink nodes are ancestors of exposure and outcome. An empty blue node is an ancestor of the outcome. A grey node (H) means that this variable is unmeasured.

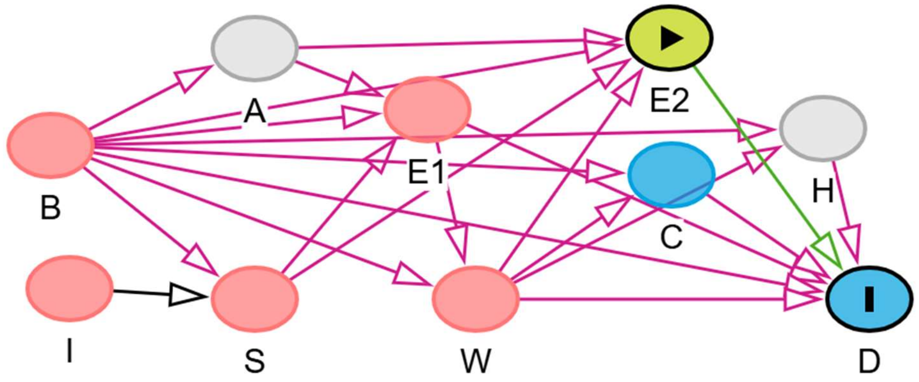


© 2025 McCracken JP et al. *JAMA Network Open*.

| Node label | Variables |
|---|---|
| B | Baseline confounders: Follow-up start time, Sex, Elevation, Population density, Household members (number, age, and sex), Crowding, Secondhand smoke, Electricity, Maternal Vaccination |
| I | Randomized liquified petroleum gas cooking intervention |
| S | Stove and fuel used for cooking |
| A | Ambient air pollution |
| E1 | Prenatal pollutant exposure ($PM_{2.5}$, BC, or CO) |
| W | Birth weight and gestational age |
| E2 | Post-natal pollutant exposure ($PM_{2.5}$, BC, or CO) |
| H | Healthcare seeking |
| C | Childhood risk factors: breastfeeding, nutrition, vaccination, and WASH (water, sanitation and hygiene) |
| D | Disease: Severe pneumonia in infants |

The DAG implies one minimal adjustment set for the effect of prenatal exposure on severe pneumonia in infants: {B, A, S}. A (ambient air pollution) is unmeasured, which indicates that the analysis cannot distinguish the effects of household and ambient sources of air pollution exposure. The DAG implies one minimal adjustment set for the effect of postnatal exposure on severe pneumonia: {B, E1, W}.

## eResults

### Characteristics of severe pneumonia in infants

We summarized demographics, clinical characteristics and outcomes of severe infant pneumonia episodes in Table S1.

### Personal exposure assessments

The Spearman correlation between personal exposures to $PM_{2.5}$ obtained from the mother and the infant personal exposure in the same visit was 0.89 (95% CI 0.88 to 0.90). We also observed moderate correlations ranging from 0.44 to 0.52 between prenatal and post-natal exposures to $PM_{2.5}$ (E-Figure 1).

### Relationship between $PM_{2.5}$ exposures and severe pneumonia in infants

A total of 170 infant-quarters out of 11,996 had an occurrence of severe pneumonia. We plotted the risk of severe pneumonia per 100 infant-years by deciles of mean prenatal and post-natal exposures to $PM_{2.5}$ in E-Figure 2. Infants with higher prenatal and postnatal $PM_{2.5}$ exposures showed a numerically greater risk of severe pneumonia.

### Episodes of severe IMCI pneumonia in infants

There were 267 episodes of severe Integrated Management of Childhood Illness (IMCI) pneumonia during infancy (secondary outcome) in 11,996 infant-quarters for a mean of 8.9 episodes for every 100 infant-years. Of 260 infants with at least one episode of severe IMCI pneumonia, 226 infants had one episode, 17 infants had two, one infant had three and one infant had four. We summarized demographics, clinical characteristics and outcomes of severe IMCI pneumonia episodes during infancy in E-Table 1. Infants with at least one episode of severe IMCI pneumonia had higher prenatal (103 vs. 87 $\mu g/m^3$; p=0.03) but similar post-natal exposures (71 vs. 67 $\mu g/m^3$; p=0.59) to $PM_{2.5}$ when compared to the exposures in infants without severe IMCI pneumonia.

### Relationship between $PM_{2.5}$ exposures and severe IMCI pneumonia in infants

A total of 260 infant-quarters out of 11,996 had an occurrence of severe IMCI pneumonia in infants. In multivariable analyses, we did not find an association between prenatal or post-natal exposures to $PM_{2.5}$ or CO and severe IMCI pneumonia during infancy (E-Table 2).

© 2025 McCracken JP et al. *JAMA Network Open*.

**eReferences**

1. Simkovich SM, Underhill LJ, Kirby MA, Goodman D, Crocker ME, Hossen S, McCracken JP, de León O, Thompson LM, Garg SS, Balakrishnan K, Thangavel G, Rosa G, Peel JL, Clasen TF, McCollum ED, Checkley W. Design and conduct of facility-based surveillance for severe childhood pneumonia in the Household Air Pollution Intervention Network (HAPIN) trial. ERJ Open Res 2020; 6(1):00308-2019.
2. World Health Organization. Integrated Management of Childhood Illness: Chart Booklet. 2014.
3. Audigier V, Husson F, Josse J. A principal component method to impute missing values for mixed data. *Adv Data Anal Classif.* 2016;10(1):5–26.

© 2025 McCracken JP et al. *JAMA Network Open*.

**eTable 1. Demographic and clinical characteristics of severe pneumonia and World Health Organization (WHO) severe Integrated Management of Childhood Illnesses (IMCI) pneumonia episodes during infancy.**

| | | Severe pneumonia in infants (n=175) | Severe IMCI pneumonia in infants (n=267) |
|---|---|---|---|
| **Country** | | | |
| | Guatemala | 70 (40) | 106 (40) |
| | India | 11 (6) | 34 (13) |
| | Peru | 13 (7) | 19 (7) |
| | Rwanda | 81 (46) | 108 (40) |
| Age at first episode, mean (SD) | | 3.9 (3.9), n=160 | 2.7 (3.5), n=245 |
| Age at all episodes, mean (SD) | | 4.2 (4.0) | 3.1 (3.6) |
| **Clinical characteristics** | | | |
| | Temperature in °C at episode, mean (SD) | 37.4 (1.0), n=166 | 37.2 (1.1), n=259 |
| | Temperature > 38°C at episode, n/N (%) | 75/166 (45) | 102/259 (39) |
| | Respiratory rate for infants < 2 months, mean (SD) | 61.8 (15.6), n=69 | 59.7 (15.0), n=147 |
| | Respiratory rate for infants 2-12 months, mean (SD) | 56.5 (12.4), n=94 | 54.4 (12.0), n=105 |
| | Respiratory danger sign, n (%) | 122/156 (78) | 139/248 (56) |
| | Oxyhemoglobin saturation in % for episodes at < 2500 m, mean (SD) | 88.4 (6.2), n=137 | 92.3 (6.7), n=217 |
| | Oxyhemoglobin saturation in % for episodes at ≥ 2500 m, mean (SD) | 80.1 (5.3), n=8 | 83.7 (6.3), n=19 |
| | Met criteria for hypoxemia, n/N (%) | 141/166 (85) | 114/264 (43) |
| | Mer criteria for consolidation by imaging, n/N (%) | 88/136 (65) | 86/184 (47) |
| **Clinical outcomes** | | | |
| | Hospitalized, n/N (%) | 90/165 (54) | 147/264 (56) |
| | Received oxygen, n/N (%) | 54/166 (33) | 74/265 (28) |
| | Advanced respiratory supportive care, n/N (%) | 23/166 (14) | 18/265 (7) |
| | Death, n/N (%) | 10/175 (6) | 10/267 (4) |

© 2025 McCracken JP et al. *JAMA Network Open*.

**eTable 2. Unadjusted and adjusted risk ratios (RR, scaled to the interquartile range [IQR]) between personal exposures to $PM_{2.5}$ and severe World Health Organization Integrated Management of Childhood Illness (IMCI) pneumonia during infancy. We adjusted for quarter, quartiles of number of people sleeping in the house, sex, birthweight, an indicator for winter season, an indicator for the COVID-19 pandemic, quartiles of socioeconomic wealth index, indicator for any pneumococcal vaccination and country. The interquartile values for prenatal exposure to $PM_{2.5}$ were 39.5 μg/m$^3$ for the 25$^{th}$ percentile and 108.8 μg/m$^3$ for the 75$^{th}$ percentile; for postnatal exposure to $PM_{2.5}$ were 21.4 μg/m$^3$ for the 25$^{th}$ percentile and 74.5 μg/m$^3$ for the 75$^{th}$ percentile; for prenatal exposure to CO were 0.53 ppm for the 25$^{th}$ percentile and 2.16 ppm for the 75$^{th}$ percentile; and for postnatal exposure to CO were 0.2 ppm for the 25$^{th}$ percentile and 1.75 ppm were for the 75$^{th}$ percentile.**

| | | Unadjusted analysis | | Adjusted analysis | |
|---|---|---|---|---|---|
| **Severe IMCI infant pneumonia** | | **RR** | **95% CI** | **RR** | **95% CI** |
| | Prenatal exposure to $PM_{2.5}$ (per IQR μg/m$^3$) | 1.12 | 1.05 – 1.19 | 1.04 | 0.97 – 1.12 |
| | Post-natal exposure to $PM_{2.5}$ (per IQR μg/m$^3$) | 1.06 | 1.00 – 1.11 | 1.01 | 0.93 – 1.09 |
| | Prenatal exposure to CO (per IQR ppm) | 0.98 | 0.91 – 1.04 | 1.03 | 0.95 – 1.11 |
| | Post-natal exposure to CO (per IQR ppm) | 0.96 | 0.89 – 1.03 | 0.99 | 0.93 – 1.05 |

© 2025 McCracken JP et al. *JAMA Network Open*.

**eFigure 1. Correlation matrix of prenatal and post-natal exposures to fine particulate matter ($PM_{2.5}$) measured three times during pregnancy (at baseline [BL] and at 24–48 [P1] and 32–36 weeks [P2] of gestation) and three times during infancy (at ages 3 [B1], 6 [B2] and 12 [B4] months).** We display Spearman correlations for pairs of prenatal (BL, P1, P2) and post-natal (B1, B2, B4) $PM_{2.5}$ exposures by visit. Positive correlations are colored in blue, and the intensity of blue coloring determines the strength of the positive correlation. Negative correlations are colored in red, and the intensity of red coloring determines the strength of the negative correlation. The shape of the ellipses has the eccentricity parametrically scaled to the correlation value. We also display the Spearman correlation values for each pair inside the ellipse.

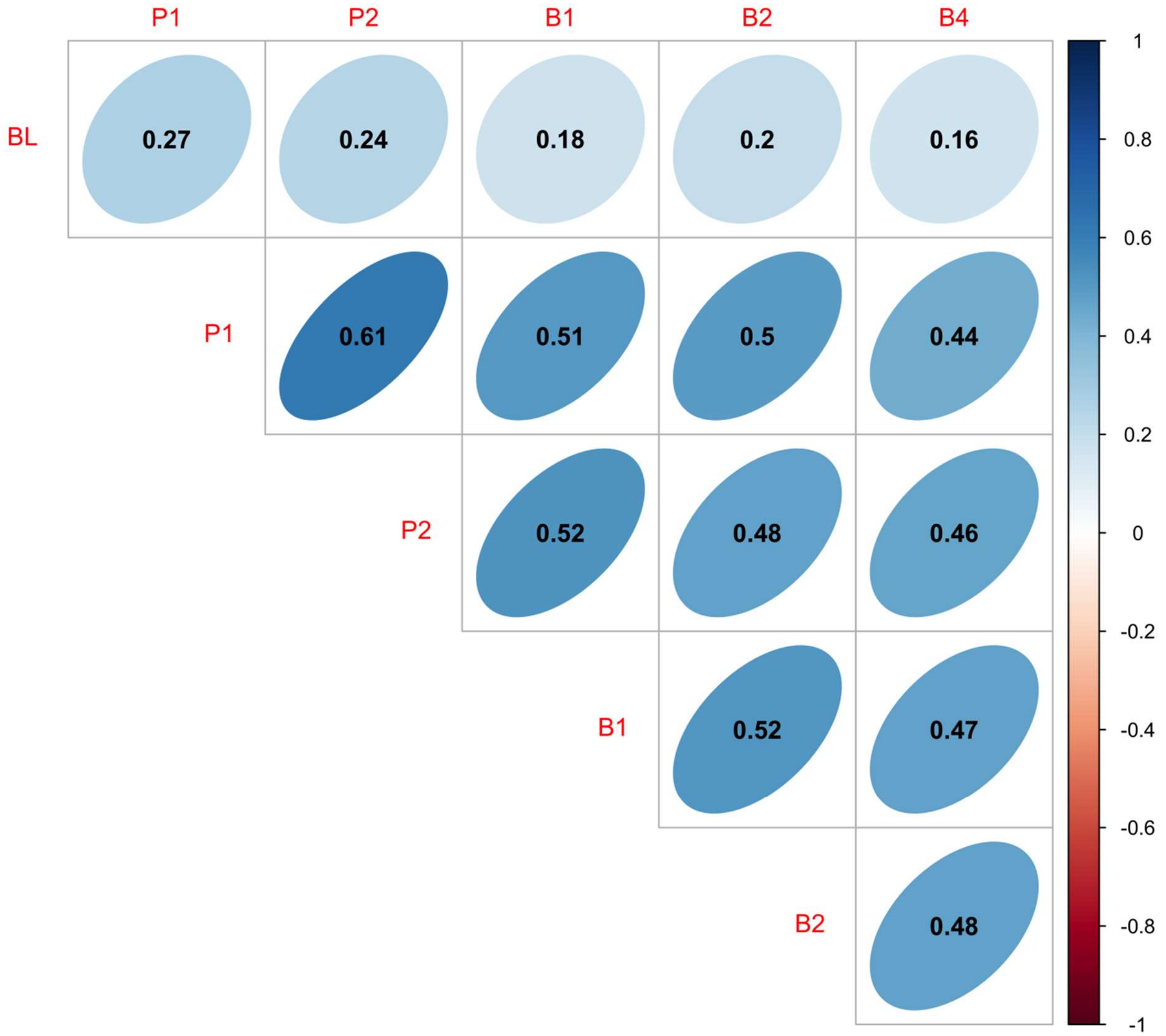


© 2025 McCracken JP et al. *JAMA Network Open*.

**eFigure 2. Risk of severe pneumonia during infancy by deciles of prenatal and post-natal exposures to fine particulate matter ($PM_{2.5}$).** The risk of severe pneumonia during infancy was calculated as occurrences of severe pneumonia during infancy by infant-quarter (scaled to 100 infant-years). The risk of severe pneumonia during infancy by deciles of prenatal exposures are plotted on the left panel, and the risk of severe pneumonia during infancy by deciles of post-natal exposures on the right panel.

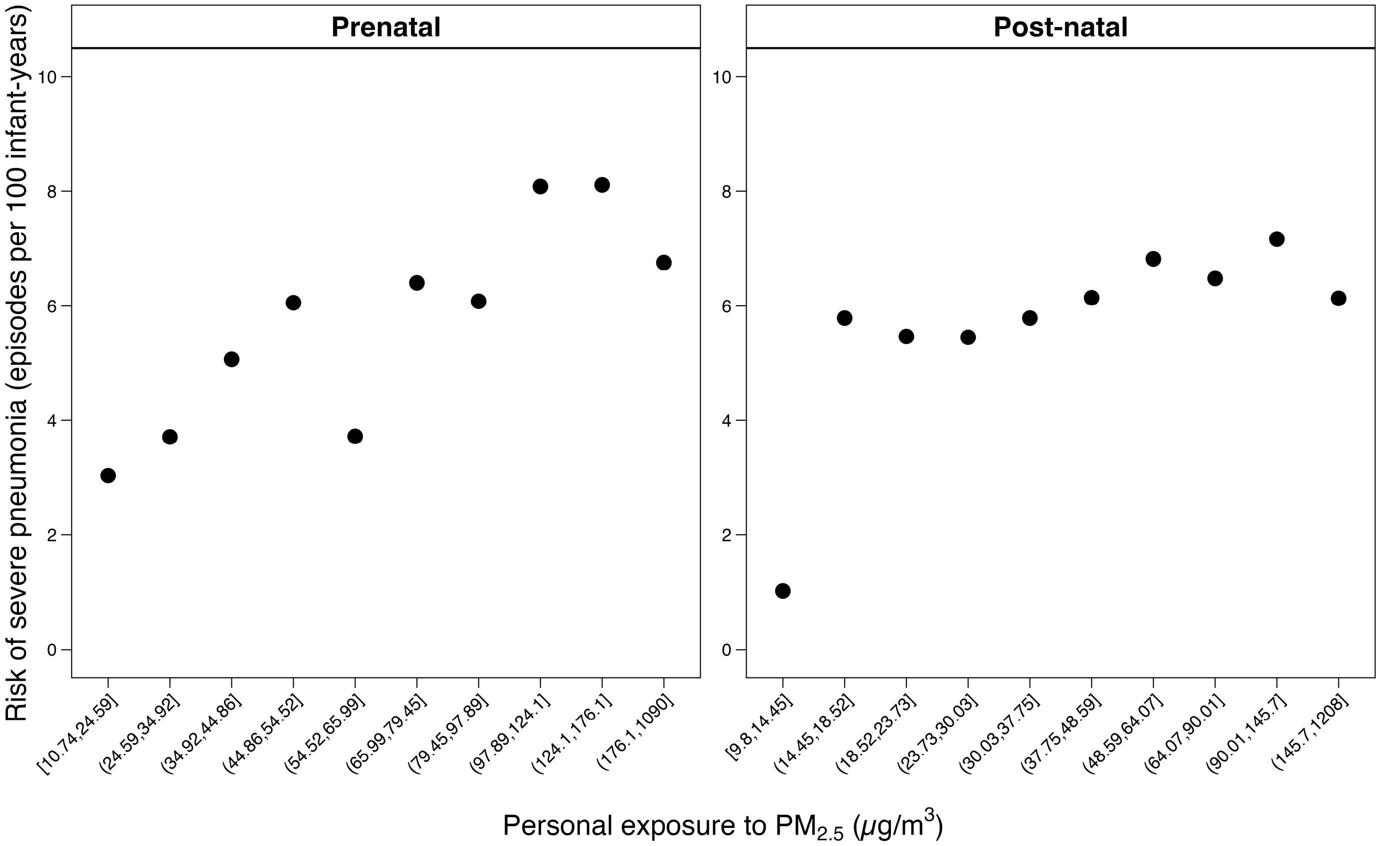


© 2025 McCracken JP et al. *JAMA Network Open*.

**eAppendix. R Statistical Code**

```
### William Checkley 28DEC2024, updated 17JUL2025
### Data management and analysis
### Analyses conducted by William Checkley and Kyle Steenland
### Code reviewed by Mingling Yang
### Main analysis conducted in R 4.2.2, replication analysis in SAS 9.4

## Load libraries
library(gee)
library(glmtoolbox)
library(haven)
library(RVAideMemoire)
library(corrplot)
library(dplyr)
library(survival)

## Read databases

# set working directory
# setwd("your/path/here")

# Read in ER-3 database
# Renamed ER3_hapinseverepneum_20230728_unfmt_ norepeat-withwhosever-
 corrected.csv as zer3
er3=read.csv("zer3.csv")

# Read in HAP exposures in mom, child, OAW
# Renamed hapin_ecm_all_20231221_fmt as hap
hap=read.csv("hap.csv")

# Read in socioeconomic wealth index
# Developed by Laura Nicolaou, Mingling Yang and William Checkley
# Code available from Mingling Yang, ses.csv file provided by Mingling Yang
ses=read.csv("ses.csv")

# Read in extra variables not provided by Data Management Core
hapinxtra=read.csv("hapinxtra.csv")

## Data Management

# Select a subset of HAP data
# subset data to mother and child data only
hap2 = subset (hap, select=c(HHID, IRC, s6_arm, timepoint, ecm_date_m,
 ECM_grav_neph_conc_M, ECM_valid_pm_M, ecm_date_c, ECM_grav_neph_conc_C,
 ecm_valid_pm_C, CO_avg_ppm_M, CO_valid_M, CO_avg_ppm_C, CO_valid_C))

# Rename variables for analysis
names(hap2)=c("hhid","irc","lpg","visit","mompm_date","mompm","mompm_valid","
 childpm_date","childpm","childpm_valid","momco","momco_valid","childco","chil
 dco_valid")

# N.B. All non-valid personal exposure assessments were converted to NA by
 the Data Management Center

# Convert study arm into 1/0 (lpg/control)
hap2$lpg=ifelse(as.character(hap2$lpg)=="Control",0,1)
```

© 2025 McCracken JP et al. *JAMA Network Open*.

```
# If child PM/CO data is missing, replace with mom PM/CO
# echildpm = combines prenatal exposures collected in pregnant mother with
# indirect post-natal child exposures. If indirect post-natal child exposures
# are missing we use the mother’s personal exposure
hap2$echildpm_date =  ifelse(is.na(hap2$childpm)==T,
                      hap2$mompm_date, hap2$childpm_date)
hap2$echildpm = ifelse(is.na(hap2$childpm)==T, hap2$mompm, hap2$childpm)
hap2$echildco = ifelse(is.na(hap2$childco)==T, hap2$momco, hap2$childco)

# Remove subset of participants with additional measurements
hap2 = hap2 [ nchar(hap2$visit)==2,]

# Turn date in HAP into R date format
# Dates in HAP were formatted as YYYY-MM-DD
hap2$echildpm_date = as.Date(hap2$echildpm_date)

# hap2 has the following variables:
# hhid                Household id
# irc                 International Research Center Guatemala,
#                     India, Peru, Rwanda
# visit               Visit BL, P1, P2, B1, B2, B4
# lpg                 Intervention arm, 1 Intervention, 0 Control
# mompm_date          Date of mother's personal exposure to PM assessment
# mompm               Mother's personal exposure to PM
# mompm_valid         If mother's personal exposure to PM is valid (1 valid,
#                     0 non  valid). If not valid mompm NA
# childpm_date        Date of child's personal exposure to PM assessment
# childpm             Child's personal exposure to PM
# childpm_valid       If child's personal exposure to PM is valid (1 valid, 0
#                     non-valid). If not valid mompm NA
# echildpm_date       Date of PM for combined child/mother PM
# echildpm            Personal exposure to PM (if child's missing, use
#                     mother's)
# momco               Mother's personal exposure to CO
# momco_valid         If child's personal exposure to CO is valid (1 valid,
#                     0 non-valid). If not valid momco NA
# childco             Child's personal exposure to CO
# childco_valid       If child's personal exposure to CO is valid (1 valid, 0
#                     non-valid). If not valid childco NA
# echildco            Personal exposure to CO (if child's missing, use
#                     mother's)

## Working with ER-3 (data by infant-quarter)
# Select a subset of ER-3 data sent by Data Management Center

xer3 = subset (er3, select = c(hhid, quarter, start, stop, HE3_pneumonia,
 HE3_IMCI_Severe, winter, COVID, NumDose_PCV_Pnm_cum, irc))

# Rename variables in ER-3 data
names(xer3)[5] = "pneumonia"
names(xer3)[6] = "whosevere"
names(xer3)[10] = "pcvdoses"

# Simplify pneumococcal vaccination variable as 0/1 (never/1+ doses)
xer3$pcv = ifelse(as.numeric(xer3$pcvdoses) >=1, 1, 0)
```

© 2025 McCracken JP et al. *JAMA Network Open*.

```
# Convert pneumonia outcomes to 0/1 (No/Yes)
# Original database has responses as Yes and No
# Revised dataset provided by Data Coordinating Center coded it as 0/1
# Below code is not needed unless formatting is reverted to original
## xer3$pneumonia = ifelse(xer3$pneumonia=="Yes",1,0)
## xer3$whosevere = ifelse(xer3$pneumonia=="Yes",1,0)

# Turn dates in ER-3 to R date variables
# Dates in ER-3 were formatted as MM-DD-YYYY
xer3$start = as.Date(xer3$start,format="%m/%d/%Y")
xer3$stop = as.Date(xer3$stop,format="%m/%d/%Y")

# Create a wide HAP dataset for prenatal and post-natal PM
# Study arm by infant
lpg = aggregate (lpg ~ hhid, data = hap2, FUN=max)

# Country by infant
irc = aggregate (irc ~ hhid, data = hap2, FUN=unique)

# Merging personal exposures by visit
# BL = baseline
# P1 = first measurement during pregnancy at 24-28 weeks
# P2 = second measurements during pregnancy at 32-36 weeks
# B1 = first personal exposure assessment when infant is 3 months old
# B2 = second personal exposure assessment when infant is 6 months old
# B4 = first personal exposure assessment when infant is 12 months old

blhap2 = subset(hap2, select=c(      hhid,
                                     echildpm_date,
                                     echildpm, echildco))[hap2$visit=="BL",]
p1hap2 = subset(hap2, select=c(      hhid,
                                     echildpm_date,
                                     echildpm,
                                     echildco))[hap2$visit=="P1",]
p2hap2 = subset(hap2, select=c(      hhid,
                                     echildpm_date,
                                     echildpm,
                                     echildco))[hap2$visit=="P2",]
b1hap2 = subset(hap2, select=c(      hhid,
                                     echildpm_date,
                                     echildpm,
                                     echildco))[hap2$visit=="B1",]
b2hap2 = subset(hap2, select=c(      hhid,
                                     echildpm_date,
                                     echildpm,
                                     echildco))[hap2$visit=="B2",]
b4hap2 = subset(hap2, select=c(      hhid,
                                     echildpm_date,
                                     echildpm,
                                     echildco))[hap2$visit=="B4",]

names(blhap2)=c("hhid","bldate","blpm","blco")
names(p1hap2)=c("hhid", "p1date","p1pm","p1co")
names(p2hap2)=c("hhid", "p2date","p2pm","p2co")
names(b1hap2)=c("hhid", "b1date","b1pm","b1co")
names(b2hap2)=c("hhid", "b2date","b2pm","b2co")
names(b4hap2)=c("hhid", "b4date","b4pm","b4co")
```

```
xhap2 = merge(lpg, irc, by="hhid",all=T)
xhap2 = merge(xhap2, blhap2, by="hhid",all=T)
xhap2 = merge(xhap2, p1hap2, by="hhid",all=T)
xhap2 = merge(xhap2, p2hap2, by="hhid",all=T)
xhap2 = merge(xhap2, b1hap2, by="hhid",all=T)
xhap2 = merge(xhap2, b2hap2, by="hhid",all=T)
xhap2 = merge(xhap2, b4hap2, by="hhid",all=T)

# write.csv(xhap2, "xhap2_15JUL2025.csv")

# Calculate mean prenatal exposures

# Limit HAP data to prenatal measurements only
hap3 = hap2[hap2$visit=="BL" | hap2$visit=="P1" | hap2$visit=="P2",]

# Create a wide HAP dataset for prenatal PM
lpg = aggregate (lpg ~ hhid, data = hap3, FUN=max)
irc = aggregate (irc ~ hhid, data = hap3, FUN=unique)

blhap3 = subset(hap3, select=c(      hhid,
                                     echildpm_date,
                                     echildpm,
                                     echildco))[hap3$visit=="BL",]
p1hap3 = subset(hap3, select=c(      hhid,
                                     echildpm_date,
                                     echildpm,
                                     echildco))[hap3$visit=="P1",]
p2hap3 = subset(hap3, select=c(      hhid,
                                     echildpm_date,
                                     echildpm,
                                     echildco))[hap3$visit=="P2",]

names(blhap3)=c("hhid","bldate","blpm","blco")
names(p1hap3)=c("hhid","p1date","p1pm","p1co")
names(p2hap3)=c("hhid","p2date","p2pm","p2co")

xhap3 = merge(lpg, irc, by="hhid",all=T)
xhap3 = merge(xhap3, blhap3, by="hhid",all=T)
xhap3 = merge(xhap3, p1hap3, by="hhid",all=T)
xhap3 = merge(xhap3, p2hap3, by="hhid",all=T)

# Calculate mean of differences for BL - mean (P1, P2)
# Limit data to complete cases only
# D=BL-mean(P1, P2) for complete {BL, P1, P2} sets

y1hap3 = xhap3[,-c(4,6,7,9,10,12)]
y2hap3 = xhap3[,-c(4,5,7,8,10,11)]
y1hap3 = y1hap3[complete.cases(y1hap3),]
y2hap3 = y2hap3[complete.cases(y2hap3),]
z1hap3 = y1hap3[y1hap3$lpg==0,]
y1hap3 = y1hap3[y1hap3$lpg==1,]

y1hap3=as.data.frame(y1hap3 %>%
       group_by(hhid) %>%
       mutate(diffpm=blpm - mean(c(p1pm,p2pm))) %>%
       ungroup())
```

© 2025 McCracken JP et al. *JAMA Network Open*.

```
y2hap3=as.data.frame(y2hap3 %>%
        group_by(hhid) %>%
        mutate(diffco=blco - mean(c(p1co,p2co))) %>%
        ungroup())

# Calculate D specific to each country
diffpm = aggregate(diffpm~irc,data=y1hap3,FUN=mean)
diffco = aggregate(diffco~irc,data=y2hap3,FUN=mean)

diffpm_lpg_guatemala = diffpm$diff[1]
diffpm_lpg_india = diffpm$diff[2]
diffpm_lpg_peru = diffpm$diff[3]
diffpm_lpg_rwanda = diffpm$diff[4]

diffco_lpg_guatemala = diffco$diff[1]
diffco_lpg_india = diffco$diff[2]
diffco_lpg_peru = diffco$diff[3]
diffco_lpg_rwanda = diffco$diff[4]


xhap3$meanp1p2pm = rowMeans(xhap3[,c(8,11)],na.rm=T)
xhap3$meanp1p2co = rowMeans(xhap3[,c(9,12)],na.rm=T)

# Compute BL imputed as D + mean(P1, P2) for LPG=1 (intervention)
 participants) for each countryyhap3 = xhap3[xhap3$irc=="Guatemala",]
yhap3$corr_d_blpm =   ifelse (is.na(yhap3$blpm)==T & yhap3$lpg==1,
                      diffpm_lpg_guatemala+yhap3$meanp1p2pm, yhap3$blpm)
yhap3$corr_d_blco =   ifelse (is.na(yhap3$blco)==T & yhap3$lpg==1,
                      diffco_lpg_guatemala+yhap3$meanp1p2co, yhap3$blco)
whap3 = yhap3

yhap3 = xhap3[xhap3$irc=="India",]
yhap3$corr_d_blpm =   ifelse (is.na(yhap3$blpm)==T & yhap3$lpg==1 ,
                      diffpm_lpg_india+yhap3$meanp1p2pm, yhap3$blpm)
yhap3$corr_d_blco =   ifelse (is.na(yhap3$blco)==T & yhap3$lpg==1 ,
                      diffco_lpg_india+yhap3$meanp1p2co, yhap3$blco)
whap3 = rbind(whap3, yhap3)

yhap3 = xhap3[xhap3$irc=="Peru",]
yhap3$corr_d_blpm =   ifelse (is.na(yhap3$blpm)==T & yhap3$lpg==1 ,
                      diffpm_lpg_peru+yhap3$meanp1p2pm, yhap3$blpm)
yhap3$corr_d_blco =   ifelse (is.na(yhap3$blco)==T & yhap3$lpg==1 ,
                      diffco_lpg_peru+yhap3$meanp1p2co, yhap3$blco)
whap3 = rbind(whap3, yhap3)

yhap3 = xhap3[xhap3$irc=="Rwanda",]
yhap3$corr_d_blpm =   ifelse (is.na(yhap3$blpm)==T & yhap3$lpg==1 ,
                      diffpm_lpg_rwanda+yhap3$meanp1p2pm, yhap3$blpm)
yhap3$corr_d_blco =   ifelse (is.na(yhap3$blco)==T & yhap3$lpg==1 ,
                      diffco_lpg_rwanda+yhap3$meanp1p2co, yhap3$blco)
whap3 = rbind(whap3, yhap3)

whap3$dprenatalpm =   rowMeans(whap3[,c(15, 8, 11)], na.rm=T)
whap3$uprenatalpm =   rowMeans(whap3[,c(5,8,11)], na.rm=T)
whap3$dprenatalpm =   ifelse (whap3$lpg==0, whap3$uprenatalpm,
                      whap3$dprenatalpm)
```

© 2025 McCracken JP et al. *JAMA Network Open*.

```
whap3$dprenatalco =    rowMeans(whap3[,c(16, 9, 12)], na.rm=T)
whap3$uprenatalco =    rowMeans(whap3[,c(6,9,12)], na.rm=T)
whap3$dprenatalco =    ifelse (whap3$lpg==0, whap3$uprenatalco,
                       whap3$dprenatalco)


# Limit HAP data set to difference-based prenatal exposuress
xhap6 = subset(whap3, select=c(hhid, dprenatalpm, dprenatalco))

# Add prenatal exposures into ER-3 data
xer3 = merge (xer3, xhap6, by="hhid")

# Reorder ER-3 data by id, quarter
xer3 = xer3 [order(xer3$hhid, xer3$start),]

## Calculate postnatal PM

# Limit data to post-natal measurements
hap5 = hap2[hap2$visit=="B1" | hap2$visit=="B2" | hap2$visit=="B4",]

# Calculate mean postnatal PM, this function averages across existing values

hap6 = aggregate(echildpm ~ hhid, data = hap5, FUN=mean)
names(hap6)[2]="postnatalpm"

## Option 1
## For-loops to calculate mean postnatal PM
## if quarter = 1, calculate PM from B1 visit
## if quarter = 2, calculate mean PM from B1 and B2 visits
## if quarter = 3, calculate mean PM from B1 and B2 visits
## if quarter = 4, calculate mean PM from B1, B2 and B4 visits
## Algorithm calculates mean of available non-NA values

# Unique set of IDs
uhhid = unique (xer3$hhid)

# NULL data.frame
xer4 = NULL

for (i in 1:length(uhhid))
        {
        # Subset post-natal HAP data for the ith infant
        uhaper3 = hap5 [ hap5$hhid == uhhid[i],]

        # Subset ER-3 data for the ith infant
        uxer3 = xer3 [ xer3$hhid == uhhid[i],]

        # Fill in post-natal exposures using above rules

        for (j in 1:dim(uxer3)[1])
                {
                if (uxer3$quarter[j]==1)
                uxer3$postnatalpm[j]=mean(uhaper3$echildpm[uhaper3$visit=="B1"
                ])
```

© 2025 McCracken JP et al. *JAMA Network Open*.

```
            if (uxer3$quarter[j]==2)
            uxer3$postnatalpm[j]=mean(c(uhaper3$echildpm[uhaper3$visit=="B1
            "], uhaper3$echildpm[uhaper3$visit=="B2"]), na.rm=T)

             if (uxer3$quarter[j]==3)
             uxer3$postnatalpm[j]=mean(c(uhaper3$echildpm[uhaper3$visit=="B
             1"], uhaper3$echildpm[uhaper3$visit=="B2"]), na.rm=T)

             if (uxer3$quarter[j]==4)
             uxer3$postnatalpm[j]=mean(c(uhaper3$echildpm[uhaper3$visit=="B
             1"], uhaper3$echildpm[uhaper3$visit=="B2"],
             uhaper3$echildpm[uhaper3$visit=="B4"]), na.rm=T)

             if (uxer3$quarter[j]==1)
             uxer3$postnatalco[j]=mean(uhaper3$echildco[uhaper3$visit=="B1"
             ])

             if (uxer3$quarter[j]==2)
             uxer3$postnatalco[j]=mean(c(uhaper3$echildco[uhaper3$visit=="B
             1"], uhaper3$echildco[uhaper3$visit=="B2"]), na.rm=T)

             if (uxer3$quarter[j]==3)
             uxer3$postnatalco[j]=mean(c(uhaper3$echildco[uhaper3$visit=="B
             1"], uhaper3$echildco[uhaper3$visit=="B2"]), na.rm=T)
             if (uxer3$quarter[j]==4)
             uxer3$postnatalco[j]=mean(c(uhaper3$echildco[uhaper3$visit=="B
             1"],
             uhaper3$echildco[uhaper3$visit=="B2"],
             uhaper3$echildco[uhaper3$visit=="B4"]), na.rm=T)

            }

        xer4 = rbind(xer4, uxer3)
        }

# Calculate quartiles for prenatal and post-natal PM and CO

xer4$dprenatalpmcat = cut(    xer4$dprenatalpm,
                              breaks=quantile(xer4$dprenatalpm,
                              c(0,0.25,0.5, 0.75,1), na.rm=T),
                              include.lowest=T)

xer4$dprenatalcocat = cut(    xer4$dprenatalco,
                              breaks=quantile(xer4$dprenatalco,
                              c(0,0.25,0.5, 0.75,1), na.rm=T),
                              include.lowest=T)


xer4$postnatalpmcat = cut(    xer4$postnatalpm,
                              breaks=quantile(xer4$postnatalpm,
                              c(0,0.25,0.5, 0.75,1), na.rm=T),
                              include.lowest=T)

xer4$postnatalcocat = cut(    xer4$postnatalco,
                              breaks=quantile(xer4$postnatalco,
                              c(0,0.25,0.5, 0.75,1), na.rm=T),
                              include.lowest=T)
```

© 2025 McCracken JP et al. *JAMA Network Open*.

```
# Repopulate confounders
# m10_sleep            number of people sleeping in house
# c30_sex              sex, 1 male 2 female (original M male, F female)
# m10_educ_R           maternal education
# diet_diverse         diet diversity
# fies                 food security score
# WeightZscore         birthweight-for-gestational age Z-score
# Birthwt_by_protocol Birthweight (g)

conf  = aggregate (m10_sleep ~ hhid, data=er3, FUN=unique)
conf2 = aggregate (c30_sex ~ hhid, data=er3, FUN=unique)
conf = merge(conf, conf2, by="hhid", all=T)
conf2 = aggregate (m10_educ_R ~ hhid, data=er3, FUN=unique)
conf = merge(conf, conf2, by="hhid", all=T)
conf2 = aggregate (diet_diverse ~ hhid, data=er3, FUN=unique)
conf = merge(conf, conf2, by="hhid", all=T)
conf2 = aggregate (fies ~ hhid, data=er3, FUN=unique)
conf = merge(conf, conf2, by="hhid", all=T)
conf2 = aggregate (WeightZScore ~ hhid, data=er3, FUN=unique)
conf = merge(conf, conf2, by="hhid", all=T)
conf2 = aggregate (Birthwt_by_protocol ~ hhid, data=er3, FUN=unique)
conf = merge(conf, conf2, by="hhid", all=T)

# Add SES variable
conf = merge(conf, ses, by="hhid",all=T)

# SES quartiles for categorization
sesquart = quantile(conf$ses, c(0,0.25,0.5,0.75,1) )

# Rename sex as ma0fe1 (male 0, female 1)
names(conf)[3]="ma0fe1"
# c30_sex was provided earlier by DMC as M/F, now DMC provided it as 1=M, 2=F
#conf$ma0fe1 = ifelse(conf$ma0fe1=="Male", 0, 1)
conf$ma0fe1 = conf$ma0fe1-1

# Rename birthweight variables
names(conf)[7]="bwz"
names(conf)[8]="birthweight"

# Merge ER-3 data with confounders
xer5 = merge (xer4, conf, by="hhid")

# Rename levels of maternal education to 1, 2 and 3, and rename as momedu
xer5$momedu = factor(as.numeric(factor(xer5$m10_educ_R)))

# Make a new variable crowding named sleep10 if 10+ people sleep in the house
xer5$sleep10 = ifelse(xer5$m10_sleep>=10,1,0)

# Create crowding by categorizing number of people who sleep in the house
 into quartiles
conf2 = aggregate(m10_sleep ~ hhid, data=er3, FUN=unique)
xer5$crowding = cut(   xer5$m10_sleep,
                       breaks = quantile(conf2$m10_sleep,0:4/4),
                       include.lowest=T, right=T)

# Make categories of SES using values in sesquart
```

© 2025 McCracken JP et al. *JAMA Network Open*.

```
xer5$sescat = cut(xer5$ses, breaks=sesquart, include.lowest=T)

# Limit variables to create a final dataset
xer6 = subset(xer5, select=c(
                                       hhid,
                                       irc,
                                       quarter,
                                       start,
                                       stop,
                                       pneumonia,
                                       whosevere,
                                       dprenatalpm,
                                       dprenatalpmcat,
                                       dprenatalco,
                                       dprenatalcocat,
                                       postnatalpm,
                                       postnatalpmcat,
                                       postnatalco,
                                       postnatalcocat,
                                       m10_sleep,
                                       sleep10,
                                       crowding,
                                       ma0fe1,
                                       winter,
                                       COVID,
                                       bwz,
                                       birthweight,
                                       ses,
                                       sescat,
                                       pcv))

# Rename variables for consistency in names and case
names(xer6)[16]="sleephouse"
names(xer6)[21]="covid"


## Option 2
## For-loops to calculate mean postnatal PM, use the denomination pm_opt
## if quarter = 1, calculate mean PM from P2 and B1 visits; if missing use B2
## visit; if missing use B4 visit
## if quarter = 2, calculate mean PM from P2, B1 and B2 visits
## if quarter = 3, calculate mean PM from P2, B1 and B2 visits
## if quarter = 4, calculate mean PM from P2, B1, B2 and B4 visits
## Algorithm calculates mean of available non-NA values

# Limit data to P2 and post-natal measurements
hap7 = hap2[hap2$visit=="P2" | hap2$visit=="B1" | hap2$visit=="B2" |
 hap2$visit=="B4",]

uhhid=unique(xer6$hhid)
xer7 = NULL

for (i in 1:length(uhhid))
        {

        # Subset post-natal HAP data for child i
        uhaper3 = hap7 [ hap7$hhid == uhhid[i],]
```

© 2025 McCracken JP et al. *JAMA Network Open*.

```
# Subset ER-3 data for child i
uxer3 = xer6 [ xer6$hhid == uhhid[i],]

# Fill in post-natal PM data with rules above
for (j in 1:dim(uxer3)[1])
        {

        if (uxer3$quarter[j]==1)
        uxer3$postnatalpm_opt[j]=mean(c(uhaper3$echildpm[uhaper3$visit=
        ="B1"], uhaper3$echildpm[uhaper3$visit=="P2"]), na.rm=T)

        if (uxer3$quarter[j]==2)
        uxer3$postnatalpm_opt[j]=mean(c(uhaper3$echildpm[uhaper3$visit=
        ="B1"], uhaper3$echildpm[uhaper3$visit=="B2"],
        uhaper3$echildpm[uhaper3$visit=="P2"]),na.rm=T)

        if (uxer3$quarter[j]==3)
        uxer3$postnatalpm_opt[j]=mean(c(uhaper3$echildpm[uhaper3$visit
        =="B1"], uhaper3$echildpm[uhaper3$visit=="B2"],
        uhaper3$echildpm[uhaper3$visit=="P2"]),na.rm=T)
        if (uxer3$quarter[j]==4)
        uxer3$postnatalpm_opt[j]=mean(c(uhaper3$echildpm[uhaper3$visit=
        ="B1"],uhaper3$echildpm[uhaper3$visit=="B2"],
        uhaper3$echildpm[uhaper3$visit=="P2"],
        uhaper3$echildpm[uhaper3$visit=="B4"]), na.rm=T)

        if (uxer3$quarter[j]==1)
        uxer3$postnatalco_opt[j]=mean(c(uhaper3$echildco[uhaper3$visit=
        ="B1"],uhaper3$echildco[uhaper3$visit=="P2"]),na.rm=T)

        if (uxer3$quarter[j]==2)
        uxer3$postnatalco_opt[j]=mean(c(uhaper3$echildco[uhaper3$visit=
        ="B1"], uhaper3$echildco[uhaper3$visit=="B2"],
        uhaper3$echildco[uhaper3$visit=="P2"]), na.rm=T)

        if (uxer3$quarter[j]==3)
        uxer3$postnatalco_opt[j]=mean(c(uhaper3$echildco[uhaper3$visit
        =="B1"], uhaper3$echildco[uhaper3$visit=="B2"],
        uhaper3$echildco[uhaper3$visit=="P2"]), na.rm=T)

        if (uxer3$quarter[j]==4)
         uxer3$postnatalco_opt[j]=mean(c(uhaper3$echildco[uhaper3$visit
         =="B1"],uhaper3$echildco[uhaper3$visit=="B2"],uhaper3$echildco
         [uhaper3$visit=="P2"], uhaper3$echildco[uhaper3$visit=="B4"]),
         na.rm=T)

        if (uxer3$quarter[j]==1 & is.na(uxer3$postnatalpm_opt[j])==T )
         uxer3$postnatalpm_opt[j]=mean(uhaper3$echildpm[uhaper3$visit==
         "B2"])

        if (uxer3$quarter[j]==1 & is.na(uxer3$postnatalpm_opt[j])==T )
         uxer3$postnatalpm_opt[j]=mean(uhaper3$echildpm[uhaper3$visit==
         "B4"])
```

© 2025 McCracken JP et al. *JAMA Network Open*.

```
                if (uxer3$quarter[j]==1 & is.na(uxer3$postnatalco_opt[j])==T )
                 uxer3$postnatalco_opt[j]=mean(uhaper3$echildco[uhaper3$visit==
                 "B2"])

                if (uxer3$quarter[j]==1 & is.na(uxer3$postnatalpm_opt[j])==T )
                 uxer3$postnatalco_opt[j]=mean(uhaper3$echildco[uhaper3$visit==
                 "B4"])

                }

                xer7 = rbind(xer7, uxer3)
        }

# Add method 2 postnatal exposures to xer6
xer6$postnatalpm_opt=xer7$postnatalpm_opt
xer6$postnatalco_opt=xer7$postnatalco_opt

# Calculate quartiles for method 2


xer6$postnatalpmcat_opt = cut(       xer6$postnatalpm_opt,
                                     breaks=quantile(xer6$postnatalpm_opt,
                                     c(0,0.25,0.5, 0.75,1), na.rm=T),
                                     include.lowest=T)

xer6$postnatalcocat_opt = cut(       xer6$postnatalco_opt,
                                     breaks=quantile(xer6$postnatalco_opt,
                                     c(0,0.25,0.5, 0.75,1), na.rm=T),
                                     include.lowest=T)

## Reorder xer6

# ER-3 dataset consists of the following:
#
# hhid                   participant ID
# irc                    IRC Guatemala, India, Peru, Rwanda
# quarter                quarters 1, 2, 3, 4
# start                  quarter start date
# stop          `        quarter stop date
# pneumonia              severe infant pneumonia episode 1 yes, 0 no
# whosevere              severe IMCI infant pneumonia episode 1 yes, 0 no
# dprenatalpm            difference-based unweighted prenatal PM
# dprenatalpmcat         difference-based unweighted prenatal PM quartiles
# dprenatalco            difference-based unweighted prenatal CO
# dprenatalcocat         difference-based unweighted prenatal CO quartiles
# postnatalpm            post-natal PM (method 1)
# postnatalpmcat         post-natal PM (method 1) quartiles
# postnatalco            Post-natal CO (method 1)
# postnatalcocat         post-natal CO (method 1) quartiles
# postnatalpm_opt        post-natal PM (method 2)
# postnatalpmcat_opt     post-natal PM (method 2) quartiles
# postnatalco_opt        Post-natal CO (method 2)
# postnatalcocat_opt     post-natal CO (method 2) quartiles
# sleephouse             number of people who sleep in house
# sleep10                10+ people sleep in house
# crowding               number of people who sleep in house in quartiles
# ma0fe1                 sex male 0, female 1
```

© 2025 McCracken JP et al. *JAMA Network Open*.

```
# winter               winter season 1 yes, 0 no
# covid                COVID-19 pandemic time period 1 yes, 0 no
# bwz                  birthweight-for-gestational-age Z-score
#                      (INTERGROWTH 21st)
# birthweight          birthweight in g
# ses                  socioeconomic index
# sescat               socioeconomic index as quartiles
# pcv                  PCV vaccination 1 1+ doses, 0 none


xer6 = subset(xer6, select=c(
                              hhid,
                              irc,
                              quarter,
                              start,
                              stop,
                              pneumonia,
                              whosevere,
                              dprenatalpm,
                              dprenatalpmcat,
                              dprenatalco,
                              dprenatalcocat,
                              postnatalpm,
                              postnatalpmcat,
                              postnatalco,
                              postnatalcocat,
                              postnatalpm_opt,
                              postnatalpmcat_opt,
                              postnatalco_opt,
                              postnatalcocat_opt,
                              sleephouse,
                              sleep10,
                              crowding,
                              ma0fe1,
                              winter,
                              covid,
                              bwz,
                              birthweight,
                              ses,
                              sescat,
                              pcv))


# We made a group decision to use Option 2 to compute post-natal exposures
# Subset data to option 2

er = subset(xer6, select=c(
                              hhid,
                              irc,
                              quarter,
                              start,
                              stop,
                              pneumonia,
                              whosevere,
                              dprenatalpm,
                              dprenatalpmcat,
                              dprenatalco,
```

© 2025 McCracken JP et al. *JAMA Network Open*.

```
                                          dprenatalcocat,
                                          postnatalpm_opt,
                                          postnatalpmcat_opt,
                                          postnatalco_opt,
                                          postnatalcocat_opt,
                                          sleephouse,
                                          sleep10,
                                          crowding,
                                          ma0fe1,
                                          winter,
                                          covid,
                                          bwz,
                                          birthweight,
                                          ses,
                                          sescat,
                                          pcv))


names(er)[12]="postnatalpm"
names(er)[13]="postnatalpmcat"
names(er)[14]="postnatalco"
names(er)[15]="postnatalcocat"

# Final ER-3 dataset consists of the following:
#
# hhid                  participant ID
# irc                   IRC Guatemala, India, Peru, Rwanda
# quarter               quarters 1, 2, 3, 4
# start                 quarter start date
# stop         `        quarter stop date
# pneumonia             severe infant pneumonia episode 1 yes, 0 no
# whosevere             severe IMCI infant pneumonia episode 1 yes, 0 no
# dprenatalpm           difference-based unweighted prenatal PM
# dprenatalpmcat        difference-based unweighted prenatal PM quartiles
# dprenatalco           difference-based unweighted prenatal CO
# dprenatalcocat        difference-based unweighted prenatal CO quartiles
# postnatalpm           post-natal PM (method 2)
# postnatalpmcat        post-natal PM (method 2) quartiles
# postnatalco           Post-natal CO (method 2)
# postnatalcocat        post-natal CO (method 2) quartiles
# sleephouse            number of people who sleep in house
# sleep10               10+ people sleep in house
# crowding              number of people who sleep in house in quartiles
# ma0fe1                sex male 0, female 1
# winter                winter season 1 yes, 0 no
# covid                 COVID-19 pandemic time period 1 yes, 0 no
# bwz                   birthweight-for-gestational-age Z-score
#                        (INTERGROWTH 21st)
# birthweight           birthweight in g
# ses                   socioeconomic index
# sescat                socioeconomic index as quartiles
# pcv                   PCV vaccination 1 1+ doses, 0 none


# Output database as CSV
# write.csv(er,"er_17JUL2025.csv",row.names=F)
```

© 2025 McCracken JP et al. *JAMA Network Open*.

```
# Calculate the difference in IQRs for PM2.5 and CO exposures
predpmiqr = diff(quantile(er$dprenatalpm,c(0.25,0.75),na.rm=T))
postpmiqr = diff(quantile(er$postnatalpm,c(0.25,0.75),na.rm=T))

predcoiqr = diff(quantile(er$dprenatalco,c(0.25,0.75),na.rm=T))
postcoiqr = diff(quantile(er$postnatalco,c(0.25,0.75),na.rm=T))

# Show IQRs
quantile(er$dprenatalpm,c(0.25,0.75),na.rm=T)
quantile(er$postnatalpm,c(0.25,0.75),na.rm=T)
quantile(er$dprenatalco,c(0.25,0.75),na.rm=T)
quantile(er$postnatalco,c(0.25,0.75),na.rm=T)

## Regression models
#
# use pneumonia for severe infant pneumonia
# use whosevere for severe IMCI infant pneumonia
# use dprenatalpm for difference-derived prenatal PM2.5
# use dprenatalco for difference-derived prenatal CO
# the _iqr values calculated above represent the scalars for PM or CO
# values, i.e., scaling the PM2.5/CO exposures to the IQR difference

## Main model for severe infant pneumonia and PM2.5 exposures
## Use GEE from library(gee)
##
model = gee (pneumonia ~
                      factor(quarter) +
                      I(dprenatalpm/predpmiqr) +
                      I(postnatalpm/postpmiqr) +
                      crowding +
                      ma0fe1 +
                      birthweight +
                      winter +
                      covid +
                      sescat +
                      pcv +
                      irc,
                      data= er,
                      id=hhid,
                      family=binomial(link="log"),
                      corstr="exchangeable")

# Calculate RR and 95% CI
x=round(summary(model)$coef,4)
round(x,4)
y=data.frame(  RR=exp(x[,1]),
               LB95=exp(x[,1]- qnorm(0.975)*x[,4]),
               UB95=exp(x[,1]+ qnorm(0.975)*x[,4]))
round(y,2)
```

```
> model = gee (pneumonia ~
+                             factor(quarter) +
+                             I(dprenatalpm/predpmiqr) +
+                             I(postnatalpm/postpmiqr) +
+                             crowding +
+                             ma0fe1 +
```

© 2025 McCracken JP et al. *JAMA Network Open*.

```
+                             birthweight +
+                             winter +
+                             covid +
+                             sescat +
+                             pcv +
+                             irc,
+                             data= er,
+                             id=hhid,
+                             family=binomial(link="log"),
+                             corstr="exchangeable")

Beginning Cgee S-function, @(#) geeformula.q 4.13 98/01/27
running glm to get initial regression estimate
             (Intercept)         factor(quarter)2         factor(quarter)3
           -1.0963685881            -1.0038459872            -1.1404020369
        factor(quarter)4 I(dprenatalpm/predpmiqr) I(postnatalpm/postpmiqr)
           -0.9272148563             0.0329884414            -0.0280438339
           crowding(3,4]            crowding(4,5]           crowding(5,18]
            0.0261714561             0.3218757899             0.1304036690
                  ma0fe1              birthweight                   winter
           -0.0925809159            -0.0006644139             0.5814462376
                   covid      sescat(0.198,0.353]      sescat(0.353,0.529]
           -0.1482814564            -0.1337043679            -0.5170052175
         sescat(0.529,1]                      pcv                 ircIndia
            0.1667964615            -0.5386172116            -2.7030167317
                 ircPeru                ircRwanda
           -1.3254581197             0.6180722223

> # Calculate RR and 95% CI
> x=round(summary(model)$coef,4)
> round(x,4)
                         Estimate Naive S.E. Naive z Robust S.E. Robust z
(Intercept)               -1.0393     0.5575 -1.8642      0.7251  -1.4334
factor(quarter)2          -1.0003     0.1994 -5.0164      0.2209  -4.5282
factor(quarter)3          -1.1398     0.2170 -5.2519      0.2572  -4.4322
factor(quarter)4          -0.9330     0.2134 -4.3731      0.2580  -3.6159
I(dprenatalpm/predpmiqr)   0.0312     0.0584  0.5336      0.0472   0.6603
I(postnatalpm/postpmiqr)  -0.0309     0.0565 -0.5471      0.0577  -0.5365
crowding(3,4]              0.0386     0.2001  0.1930      0.2227   0.1733
crowding(4,5]              0.3218     0.2185  1.4726      0.2365   1.3604
crowding(5,18]             0.1502     0.2047  0.7338      0.2282   0.6580
ma0fe1                    -0.0993     0.1459 -0.6803      0.1647  -0.6029
birthweight               -0.0007     0.0002 -3.8619      0.0002  -2.9543
winter                     0.5782     0.2136  2.7067      0.2441   2.3693
covid                     -0.1224     0.1620 -0.7558      0.2071  -0.5912
sescat(0.198,0.353]       -0.1438     0.1943 -0.7401      0.2151  -0.6686
sescat(0.353,0.529]       -0.5429     0.2775 -1.9564      0.3315  -1.6378
sescat(0.529,1]            0.1508     0.3784  0.3985      0.3598   0.4191
pcv                       -0.5580     0.1874 -2.9782      0.2044  -2.7305
ircIndia                  -2.6982     0.4656 -5.7955      0.4506  -5.9881
ircPeru                   -1.3187     0.3167 -4.1643      0.3273  -4.0294
ircRwanda                  0.5997     0.2498  2.4007      0.3082   1.9456

> y=data.frame(       RR=exp(x[,1]),
+                     LB95=exp(x[,1]- qnorm(0.975)*x[,4]),
+                     UB95=exp(x[,1]+ qnorm(0.975)*x[,4]))
> round(y,2)
```

© 2025 McCracken JP et al. *JAMA Network Open*.

```
                             RR LB95 UB95
(Intercept)                0.35 0.09 1.47
factor(quarter)2           0.37 0.24 0.57
factor(quarter)3           0.32 0.19 0.53
factor(quarter)4           0.39 0.24 0.65
I(dprenatalpm/predpmiqr)   1.03 0.94 1.13
I(postnatalpm/postpmiqr)   0.97 0.87 1.09
crowding(3,4]              1.04 0.67 1.61
crowding(4,5]              1.38 0.87 2.19
crowding(5,18]             1.16 0.74 1.82
ma0fe1                     0.91 0.66 1.25
birthweight                1.00 1.00 1.00
winter                     1.78 1.10 2.88
covid                      0.88 0.59 1.33
sescat(0.198,0.353]        0.87 0.57 1.32
sescat(0.353,0.529]        0.58 0.30 1.11
sescat(0.529,1]            1.16 0.57 2.35
pcv                        0.57 0.38 0.85
ircIndia                   0.07 0.03 0.16
ircPeru                    0.27 0.14 0.51
ircRwanda                  1.82 1.00 3.33
```

```
## Calc unadjusted RRs for PM2.5 exposures
# Prenatal PM2.5
model = gee (pneumonia ~
                       factor(quarter) +
                       I(dprenatalpm/predpmiqr),
                       data= er,
                       id=hhid,
                       family=binomial(link="log"),
                       corstr="exchangeable")


# Calculate RR and 95% CI
x=round(summary(model)$coef,4)
round(x,4)
y=data.frame(  RR=exp(x[,1]),
               LB95=exp(x[,1]- qnorm(0.975)*x[,4]),
               UB95=exp(x[,1]+ qnorm(0.975)*x[,4]))

round(y,2)
```

```
> model = gee (pneumonia ~
+                            factor(quarter) +
+                            I(dprenatalpm/predpmiqr),
+                            data= er,
+                            id=hhid,
+                            family=binomial(link="log"),
+                            corstr="exchangeable")

Beginning Cgee S-function, @(#) geeformula.q 4.13 98/01/27
running glm to get initial regression estimate
             (Intercept)          factor(quarter)2          factor(quarter)3
```

© 2025 McCracken JP et al. *JAMA Network Open*.

```
             -3.6683658                 -1.1696559                 -1.3254968
        factor(quarter)4 I(dprenatalpm/predpmiqr)
             -1.0536784                  0.1040626

> # Calculate RR and 95% CI
> x=round(summary(model)$coef,4)
> round(x,4)
                         Estimate Naive S.E.  Naive z Robust S.E. Robust z
(Intercept)               -3.6658     0.1283 -28.5809      0.1224 -29.9433
factor(quarter)2          -1.1573     0.2120  -5.4590      0.2110  -5.4850
factor(quarter)3          -1.3061     0.2254  -5.7947      0.2294  -5.6935
factor(quarter)4          -1.0360     0.2030  -5.1024      0.2039  -5.0820
I(dprenatalpm/predpmiqr)   0.1023     0.0515   1.9859      0.0415   2.4656
> y=data.frame(       RR=exp(x[,1]),
+                     LB95=exp(x[,1]- qnorm(0.975)*x[,4]),
+                     UB95=exp(x[,1]+ qnorm(0.975)*x[,4]))
>
> round(y,2)
                           RR LB95 UB95
(Intercept)              0.03 0.02 0.03
factor(quarter)2         0.31 0.21 0.48
factor(quarter)3         0.27 0.17 0.42
factor(quarter)4         0.35 0.24 0.53
I(dprenatalpm/predpmiqr) 1.11 1.02 1.20
```

```
# Postnatal PM2.5
model = gee (pneumonia ~
                      factor(quarter) +
                      I(postnatalpm/postpmiqr),
                      data= er,
                      id=hhid,
                      family=binomial(link="log"),
                      corstr="exchangeable")

x=round(summary(model)$coef,4)
round(x,4)
y=data.frame(         RR=exp(x[,1]),
                      LB95=exp(x[,1]- qnorm(0.975)*x[,4]),
                      UB95=exp(x[,1]+ qnorm(0.975)*x[,4]))
round(y,2)
```

```
> model = gee (pneumonia ~
+                              factor(quarter) +
+                              I(postnatalpm/postpmiqr),
+                              data= er,
+                              id=hhid,
+                              family=binomial(link="log"),
+                              corstr="exchangeable")

Beginning Cgee S-function, @(#) geeformula.q 4.13 98/01/27
running glm to get initial regression estimate
             (Intercept)         factor(quarter)2         factor(quarter)3
             -3.60857517              -1.11038326              -1.26810765
        factor(quarter)4 I(postnatalpm/postpmiqr)
```

© 2025 McCracken JP et al. *JAMA Network Open*.

```
              -1.02104813               0.03399509
>
> x=round(summary(model)$coef,4)
> round(x,4)
                            Estimate Naive S.E.  Naive z Robust S.E. Robust z
(Intercept)                  -3.6033     0.1219 -29.5532      0.1161 -31.0292
factor(quarter)2             -1.1024     0.2140  -5.1505      0.2131  -5.1729
factor(quarter)3             -1.2531     0.2274  -5.5100      0.2316  -5.4110
factor(quarter)4             -1.0057     0.2050  -4.9057      0.2056  -4.8924
I(postnatalpm/postpmiqr)      0.0299     0.0447   0.6677      0.0339   0.8819
> y=data.frame(              RR=exp(x[,1]),
+                            LB95=exp(x[,1]- qnorm(0.975)*x[,4]),
+                            UB95=exp(x[,1]+ qnorm(0.975)*x[,4]))

> round(y,2)
                            RR LB95 UB95
(Intercept)               0.03 0.02 0.03
factor(quarter)2          0.33 0.22 0.50
factor(quarter)3          0.29 0.18 0.45
factor(quarter)4          0.37 0.24 0.55
I(postnatalpm/postpmiqr) 1.03 0.96 1.10
```

```
# Main models for CO exposures

model = gee (pneumonia ~
                      factor(quarter) +
                      I(dprenatalco/predcoiqr) +
                      I(postnatalco/postcoiqr) +
                      crowding +
                      ma0fe1 +
                      birthweight +
                      winter +
                      covid +
                      sescat +
                      pcv +
                      irc,
                      data= er,
                      id=hhid,
                      family=binomial(link="log"),
                      corstr="exchangeable")


# Calculate RR and 95% CI
x=round(summary(model)$coef,4)
round(x,4)
y=data.frame(  RR=exp(x[,1]),
               LB95=exp(x[,1]- qnorm(0.975)*x[,4]),
               UB95=exp(x[,1]+ qnorm(0.975)*x[,4]))
round(y,2)
```

```
> model = gee (pneumonia ~
+                            factor(quarter) +
+                            I(dprenatalco/predcoiqr) +
+                            I(postnatalco/postcoiqr) +
```

© 2025 McCracken JP et al. *JAMA Network Open*.

```
+                              crowding +
+                              ma0fe1 +
+                              birthweight +
+                              winter +
+                              covid +
+                              sescat +
+                              pcv +
+                              irc,
+                              data= er,
+                              id=hhid,
+                              family=binomial(link="log"),
+                              corstr="exchangeable")

Beginning Cgee S-function, @(#) geeformula.q 4.13 98/01/27
running glm to get initial regression estimate
            (Intercept)         factor(quarter)2         factor(quarter)3
          -0.7932838432            -1.0627156804            -1.1596408328
       factor(quarter)4 I(dprenatalco/predcoiqr) I(postnatalco/postcoiqr)
          -0.9471897948             0.0245033657            -0.0852485228
          crowding(3,4]            crowding(4,5]           crowding(5,18]
          -0.0046695577             0.2790847117             0.0724853986
                 ma0fe1              birthweight                   winter
          -0.1075785652            -0.0007280965             0.4978685561
                  covid      sescat(0.198,0.353]      sescat(0.353,0.529]
          -0.1488792210            -0.1230334925            -0.5269881045
         sescat(0.529,1]                      pcv                 ircIndia
           0.1609908978            -0.4877426665            -2.6873551550
                ircPeru                ircRwanda
          -1.3466750978             0.5568139798
>
>
> # Calculate RR and 95% CI
> x=round(summary(model)$coef,4)
> round(x,4)
                         Estimate Naive S.E. Naive z Robust S.E. Robust z
(Intercept)               -0.7458     0.5299 -1.4074      0.7117  -1.0480
factor(quarter)2          -1.0595     0.2005 -5.2841      0.2246  -4.7172
factor(quarter)3          -1.1604     0.2155 -5.3852      0.2593  -4.4753
factor(quarter)4          -0.9545     0.2123 -4.4958      0.2604  -3.6654
I(dprenatalco/predcoiqr)   0.0230     0.0606  0.3786      0.0643   0.3571
I(postnatalco/postcoiqr)  -0.0837     0.0669 -1.2523      0.0652  -1.2838
crowding(3,4]              0.0082     0.1976  0.0414      0.2209   0.0370
crowding(4,5]              0.2769     0.2196  1.2608      0.2392   1.1576
crowding(5,18]             0.0905     0.2039  0.4437      0.2286   0.3958
ma0fe1                    -0.1159     0.1450 -0.7991      0.1647  -0.7037
birthweight               -0.0007     0.0002 -4.2790      0.0002  -3.2394
winter                     0.4956     0.2093  2.3678      0.2413   2.0538
covid                     -0.1220     0.1618 -0.7538      0.2076  -0.5877
sescat(0.198,0.353]       -0.1326     0.1930 -0.6872      0.2170  -0.6111
sescat(0.353,0.529]       -0.5496     0.2773 -1.9815      0.3375  -1.6284
sescat(0.529,1]            0.1493     0.3723  0.4011      0.3538   0.4220
pcv                       -0.5057     0.1898 -2.6644      0.2092  -2.4169
ircIndia                  -2.6855     0.4622 -5.8106      0.4503  -5.9635
ircPeru                   -1.3402     0.3401 -3.9404      0.3639  -3.6826
ircRwanda                  0.5386     0.2439  2.2082      0.3015   1.7865
> y=data.frame(       RR=exp(x[,1]),
+                     LB95=exp(x[,1]- qnorm(0.975)*x[,4]),
```

© 2025 McCracken JP et al. *JAMA Network Open*.

```
+                       UB95=exp(x[,1]+ qnorm(0.975)*x[,4]))

> round(y,2)
                             RR LB95 UB95
(Intercept)                0.47 0.12 1.91
factor(quarter)2           0.35 0.22 0.54
factor(quarter)3           0.31 0.19 0.52
factor(quarter)4           0.39 0.23 0.64
I(dprenatalco/predcoiqr)   1.02 0.90 1.16
I(postnatalco/postcoiqr)   0.92 0.81 1.05
crowding(3,4]              1.01 0.65 1.55
crowding(4,5]              1.32 0.83 2.11
crowding(5,18]             1.09 0.70 1.71
ma0fe1                     0.89 0.64 1.23
birthweight                1.00 1.00 1.00
winter                     1.64 1.02 2.63
covid                      0.89 0.59 1.33
sescat(0.198,0.353]        0.88 0.57 1.34
sescat(0.353,0.529]        0.58 0.30 1.12
sescat(0.529,1]            1.16 0.58 2.32
pcv                        0.60 0.40 0.91
ircIndia                   0.07 0.03 0.16
ircPeru                    0.26 0.13 0.53
ircRwanda                  1.71 0.95 3.09
```

```
## Calculate unadjusted RRs for CO exposures
# Prenatal CO
model = gee (pneumonia ~
                       factor(quarter) +
                       I(dprenatalco/predcoiqr),
                       data= er,
                       id=hhid,
                       family=binomial(link="log"),
                       corstr="exchangeable")

# Calculate RR and 95% CI
x=round(summary(model)$coef,4)
round(x,4)
y=data.frame(  RR=exp(x[,1]),
               LB95=exp(x[,1]- qnorm(0.975)*x[,4]),
               UB95=exp(x[,1]+ qnorm(0.975)*x[,4]))
round(y,2)
```

```
> model = gee (pneumonia ~
+                       factor(quarter) +
+                       I(dprenatalco/predcoiqr),
+                       data= er,
+                       id=hhid,
+                       family=binomial(link="log"),
+                       corstr="exchangeable")
Beginning Cgee S-function, @(#) geeformula.q 4.13 98/01/27
running glm to get initial regression estimate
             (Intercept)         factor(quarter)2          factor(quarter)3
             -3.45287303              -1.18278678               -1.33813315
```

© 2025 McCracken JP et al. *JAMA Network Open*.

```
        factor(quarter)4 I(dprenatalco/predcoiqr)
             -1.06738195              -0.06005917
>
> # Calculate RR and 95% DI
> x=round(summary(model)$coef,4)
> round(x,4)
                          Estimate Naive S.E.  Naive z Robust S.E. Robust z
(Intercept)                -3.4525     0.1190 -29.0026      0.1176 -29.3660
factor(quarter)2           -1.1704     0.2123  -5.5124      0.2108  -5.5533
factor(quarter)3           -1.3187     0.2257  -5.8419      0.2291  -5.7550
factor(quarter)4           -1.0496     0.2034  -5.1614      0.2036  -5.1557
I(dprenatalco/predcoiqr)   -0.0605     0.0575  -1.0505      0.0548  -1.1029
> y=data.frame(       RR=exp(x[,1]),
+                     LB95=exp(x[,1]- qnorm(0.975)*x[,4]),
+                     UB95=exp(x[,1]+ qnorm(0.975)*x[,4]))

> round(y,2)
                            RR LB95 UB95
(Intercept)               0.03 0.03 0.04
factor(quarter)2          0.31 0.21 0.47
factor(quarter)3          0.27 0.17 0.42
factor(quarter)4          0.35 0.23 0.52
I(dprenatalco/predcoiqr)  0.94 0.85 1.05
```

```
# Postnatal CO
model = gee (pneumonia ~
                      factor(quarter) +
                      I(postnatalco/postcoiqr),
                      data= er,
                      id=hhid,
                      family=binomial(link="log"),
                      corstr="exchangeable")

x=round(summary(model)$coef,4)
round(x,4)
y=data.frame(  RR=exp(x[,1]),
               LB95=exp(x[,1]- qnorm(0.975)*x[,4]),
               UB95=exp(x[,1]+ qnorm(0.975)*x[,4]))
round(y,2)
```

```
> model = gee (pneumonia ~
+                            factor(quarter) +
+                            I(postnatalco/postcoiqr),
+                            data= er,
+                            id=hhid,
+                            family=binomial(link="log"),
+                            corstr="exchangeable")

Beginning Cgee S-function, @(#) geeformula.q 4.13 98/01/27
running glm to get initial regression estimate
             (Intercept)         factor(quarter)2         factor(quarter)3
              -3.4363025               -1.1609622               -1.2814547
        factor(quarter)4 I(postnatalco/postcoiqr)
              -1.0283233               -0.1181266
```

© 2025 McCracken JP et al. *JAMA Network Open*.

```
>
> x=round(summary(model)$coef,4)
> round(x,4)
                          Estimate Naive S.E.  Naive z Robust S.E. Robust z
(Intercept)                -3.4382     0.1170 -29.3918      0.1128 -30.4909
factor(quarter)2           -1.1527     0.2163  -5.3285      0.2162  -5.3304
factor(quarter)3           -1.2675     0.2266  -5.5948      0.2323  -5.4564
factor(quarter)4           -1.0155     0.2045  -4.9662      0.2072  -4.9021
I(postnatalco/postcoiqr)   -0.1162     0.0671  -1.7303      0.0595  -1.9514
> y=data.frame(       RR=exp(x[,1]),
+                     LB95=exp(x[,1]- qnorm(0.975)*x[,4]),
+                     UB95=exp(x[,1]+ qnorm(0.975)*x[,4]))

> round(y,2)
                            RR LB95 UB95
(Intercept)               0.03 0.03 0.04
factor(quarter)2          0.32 0.21 0.48
factor(quarter)3          0.28 0.18 0.44
factor(quarter)4          0.36 0.24 0.54
I(postnatalco/postcoiqr) 0.89 0.79 1.00
```

```
## Table S2
# Main model for IMCI severe infant pneumonia and PM2.5
model = gee (whosevere ~
                      factor(quarter) +
                      I(dprenatalpm/predpmiqr) +
                      I(postnatalpm/postpmiqr) +
                      crowding +
                      ma0fe1 +
                      birthweight +
                      winter +
                      covid +
                      sescat +
                      pcv +
                      irc,
                      data= er,
                      id=hhid,
                      family=binomial(link="log"),
                      corstr="exchangeable")


# Calculate RR and 95% CI
x=round(summary(model)$coef,4)
round(x,4)
y=data.frame(         RR=exp(x[,1]),
                      LB95=exp(x[,1]- qnorm(0.975)*x[,4]),
                      UB95=exp(x[,1]+ qnorm(0.975)*x[,4]))
round(y,2)
```

```
> model = gee (whosevere ~
+                            factor(quarter) +
+                            I(dprenatalpm/predpmiqr) +
+                            I(postnatalpm/postpmiqr) +
+                            crowding +
+                            ma0fe1 +
```

© 2025 McCracken JP et al. *JAMA Network Open*.

```
+                               birthweight +
+                               winter +
+                               covid +
+                               sescat +
+                               pcv +
+                               irc,
+                               data= er,
+                               id=hhid,
+                               family=binomial(link="log"),
+                               corstr="exchangeable")

Beginning Cgee S-function, @(#) geeformula.q 4.13 98/01/27
running glm to get initial regression estimate
             (Intercept)          factor(quarter)2          factor(quarter)3
            -0.262160842              -1.830206065              -1.792084155
        factor(quarter)4 I(dprenatalpm/predpmiqr) I(postnatalpm/postpmiqr)
            -1.877285338               0.041983712               0.006386833
           crowding(3,4]             crowding(4,5]            crowding(5,18]
             0.175974824               0.345778796               0.117761023
                  ma0fe1               birthweight                    winter
            -0.319843687              -0.000839657               0.482489413
                   covid      sescat(0.198,0.353]      sescat(0.353,0.529]
             0.171831648              -0.059033440              -0.142764123
          sescat(0.529,1]                       pcv                  ircIndia
             0.409956402              -0.232406849              -1.917716634
                 ircPeru                ircRwanda
            -1.360051648               0.503503872
>
>
> # Calculate RR and 95% CI
> x=round(summary(model)$coef,4)
> round(x,4)
                         Estimate Naive S.E. Naive z Robust S.E. Robust z
(Intercept)               -0.2471     0.4491 -0.5501      0.5229  -0.4725
factor(quarter)2          -1.8331     0.2034 -9.0131      0.2116  -8.6632
factor(quarter)3          -1.7965     0.2011 -8.9345      0.2200  -8.1666
factor(quarter)4          -1.8857     0.2107 -8.9507      0.2336  -8.0723
I(dprenatalpm/predpmiqr)   0.0417     0.0467  0.8917      0.0374   1.1139
I(postnatalpm/postpmiqr)   0.0063     0.0423  0.1488      0.0407   0.1547
crowding(3,4]              0.1885     0.1623  1.1615      0.1652   1.1412
crowding(4,5]              0.3427     0.1831  1.8721      0.1837   1.8660
crowding(5,18]             0.1226     0.1726  0.7098      0.1769   0.6926
ma0fe1                    -0.3284     0.1237 -2.6542      0.1281  -2.5640
birthweight               -0.0008     0.0001 -5.9571      0.0002  -5.1811
winter                     0.4761     0.1689  2.8183      0.1777   2.6789
covid                      0.1930     0.1331  1.4495      0.1541   1.2520
sescat(0.198,0.353]       -0.0603     0.1707 -0.3534      0.1798  -0.3355
sescat(0.353,0.529]       -0.1518     0.2190 -0.6930      0.2277  -0.6666
sescat(0.529,1]            0.4084     0.2966  1.3772      0.3102   1.3165
pcv                       -0.2377     0.1682 -1.4134      0.1736  -1.3695
ircIndia                  -1.9118     0.3319 -5.7597      0.3468  -5.5121
ircPeru                   -1.3633     0.2663 -5.1199      0.2674  -5.0986
ircRwanda                  0.4892     0.2082  2.3502      0.2327   2.1022
> y=data.frame(RR=exp(x[,1]),
+              LB95=exp(x[,1]- qnorm(0.975)*x[,4]),
+              UB95=exp(x[,1]+ qnorm(0.975)*x[,4]))
```

© 2025 McCracken JP et al. *JAMA Network Open*.

```
> round(y,2)
                           RR LB95 UB95
(Intercept)              0.78 0.28 2.18
factor(quarter)2         0.16 0.11 0.24
factor(quarter)3         0.17 0.11 0.26
factor(quarter)4         0.15 0.10 0.24
I(dprenatalpm/predpmiqr) 1.04 0.97 1.12
I(postnatalpm/postpmiqr) 1.01 0.93 1.09
crowding(3,4]            1.21 0.87 1.67
crowding(4,5]            1.41 0.98 2.02
crowding(5,18]           1.13 0.80 1.60
ma0fe1                   0.72 0.56 0.93
birthweight              1.00 1.00 1.00
winter                   1.61 1.14 2.28
covid                    1.21 0.90 1.64
sescat(0.198,0.353]      0.94 0.66 1.34
sescat(0.353,0.529]      0.86 0.55 1.34
sescat(0.529,1]          1.50 0.82 2.76
pcv                      0.79 0.56 1.11
ircIndia                 0.15 0.07 0.29
ircPeru                  0.26 0.15 0.43
ircRwanda                1.63 1.03 2.57
```

```
# Unadjusted RRs
# Prenatal PM
model = gee (whosevere ~
                       factor(quarter) +
                       I(dprenatalpm/predpmiqr),
                       data= er,
                       id=hhid,
                       family=binomial(link="log"),
                       corstr="exchangeable")

# Calculate RR and 95% CI
x=round(summary(model)$coef,4)
round(x,4)
y=data.frame(  RR=exp(x[,1]),
               LB95=exp(x[,1]- qnorm(0.975)*x[,4]),
               UB95=exp(x[,1]+ qnorm(0.975)*x[,4]))
round(y,2)
```

```
> model = gee (whosevere ~
+                              factor(quarter) +
+                              I(dprenatalpm/predpmiqr),
+                              data= er,
+                              id=hhid,
+                              family=binomial(link="log"),
+                              corstr="exchangeable")

Beginning Cgee S-function, @(#) geeformula.q 4.13 98/01/27
running glm to get initial regression estimate
             (Intercept)         factor(quarter)2         factor(quarter)3
              -3.0105982               -1.8783610               -1.7997836
        factor(quarter)4 I(dprenatalpm/predpmiqr)
              -1.7583629                0.1129181
>
```

© 2025 McCracken JP et al. *JAMA Network Open*.

```
> # Calculate RR and 95% CI
> x=round(summary(model)$coef,4)
> round(x,4)
                         Estimate Naive S.E.  Naive z Robust S.E. Robust z
(Intercept)               -3.0113     0.0942 -31.9696      0.0882 -34.1441
factor(quarter)2          -1.8738     0.2059  -9.1008      0.2039  -9.1909
factor(quarter)3          -1.7938     0.1991  -9.0082      0.1992  -9.0034
factor(quarter)4          -1.7505     0.1958  -8.9401      0.1950  -8.9764
I(dprenatalpm/predpmiqr)   0.1133     0.0390   2.9094      0.0323   3.5053

> y=data.frame(       RR=exp(x[,1]),
+                     LB95=exp(x[,1]- qnorm(0.975)*x[,4]),
+                     UB95=exp(x[,1]+ qnorm(0.975)*x[,4]))
> round(y,2)
                           RR LB95 UB95
(Intercept)              0.05 0.04 0.06
factor(quarter)2         0.15 0.10 0.23
factor(quarter)3         0.17 0.11 0.25
factor(quarter)4         0.17 0.12 0.25
I(dprenatalpm/predpmiqr) 1.12 1.05 1.19
```

```
# Postnatal PM

model = gee (whosevere ~
                      factor(quarter) +
                      I(postnatalpm/postpmiqr),
                      data= er,
                      id=hhid,
                      family=binomial(link="log"),
                      corstr="exchangeable")

x=round(summary(model)$coef,4)
round(x,4)

y=data.frame(  RR=exp(x[,1]),
               LB95=exp(x[,1]- qnorm(0.975)*x[,4]),
               UB95=exp(x[,1]+ qnorm(0.975)*x[,4]))
round(y,2)
```

```
> model = gee (whosevere ~
+                             factor(quarter) +
+                             I(postnatalpm/postpmiqr),
+                             data= er,
+                             id=hhid,
+                             family=binomial(link="log"),
+                             corstr="exchangeable")

Beginning Cgee S-function, @(#) geeformula.q 4.13 98/01/27
running glm to get initial regression estimate
             (Intercept)         factor(quarter)2         factor(quarter)3
             -2.92085555              -1.86335518              -1.78693014
        factor(quarter)4 I(postnatalpm/postpmiqr)
             -1.77112015               0.05468726
>
> x=round(summary(model)$coef,4)
```

© 2025 McCracken JP et al. *JAMA Network Open*.

```
> round(x,4)
                         Estimate Naive S.E.  Naive z Robust S.E.  Robust z
(Intercept)               -2.9202     0.0856 -34.1049      0.0813 -35.8971
factor(quarter)2          -1.8607     0.2060  -9.0327      0.2045  -9.0991
factor(quarter)3          -1.7825     0.1992  -8.9477      0.1995  -8.9363
factor(quarter)4          -1.7639     0.1959  -9.0036      0.1951  -9.0425
I(postnatalpm/postpmiqr)   0.0541     0.0304   1.7819      0.0262   2.0643

> y=data.frame(       RR=exp(x[,1]),
+                     LB95=exp(x[,1]- qnorm(0.975)*x[,4]),
+                     UB95=exp(x[,1]+ qnorm(0.975)*x[,4]))
> round(y,2)
                           RR LB95 UB95
(Intercept)              0.05 0.05 0.06
factor(quarter)2         0.16 0.10 0.23
factor(quarter)3         0.17 0.11 0.25
factor(quarter)4         0.17 0.12 0.25
I(postnatalpm/postpmiqr) 1.06 1.00 1.11
```

```
# Main model for IMCI severe infant pneumonia and CO
model = gee (whosevere ~
                      factor(quarter) +
                      I(dprenatalco/predcoiqr) +
                      I(postnatalco/postcoiqr) +
                      crowding +
                      ma0fe1 +
                      birthweight +
                      winter +
                      covid +
                      sescat +
                      pcv +
                      irc,
                      data= er,
                      id=hhid,
                      family=binomial(link="log"),
                      corstr="exchangeable")

# Calculate RR and 95% CI
x=round(summary(model)$coef,4)
round(x,4)
y=data.frame(  RR=exp(x[,1]),
               LB95=exp(x[,1]- qnorm(0.975)*x[,4]),
               UB95=exp(x[,1]+ qnorm(0.975)*x[,4]))
round(y,2)
```

```
> model = gee (whosevere ~
+                             factor(quarter) +
+                             I(dprenatalco/predcoiqr) +
+                             I(postnatalco/postcoiqr) +
+                             crowding +
+                             ma0fe1 +
+                             birthweight +
+                             winter +
+                             covid +
```

© 2025 McCracken JP et al. *JAMA Network Open*.

```
+                             sescat +
+                             pcv +
+                             irc,
+                             data= er,
+                             id=hhid,
+                             family=binomial(link="log"),
+                             corstr="exchangeable")

Beginning Cgee S-function, @(#) geeformula.q 4.13 98/01/27
running glm to get initial regression estimate
             (Intercept)         factor(quarter)2          factor(quarter)3
           -0.0074760329            -1.8495544644             -1.8490311127
        factor(quarter)4 I(dprenatalco/predcoiqr) I(postnatalco/postcoiqr)
           -1.9007592467             0.0259091921             -0.0139527082
           crowding(3,4]            crowding(4,5]            crowding(5,18]
            0.1906399742             0.3209324887              0.0942639718
                  ma0fe1              birthweight                   winter
           -0.3266762618            -0.0009195028              0.4892876135
                   covid      sescat(0.198,0.353]      sescat(0.353,0.529]
            0.1749983669            -0.0592995634             -0.1508679075
         sescat(0.529,1]                      pcv                 ircIndia
            0.3140892904            -0.1485584406             -1.8236349488
                 ircPeru               ircRwanda
           -1.4213612720             0.4670416419

> # Calculate RR and 95% CI
> x=round(summary(model)$coef,4)
> round(x,4)
                         Estimate Naive S.E. Naive z Robust S.E. Robust z
(Intercept)                0.0078     0.4223  0.0184      0.4928   0.0158
factor(quarter)2          -1.8531     0.2027 -9.1401      0.2122  -8.7348
factor(quarter)3          -1.8546     0.2037 -9.1034      0.2235  -8.2996
factor(quarter)4          -1.9104     0.2105 -9.0776      0.2346  -8.1432
I(dprenatalco/predcoiqr)   0.0253     0.0457  0.5539      0.0376   0.6731
I(postnatalco/postcoiqr)  -0.0118     0.0392 -0.2999      0.0326  -0.3607
crowding(3,4]              0.2042     0.1596  1.2793      0.1620   1.2606
crowding(4,5]              0.3180     0.1858  1.7111      0.1876   1.6949
crowding(5,18]             0.0975     0.1727  0.5650      0.1773   0.5502
ma0fe1                    -0.3362     0.1233 -2.7261      0.1277  -2.6332
birthweight               -0.0009     0.0001 -6.6869      0.0002  -5.8348
winter                     0.4813     0.1690  2.8487      0.1783   2.6991
covid                      0.1973     0.1334  1.4783      0.1532   1.2874
sescat(0.198,0.353]       -0.0600     0.1683 -0.3564      0.1773  -0.3382
sescat(0.353,0.529]       -0.1578     0.2170 -0.7274      0.2240  -0.7045
sescat(0.529,1]            0.3143     0.2982  1.0540      0.3201   0.9817
pcv                       -0.1519     0.1727 -0.8797      0.1788  -0.8497
ircIndia                  -1.8165     0.3341 -5.4369      0.3590  -5.0601
ircPeru                   -1.4329     0.2876 -4.9830      0.2868  -4.9960
ircRwanda                  0.4495     0.2074  2.1676      0.2325   1.9338

> y=data.frame(       RR=exp(x[,1]),
+                     LB95=exp(x[,1]- qnorm(0.975)*x[,4]),
+                     UB95=exp(x[,1]+ qnorm(0.975)*x[,4]))
> round(y,2)
                           RR LB95 UB95
(Intercept)              1.01 0.38 2.65
factor(quarter)2         0.16 0.10 0.24
```

© 2025 McCracken JP et al. *JAMA Network Open*.

```
factor(quarter)3          0.16 0.10 0.24
factor(quarter)4          0.15 0.09 0.23
I(dprenatalco/predcoiqr)  1.03 0.95 1.10
I(postnatalco/postcoiqr)  0.99 0.93 1.05
crowding(3,4]             1.23 0.89 1.68
crowding(4,5]             1.37 0.95 1.99
crowding(5,18]            1.10 0.78 1.56
ma0fe1                    0.71 0.56 0.92
birthweight               1.00 1.00 1.00
winter                    1.62 1.14 2.30
covid                     1.22 0.90 1.64
sescat(0.198,0.353]       0.94 0.67 1.33
sescat(0.353,0.529]       0.85 0.55 1.32
sescat(0.529,1]           1.37 0.73 2.56
pcv                       0.86 0.61 1.22
ircIndia                  0.16 0.08 0.33
ircPeru                   0.24 0.14 0.42
ircRwanda                 1.57 0.99 2.47
```

```
# Unadjusted RRs
# Prenatal PM
model = gee (whosevere ~
                       factor(quarter) +
                       I(dprenatalco/predcoiqr),
                       data= er,
                       id=hhid,
                       family=binomial(link="log"),
                       corstr="exchangeable")

# Calculate RR and 95% CI

x=round(summary(model)$coef,4)
round(x,4)
y=data.frame(  RR=exp(x[,1]),
               LB95=exp(x[,1]- qnorm(0.975)*x[,4]),
               UB95=exp(x[,1]+ qnorm(0.975)*x[,4]))
round(y,2)
```

```
> model = gee (whosevere ~
+                              factor(quarter) +
+                              I(dprenatalco/predcoiqr),
+                              data= er,
+                              id=hhid,
+                              family=binomial(link="log"),
+                              corstr="exchangeable")

Beginning Cgee S-function, @(#) geeformula.q 4.13 98/01/27
running glm to get initial regression estimate
             (Intercept)        factor(quarter)2         factor(quarter)3
             -2.81463891             -1.89677133              -1.81774232
        factor(quarter)4 I(dprenatalco/predcoiqr)
             -1.77783546             -0.02443834
>
> # Calculate RR and 95% CI
```

© 2025 McCracken JP et al. *JAMA Network Open*.

```
>
> x=round(summary(model)$coef,4)
> round(x,4)
                          Estimate Naive S.E.  Naive z Robust S.E. Robust z
(Intercept)                -2.8154     0.0850 -33.1051      0.0816 -34.4971
factor(quarter)2           -1.8922     0.2053  -9.2154      0.2037  -9.2878
factor(quarter)3           -1.8118     0.1985  -9.1256      0.1991  -9.1005
factor(quarter)4           -1.7700     0.1953  -9.0620      0.1948  -9.0873
I(dprenatalco/predcoiqr)   -0.0237     0.0397  -0.5971      0.0339  -0.6992
> y=data.frame(       RR=exp(x[,1]),
+                     LB95=exp(x[,1]- qnorm(0.975)*x[,4]),
+                     UB95=exp(x[,1]+ qnorm(0.975)*x[,4]))
> round(y,2)
                            RR LB95 UB95
(Intercept)               0.06 0.05 0.07
factor(quarter)2          0.15 0.10 0.22
factor(quarter)3          0.16 0.11 0.24
factor(quarter)4          0.17 0.12 0.25
I(dprenatalco/predcoiqr) 0.98 0.91 1.04
```

```
# Post-natal PM
model = gee (whosevere ~
                      factor(quarter) +
                      I(postnatalco/postcoiqr),
                      data= er,
                      id=hhid,
                      family=binomial(link="log"),
                      corstr="exchangeable")

# Calculate RR and 95% CI
x=round(summary(model)$coef,4)
round(x,4)
y=data.frame(  RR=exp(x[,1]),
               LB95=exp(x[,1]- qnorm(0.975)*x[,4]),
               UB95=exp(x[,1]+ qnorm(0.975)*x[,4]))
round(y,2)
```

```
> model = gee (whosevere ~
+                              factor(quarter) +
+                              I(postnatalco/postcoiqr),
+                              data= er,
+                              id=hhid,
+                              family=binomial(link="log"),
+                              corstr="exchangeable")

Beginning Cgee S-function, @(#) geeformula.q 4.13 98/01/27
running glm to get initial regression estimate
             (Intercept)         factor(quarter)2         factor(quarter)3
             -2.78870275              -1.86748163              -1.82780809
        factor(quarter)4 I(postnatalco/postcoiqr)
             -1.77364012              -0.04537725
>
> # Calculate RR and 95% CI
> x=round(summary(model)$coef,4)
> round(x,4)
```

© 2025 McCracken JP et al. *JAMA Network Open*.

```
                          Estimate Naive S.E.  Naive z Robust S.E. Robust z
(Intercept)                -2.7913     0.0814 -34.3011      0.0790 -35.3551
factor(quarter)2           -1.8646     0.2053  -9.0835      0.2045  -9.1162
factor(quarter)3           -1.8235     0.2017  -9.0396      0.2031  -8.9766
factor(quarter)4           -1.7670     0.1953  -9.0462      0.1958  -9.0224
I(postnatalco/postcoiqr)  -0.0428     0.0390  -1.0961      0.0357  -1.1983
> y=data.frame(       RR=exp(x[,1]),
+                     LB95=exp(x[,1]- qnorm(0.975)*x[,4]),
+                     UB95=exp(x[,1]+ qnorm(0.975)*x[,4]))

> round(y,2)
                            RR LB95 UB95
(Intercept)               0.06 0.05 0.07
factor(quarter)2          0.15 0.10 0.23
factor(quarter)3          0.16 0.11 0.24
factor(quarter)4          0.17 0.12 0.25
I(postnatalco/postcoiqr) 0.96 0.89 1.03
```

```
## Table 3
##
## Models for severe infant pneumonia and categories of PM2.5

model = gee (pneumonia ~
                      factor(quarter) +
                      dprenatalpmcat +
                      postnatalpmcat +
                      crowding +
                      ma0fe1 +
                      birthweight +
                      winter +
                      covid +
                      sescat +
                      pcv +
                      irc,
                      data= er,
                      id=hhid,
                      family=binomial(link="log"),
                      corstr="exchangeable")

x=round(summary(model)$coef,4)
round(x,4)
y=data.frame(  RR=exp(x[,1]),
               LB95=exp(x[,1]- qnorm(0.975)*x[,4]),
               UB95=exp(x[,1]+ qnorm(0.975)*x[,4]))
round(y,2)
```

```
> model = gee (pneumonia ~
+                            factor(quarter) +
+                            dprenatalpmcat +
+                            postnatalpmcat +
+                            crowding +
+                            ma0fe1 +
+                            birthweight +
+                            winter +
+                            covid +
```

© 2025 McCracken JP et al. *JAMA Network Open*.

```
+                                sescat +
+                                pcv +
+                                irc,
+                                data= er,
+                                id=hhid,
+                                family=binomial(link="log"),
+                                corstr="exchangeable")
Beginning Cgee S-function, @(#) geeformula.q 4.13 98/01/27
running glm to get initial regression estimate
                  (Intercept)             factor(quarter)2
                -1.1763917212                -0.9991771957
             factor(quarter)3             factor(quarter)4
                -1.1339988928                -0.9200951450
      dprenatalpmcat(39.5,66]       dprenatalpmcat(66,109]
                -0.0235334421                 0.1054144621
 dprenatalpmcat(109,1.09e+03]     postnatalpmcat(21.4,37.8]
                 0.2889632180                 0.0436529088
    postnatalpmcat(37.8,74.5] postnatalpmcat(74.5,1.21e+03]
                -0.0950407708                -0.0923042889
                 crowding(3,4]                crowding(4,5]
                 0.0370159829                 0.3201925392
               crowding(5,18]                      ma0fe1
                 0.1183774040                -0.0923484714
                  birthweight                       winter
                -0.0006691009                 0.5800238963
                        covid          sescat(0.198,0.353]
                -0.1539227265                -0.1175784413
          sescat(0.353,0.529]              sescat(0.529,1]
                -0.4819204445                 0.2164558081
                          pcv                    ircIndia
                -0.5488888131                -2.7108560360
                      ircPeru                   ircRwanda
                -1.2846973093                 0.6646651822

> x=round(summary(model)$coef,4)
> round(x,4)
                              Estimate Naive S.E. Naive z Robust S.E. Robust z
(Intercept)                    -1.1322     0.6051 -1.8711      0.7892  -1.4345
factor(quarter)2               -0.9957     0.2000 -4.9779      0.2208  -4.5106
factor(quarter)3               -1.1337     0.2178 -5.2057      0.2575  -4.4034
factor(quarter)4               -0.9263     0.2144 -4.3206      0.2578  -3.5935
dprenatalpmcat(39.5,66]        -0.0185     0.2485 -0.0745      0.2639  -0.0702
dprenatalpmcat(66,109]          0.1058     0.2478  0.4268      0.2604   0.4061
dprenatalpmcat(109,1.09e+03]    0.2852     0.2611  1.0926      0.2742   1.0402
postnatalpmcat(21.4,37.8]       0.0561     0.2502  0.2241      0.2702   0.2075
postnatalpmcat(37.8,74.5]      -0.0961     0.2652 -0.3625      0.2957  -0.3250
postnatalpmcat(74.5,1.21e+03]  -0.0906     0.2709 -0.3346      0.3004  -0.3017
crowding(3,4]                   0.0509     0.2008  0.2536      0.2239   0.2275
crowding(4,5]                   0.3215     0.2194  1.4650      0.2385   1.3477
crowding(5,18]                  0.1386     0.2050  0.6759      0.2266   0.6114
ma0fe1                         -0.0995     0.1466 -0.6785      0.1632  -0.6095
birthweight                    -0.0007     0.0002 -3.8723      0.0002  -2.9694
winter                          0.5768     0.2143  2.6916      0.2444   2.3599
covid                          -0.1272     0.1627 -0.7819      0.2066  -0.6158
sescat(0.198,0.353]            -0.1287     0.1961 -0.6563      0.2165  -0.5945
sescat(0.353,0.529]            -0.5085     0.2826 -1.7996      0.3374  -1.5071
sescat(0.529,1]                 0.2002     0.3866  0.5178      0.3719   0.5383
pcv                            -0.5675     0.1884 -3.0122      0.2036  -2.7867
ircIndia                       -2.7053     0.4705 -5.7501      0.4592  -5.8918
ircPeru                        -1.2744     0.3301 -3.8605      0.3253  -3.9181
ircRwanda                       0.6461     0.2539  2.5453      0.3138   2.0593
```

© 2025 McCracken JP et al. *JAMA Network Open*.

```
> y=data.frame(       RR=exp(x[,1]),
+                     LB95=exp(x[,1]- qnorm(0.975)*x[,4]),
+                     UB95=exp(x[,1]+ qnorm(0.975)*x[,4]))
> round(y,2)
                                RR LB95 UB95
(Intercept)                   0.32 0.07 1.51
factor(quarter)2              0.37 0.24 0.57
factor(quarter)3              0.32 0.19 0.53
factor(quarter)4              0.40 0.24 0.66
dprenatalpmcat(39.5,66]       0.98 0.59 1.65
dprenatalpmcat(66,109]        1.11 0.67 1.85
dprenatalpmcat(109,1.09e+03]  1.33 0.78 2.28
postnatalpmcat(21.4,37.8]     1.06 0.62 1.80
postnatalpmcat(37.8,74.5]     0.91 0.51 1.62
postnatalpmcat(74.5,1.21e+03] 0.91 0.51 1.65
crowding(3,4]                 1.05 0.68 1.63
crowding(4,5]                 1.38 0.86 2.20
crowding(5,18]                1.15 0.74 1.79
ma0fe1                        0.91 0.66 1.25
birthweight                   1.00 1.00 1.00
winter                        1.78 1.10 2.87
covid                         0.88 0.59 1.32
sescat(0.198,0.353]           0.88 0.58 1.34
sescat(0.353,0.529]           0.60 0.31 1.17
sescat(0.529,1]               1.22 0.59 2.53
pcv                           0.57 0.38 0.84
ircIndia                      0.07 0.03 0.16
ircPeru                       0.28 0.15 0.53
ircRwanda                     1.91 1.03 3.53
```

```
## Unadjusted models
# Prenatal PM2.5 categories
model = gee (pneumonia ~
                      factor(quarter) +
                      dprenatalpmcat,
                      data= er,
                      id=hhid,
                      family=binomial(link="log"),
                      corstr="exchangeable")

# Calculate RR and 95% CI
x=round(summary(model)$coef,4)
round(x,4)
y=data.frame(  RR=exp(x[,1]),
               LB95=exp(x[,1]- qnorm(0.975)*x[,4]),
               UB95=exp(x[,1]+ qnorm(0.975)*x[,4]))
round(y,2)
```

```
> model = gee ( pneumonia ~
+                         factor(quarter) +
+                         dprenatalpmcat,
+                         data= er,
+                         id=hhid,
+                         family=binomial(link="log"),
```

© 2025 McCracken JP et al. *JAMA Network Open*.

```
+                              corstr="exchangeable")

Beginning Cgee S-function, @(#) geeformula.q 4.13 98/01/27
running glm to get initial regression estimate
                 (Intercept)            factor(quarter)2
                  -3.9383737                  -1.1696131
            factor(quarter)3            factor(quarter)4
                  -1.3253575                  -1.0548015
     dprenatalpmcat(39.5,66]      dprenatalpmcat(66,109]
                   0.2523592                   0.4987624
dprenatalpmcat(109,1.09e+03]
                   0.7445850

> # Calculate RR and 95% CI
> x=round(summary(model)$coef,4)
> round(x,4)
                             Estimate Naive S.E.  Naive z Robust S.E. Robust z
(Intercept)                   -3.9373     0.2049 -19.2192      0.1983 -19.8526
factor(quarter)2              -1.1587     0.2124  -5.4544      0.2113  -5.4848
factor(quarter)3              -1.3082     0.2259  -5.7909      0.2299  -5.6913
factor(quarter)4              -1.0392     0.2036  -5.1035      0.2041  -5.0904
dprenatalpmcat(39.5,66]        0.2576     0.2571   1.0021      0.2532   1.0175
dprenatalpmcat(66,109]         0.4953     0.2448   2.0227      0.2404   2.0600
dprenatalpmcat(109,1.09e+03]   0.7412     0.2342   3.1642      0.2309   3.2104

> y=data.frame(       RR=exp(x[,1]),
+                     LB95=exp(x[,1]- qnorm(0.975)*x[,4]),
+                     UB95=exp(x[,1]+ qnorm(0.975)*x[,4]))
> round(y,2)
                               RR LB95 UB95
(Intercept)                  0.02 0.01 0.03
factor(quarter)2             0.31 0.21 0.47
factor(quarter)3             0.27 0.17 0.42
factor(quarter)4             0.35 0.24 0.53
dprenatalpmcat(39.5,66]      1.29 0.79 2.13
dprenatalpmcat(66,109]       1.64 1.02 2.63
dprenatalpmcat(109,1.09e+03] 2.10 1.33 3.30
```

```
# Postnatal PM2.5 categoreis
model = gee (pneumonia ~
                      factor(quarter) +
                      postnatalpmcat,
                      data= er,
                      id=hhid,
                      family=binomial(link="log"),
                      corstr="exchangeable")

# Calculate RR and 95% CI

x=round(summary(model)$coef,4)
round(x,4)
y=data.frame(  RR=exp(x[,1]),
               LB95=exp(x[,1]- qnorm(0.975)*x[,4]),
               UB95=exp(x[,1]+ qnorm(0.975)*x[,4]))
round(y,2)
```

© 2025 McCracken JP et al. *JAMA Network Open*.

```
> model = gee ( pneumonia ~
+                           factor(quarter) +
+                           postnatalpmcat,
+                           data= er,
+                           id=hhid,
+                           family=binomial(link="log"),
+                           corstr="exchangeable")

Beginning Cgee S-function, @(#) geeformula.q 4.13 98/01/27
running glm to get initial regression estimate
                  (Intercept)              factor(quarter)2
                   -4.0500295                    -1.1250318
             factor(quarter)3              factor(quarter)4
                   -1.2822610                    -1.0461455
    postnatalpmcat(21.4,37.8]     postnatalpmcat(37.8,74.5]
                    0.5387212                     0.6269370
postnatalpmcat(74.5,1.21e+03]
                    0.6923070
>
> # Calculate RR and 95% CI
>
> x=round(summary(model)$coef,4)
> round(x,4)
                              Estimate Naive S.E.  Naive z Robust S.E. Robust z
(Intercept)                    -4.0415     0.2094 -19.2994      0.2026 -19.9525
factor(quarter)2               -1.1170     0.2147  -5.2025      0.2134  -5.2330
factor(quarter)3               -1.2681     0.2281  -5.5590      0.2319  -5.4682
factor(quarter)4               -1.0309     0.2057  -5.0112      0.2054  -5.0181
postnatalpmcat(21.4,37.8]       0.5407     0.2504   2.1595      0.2458   2.2000
postnatalpmcat(37.8,74.5]       0.6060     0.2494   2.4299      0.2431   2.4932
postnatalpmcat(74.5,1.21e+03]   0.6775     0.2458   2.7564      0.2407   2.8146

> y=data.frame(       RR=exp(x[,1]),
+                     LB95=exp(x[,1]- qnorm(0.975)*x[,4]),
+                     UB95=exp(x[,1]+ qnorm(0.975)*x[,4]))
> round(y,2)
                                RR LB95 UB95
(Intercept)                   0.02 0.01 0.03
factor(quarter)2              0.33 0.22 0.50
factor(quarter)3              0.28 0.18 0.44
factor(quarter)4              0.36 0.24 0.53
postnatalpmcat(21.4,37.8]     1.72 1.06 2.78
postnatalpmcat(37.8,74.5]     1.83 1.14 2.95
postnatalpmcat(74.5,1.21e+03] 1.97 1.23 3.16
```

```
# Models for severe infant pneumonia and categories of CO

model = gee ( pneumonia ~
                      factor(quarter) +
                      dprenatalcocat +
                      postnatalcocat +
                      crowding +
                      ma0fe1 +
                      birthweight +
```

© 2025 McCracken JP et al. *JAMA Network Open*.

```
                              winter +
                              covid +
                              sescat +
                              pcv +
                              irc,
                              data= er,
                              id=hhid,
                              family=binomial(link="log"),
                              corstr="exchangeable")

# Calculate RR and 95% CI
x=round(summary(model)$coef,4)
round(x,4)
y=data.frame(  RR=exp(x[,1]),
               LB95=exp(x[,1]- qnorm(0.975)*x[,4]),
               UB95=exp(x[,1]+ qnorm(0.975)*x[,4]))
round(y,2)
```

```
model = gee ( pneumonia ~
+                              factor(quarter) +
+                              dprenatalcocat +
+                              postnatalcocat +
+                              crowding +
+                              ma0fe1 +
+                              birthweight +
+                              winter +
+                              covid +
+                              sescat +
+                              pcv +
+                              irc,
+                              data= er,
+                              id=hhid,
+                              family=binomial(link="log"),
+                              corstr="exchangeable")

Beginning Cgee S-function, @(#) geeformula.q 4.13 98/01/27
running glm to get initial regression estimate
                (Intercept)            factor(quarter)2
              -1.1893281689               -1.0581358193
           factor(quarter)3            factor(quarter)4
              -1.1522569969               -0.9410700041
 dprenatalcocat(0.531,1.07]   dprenatalcocat(1.07,2.16]
               0.6368230271                0.3210387670
  dprenatalcocat(2.16,46.4] postnatalcocat(0.204,0.717]
               0.2627240581               -0.0942175562
 postnatalcocat(0.717,1.75]   postnatalcocat(1.75,98.5]
               0.1125000247               -0.0641361868
              crowding(3,4]               crowding(4,5]
               0.0233562186                0.2951262750
             crowding(5,18]                      ma0fe1
               0.0806834460               -0.0957801530
                birthweight                      winter
              -0.0007228277                0.5008320444
                      covid         sescat(0.198,0.353]
              -0.1518095123               -0.0930923401
        sescat(0.353,0.529]             sescat(0.529,1]
```

© 2025 McCracken JP et al. *JAMA Network Open*.

```
             -0.4905152634              0.2047758915
                       pcv                 ircIndia
             -0.5375596305             -2.6993416103
                  ircPeru                 ircRwanda
             -1.3831487062              0.5719868973
>
> # Calculate RR and 95% CI
> x=round(summary(model)$coef,4)
> round(x,4)
                             Estimate Naive S.E. Naive z Robust S.E. Robust z
(Intercept)                   -1.1479     0.5644 -2.0338      0.7509  -1.5286
factor(quarter)2              -1.0543     0.2037 -5.1766      0.2240  -4.7071
factor(quarter)3              -1.1523     0.2191 -5.2584      0.2596  -4.4394
factor(quarter)4              -0.9468     0.2165 -4.3725      0.2589  -3.6564
dprenatalcocat(0.531,1.07]     0.6552     0.2150  3.0468      0.2320   2.8237
dprenatalcocat(1.07,2.16]      0.3404     0.2379  1.4307      0.2556   1.3315
dprenatalcocat(2.16,46.4]      0.2729     0.2622  1.0408      0.3030   0.9008
postnatalcocat(0.204,0.717]   -0.1073     0.2083 -0.5154      0.2163  -0.4962
postnatalcocat(0.717,1.75]     0.0964     0.2052  0.4696      0.2241   0.4300
postnatalcocat(1.75,98.5]     -0.0809     0.2431 -0.3329      0.2778  -0.2914
crowding(3,4]                  0.0362     0.2008  0.1805      0.2216   0.1635
crowding(4,5]                  0.2934     0.2227  1.3170      0.2415   1.2146
crowding(5,18]                 0.0975     0.2086  0.4675      0.2311   0.4222
ma0fe1                        -0.1035     0.1468 -0.7048      0.1648  -0.6279
birthweight                   -0.0007     0.0002 -4.1895      0.0002  -3.1987
winter                         0.4990     0.2126  2.3471      0.2427   2.0559
covid                         -0.1273     0.1646 -0.7734      0.2094  -0.6081
sescat(0.198,0.353]           -0.1014     0.1961 -0.5171      0.2212  -0.4585
sescat(0.353,0.529]           -0.5126     0.2817 -1.8196      0.3423  -1.4976
sescat(0.529,1]                0.1917     0.3793  0.5055      0.3620   0.5296
pcv                           -0.5541     0.1932 -2.8682      0.2126  -2.6064
ircIndia                      -2.6953     0.4699 -5.7365      0.4530  -5.9503
ircPeru                       -1.3766     0.3442 -3.9995      0.3639  -3.7826
ircRwanda                      0.5559     0.2483  2.2385      0.3087   1.8010

> y=data.frame(       RR=exp(x[,1]),
+                     LB95=exp(x[,1]- qnorm(0.975)*x[,4]),
+                     UB95=exp(x[,1]+ qnorm(0.975)*x[,4]))
> round(y,2)
                              RR LB95 UB95
(Intercept)                 0.32 0.07 1.38
factor(quarter)2            0.35 0.22 0.54
factor(quarter)3            0.32 0.19 0.53
factor(quarter)4            0.39 0.23 0.64
dprenatalcocat(0.531,1.07]  1.93 1.22 3.03
dprenatalcocat(1.07,2.16]   1.41 0.85 2.32
dprenatalcocat(2.16,46.4]   1.31 0.73 2.38
postnatalcocat(0.204,0.717] 0.90 0.59 1.37
postnatalcocat(0.717,1.75]  1.10 0.71 1.71
postnatalcocat(1.75,98.5]   0.92 0.54 1.59
crowding(3,4]               1.04 0.67 1.60
crowding(4,5]               1.34 0.84 2.15
crowding(5,18]              1.10 0.70 1.73
ma0fe1                      0.90 0.65 1.25
birthweight                 1.00 1.00 1.00
winter                      1.65 1.02 2.65
covid                       0.88 0.58 1.33
sescat(0.198,0.353]         0.90 0.59 1.39
sescat(0.353,0.529]         0.60 0.31 1.17
```

© 2025 McCracken JP et al. *JAMA Network Open*.

```
sescat(0.529,1]              1.21 0.60 2.46
pcv                          0.57 0.38 0.87
ircIndia                     0.07 0.03 0.16
ircPeru                      0.25 0.12 0.52
ircRwanda                    1.74 0.95 3.19
```

```
# Unadjusted models
# Prenatal CO categories
model = gee (pneumonia ~
                       factor(quarter) +
                       dprenatalcocat,
                       data= er, id=hhid,
                       family=binomial(link="log"),
                       corstr="exchangeable")

# Calculate RR and 95% CI
x=round(summary(model)$coef,4)
round(x,4)
y=data.frame(  RR=exp(x[,1]),
               LB95=exp(x[,1]- qnorm(0.975)*x[,4]),
               UB95=exp(x[,1]+ qnorm(0.975)*x[,4]))
round(y,2)
```

```
> model = gee (pneumonia ~
+                            factor(quarter) +
+                            dprenatalcocat,
+                            data= er, id=hhid,
+                            family=binomial(link="log"),
+                            corstr="exchangeable")

Beginning Cgee S-function, @(#) geeformula.q 4.13 98/01/27
running glm to get initial regression estimate
              (Intercept)         factor(quarter)2          factor(quarter)3
               -3.8698435               -1.1835377                -1.3393599
         factor(quarter)4 dprenatalcocat(0.531,1.07] dprenatalcocat(1.07,2.16]
               -1.0690574                0.7143494                 0.3427347
 dprenatalcocat(2.16,46.4]
                0.2116560
>
> # Calculate RR and 95%CI
> x=round(summary(model)$coef,4)
> round(x,4)
                          Estimate Naive S.E.  Naive z Robust S.E.  Robust z
(Intercept)                -3.8857     0.2043 -19.0165      0.1920 -20.2390
factor(quarter)2           -1.1696     0.2141  -5.4627      0.2103  -5.5607
factor(quarter)3           -1.3175     0.2276  -5.7880      0.2286  -5.7641
factor(quarter)4           -1.0490     0.2050  -5.1170      0.2031  -5.1655
dprenatalcocat(0.531,1.07]  0.7358     0.2341   3.1434      0.2292   3.2107
dprenatalcocat(1.07,2.16]   0.3652     0.2510   1.4549      0.2477   1.4743
dprenatalcocat(2.16,46.4]   0.2238     0.2586   0.8652      0.2602   0.8600

> y=data.frame(       RR=exp(x[,1]),
+                     LB95=exp(x[,1]- qnorm(0.975)*x[,4]),
+                     UB95=exp(x[,1]+ qnorm(0.975)*x[,4]))
> round(y,2)
                              RR LB95 UB95
(Intercept)                 0.02 0.01 0.03
```

© 2025 McCracken JP et al. *JAMA Network Open*.

```
factor(quarter)2            0.31 0.21 0.47
factor(quarter)3            0.27 0.17 0.42
factor(quarter)4            0.35 0.24 0.52
dprenatalcocat(0.531,1.07] 2.09 1.33 3.27
dprenatalcocat(1.07,2.16]  1.44 0.89 2.34
dprenatalcocat(2.16,46.4]  1.25 0.75 2.08
```

```
# Postnatal CO categories
model = gee (pneumonia ~
                      factor(quarter) +
                      postnatalcocat,
                      data= er,
                      id=hhid,
                      family=binomial(link="log"),
                      corstr="exchangeable")

x=round(summary(model)$coef,4)
round(x,4)
y=data.frame(  RR=exp(x[,1]),
               LB95=exp(x[,1]- qnorm(0.975)*x[,4]),
               UB95=exp(x[,1]+ qnorm(0.975)*x[,4]))
round(y,2)
```

```
> model = gee (pneumonia ~
+                            factor(quarter) +
+                            postnatalcocat,
+                            data= er,
+                            id=hhid,
+                            family=binomial(link="log"),
+                            corstr="exchangeable")

Beginning Cgee S-function, @(#) geeformula.q 4.13 98/01/27
running glm to get initial regression estimate
                (Intercept)            factor(quarter)2
                 -3.6347191                  -1.1733346
           factor(quarter)3            factor(quarter)4
                 -1.2944017                  -1.0528815
postnatalcocat(0.204,0.717]  postnatalcocat(0.717,1.75]
                  0.1606901                   0.3358306
  postnatalcocat(1.75,98.5]
                 -0.1272953
>
> x=round(summary(model)$coef,4)
> round(x,4)
                             Estimate Naive S.E.  Naive z Robust S.E. Robust z
(Intercept)                   -3.6214     0.1727 -20.9741      0.1719 -21.0660
factor(quarter)2              -1.1645     0.2181  -5.3392      0.2154  -5.4065
factor(quarter)3              -1.2804     0.2285  -5.6038      0.2326  -5.5037
factor(quarter)4              -1.0392     0.2065  -5.0326      0.2061  -5.0431
postnatalcocat(0.204,0.717]    0.1417     0.2244   0.6314      0.2136   0.6633
postnatalcocat(0.717,1.75]     0.3155     0.2163   1.4587      0.2155   1.4641
postnatalcocat(1.75,98.5]     -0.1446     0.2426  -0.5961      0.2406  -0.6011

> y=data.frame(       RR=exp(x[,1]),
+                     LB95=exp(x[,1]- qnorm(0.975)*x[,4]),
```

© 2025 McCracken JP et al. *JAMA Network Open*.

```
+                       UB95=exp(x[,1]+ qnorm(0.975)*x[,4]))
> round(y,2)
                               RR LB95 UB95
(Intercept)                  0.03 0.02 0.04
factor(quarter)2             0.31 0.20 0.48
factor(quarter)3             0.28 0.18 0.44
factor(quarter)4             0.35 0.24 0.53
postnatalcocat(0.204,0.717] 1.15 0.76 1.75
postnatalcocat(0.717,1.75]  1.37 0.90 2.09
postnatalcocat(1.75,98.5]   0.87 0.54 1.39
```

```
# Calculate Incidence of severe infant pneumonia: # episode/# infant-
 quarters.
rate_dprenatalpm <- tapply(er$pneumonia, er$dprenatalpmcat, sum, na.rm =
 TRUE) / table(er$dprenatalpmcat, useNA = "no")
round(4*rate_dprenatalpm*100, 2)
rate_postnatalpm <- tapply(er$pneumonia, er$postnatalpmcat, sum, na.rm =
 TRUE) / table(er$postnatalpmcat, useNA = "no")
round(4*rate_postnatalpm*100, 2)
rate_dprenatalco <- tapply(er$pneumonia, er$dprenatalcocat, sum, na.rm =
 TRUE) / table(er$dprenatalcocat, useNA = "no")
round(4*rate_dprenatalco*100, 2)
rate_postnatalco <- tapply(er$pneumonia, er$postnatalcocat, sum, na.rm =
 TRUE) / table(er$postnatalcocat, useNA = "no")
round(4*rate_postnatalco*100, 2)
```

```
> rate_dprenatalpm <- tapply(er$pneumonia, er$dprenatalpmcat, sum, na.rm =
TRUE) / table(er$dprenatalpmcat, useNA = "no")
> round(4*rate_dprenatalpm*100, 2)
   [10.7,39.5]      (39.5,66]       (66,109] (109,1.09e+03]
          3.78           4.86           6.21           7.97
> rate_postnatalpm <- tapply(er$pneumonia, er$postnatalpmcat, sum, na.rm =
TRUE) / table(er$postnatalpmcat, useNA = "no")
> round(4*rate_postnatalpm*100, 2)
     [9.8,21.4]     (21.4,37.8]     (37.8,74.5] (74.5,1.21e+03]
           3.54            5.86            6.28            6.82
> rate_dprenatalco <- tapply(er$pneumonia, er$dprenatalcocat, sum, na.rm =
TRUE) / table(er$dprenatalcocat, useNA = "no")
> round(4*rate_dprenatalco*100, 2)
   [0,0.531] (0.531,1.07]  (1.07,2.16]  (2.16,46.4]
        4.03         8.20         5.64         4.97
> rate_postnatalco <- tapply(er$pneumonia, er$postnatalcocat, sum, na.rm =
TRUE) / table(er$postnatalcocat, useNA = "no")
> round(4*rate_postnatalco*100, 2)
    [0,0.204] (0.204,0.717]  (0.717,1.75]   (1.75,98.5]
         5.42          5.85          6.96          4.45
```

```
## Table 4
## Subgroup analyses
## Calculate RR and 95% CIs using 1000 bootstrap replications
#
# Sex

basemodel = gee (pneumonia ~
```

© 2025 McCracken JP et al. *JAMA Network Open*.

```
                              factor(quarter) +
                              I(dprenatalpm/predpmiqr)*ma0fe1 +
                              I(postnatalpm/postpmiqr)*ma0fe1 +
                              crowding +
                              birthweight +
                              pcv +
                              covid +
                              winter +
                              sescat +
                              irc,
                              data= er, id=hhid,
                              family=binomial(link="log"),
                              corstr="exchangeable")


set.seed(125)  # For reproducibility
n_bootstrap <- 1110
#bootstrap_results = replicate(n_bootstrap, boot_gee(er), simplify=FALSE)
#bootstrap_df <- do.call(rbind, bootstrap_results)

# Pre-allocate a list to store coefficients from successful fits
results_list <- vector("list", n_bootstrap)

for (i in seq_len(n_bootstrap)) {
  # 1) Resample clusters with replacement
  boothhid <- sample(uhhid, size = length(uhhid), replace = TRUE)
  hhidtable <- data.frame(hhid = boothhid, newhhid = seq_along(boothhid))
  resampled_data <- merge(er, hhidtable, by = "hhid", sort = FALSE)

  # 2) Fit the logistic regression (glm) and catch errors or non-convergence
  smodel <- try(
    suppressWarnings(
      glm(
        pneumonia ~ factor(quarter) +
          I(dprenatalpm / predpmiqr) * ma0fe1 +
          I(postnatalpm  / postpmiqr) * ma0fe1 +
          crowding + birthweight +
          pcv + covid + winter +
          sescat + irc,
        data   = resampled_data,
        family = binomial(link = "log")
      )
    ),
    silent = TRUE
  )
  if (inherits(smodel, "try-error") || !isTRUE(smodel$converged)) {
    print(paste(i,
                if (inherits(smodel, "try-error")) "glm_error" else
 smodel$converged,
                NA,
                Sys.time()))
    results_list[[i]] <- NULL
    next
  }

  # 3) Fit the GEE model and catch errors or non-convergence
```

© 2025 McCracken JP et al. *JAMA Network Open*.

```
  model <- try(
    suppressWarnings(
      glmgee(
        pneumonia ~ factor(quarter) +
          I(dprenatalpm / predpmiqr) * ma0fe1 +
          I(postnatalpm  / postpmiqr) * ma0fe1 +
          crowding + birthweight +
          pcv + covid + winter +
          sescat + irc,
        data    = resampled_data,
        id      = newhhid,
        family  = binomial(link = "log"),
        corstr  = "exchangeable"
      )
    ),
    silent = TRUE
  )
  if (inherits(model, "try-error") ||
      !is.logical(model$converged) ||
      !isTRUE(model$converged)
  ) {
    print(paste(i,
                smodel$converged,
                if (inherits(model, "try-error")) "gee_error" else
 model$converged,
                Sys.time()))
    results_list[[i]] <- NULL
    next
  }

  # 4) On success, print iteration info and save coefficients
  print(paste(i, smodel$converged, model$converged, Sys.time()))
  results_list[[i]] <- as.numeric(model$coef)
}

# Combine all successful coefficient vectors into one data frame
bootstrap_df <- do.call(rbind, Filter(Negate(is.null), results_list))

subgroup_sex <- head(bootstrap_df, 1000)

# Calculate RR and percentile-based bootstrap 95% CIs
mpre0          = basemodel$coef[5]
mpos0          = basemodel$coef[7]
mpre1          = basemodel$coef[5] + basemodel$coef[21]
mpos1          = basemodel$coef[7] + basemodel$coef[22]
mpre0lb        = quantile ( subgroup_sex[,5], 0.025)
mpre0ub        = quantile ( subgroup_sex[,5], 0.975)
mpos0lb        = quantile ( subgroup_sex[,7], 0.025)
mpos0ub        = quantile ( subgroup_sex[,7], 0.975)

mpre1lb= quantile ( subgroup_sex[,5] + subgroup_sex[,21], 0.025)
mpre1ub= quantile ( subgroup_sex[,5] + subgroup_sex[,21], 0.975)
mpos1lb= quantile ( subgroup_sex[,7] + subgroup_sex[,22], 0.025)
mpos1ub = quantile ( subgroup_sex[,7] + subgroup_sex[,22], 0.975)

# Print RRs
```

© 2025 McCracken JP et al. *JAMA Network Open*.

```
round(exp (
rbind (        c(mpre0, mpre0lb, mpre0ub),
               c(mpos0, mpos0lb, mpos0ub),
               c(mpre1, mpre1lb, mpre1ub),
               c(mpos1, mpos1lb, mpos1ub))),2)
```

```
> subgroup_sex <- head(bootstrap_df, 1000)

> mpre0 = basemodel$coef[5]
> mpos0 = basemodel$coef[7]
> mpre1 = basemodel$coef[5] + basemodel$coef[21]
> mpos1 = basemodel$coef[7] + basemodel$coef[22]
> mpre0lb = quantile ( subgroup_sex[,5], 0.025)
> mpre0ub = quantile ( subgroup_sex[,5], 0.975)
> mpos0lb = quantile ( subgroup_sex[,7], 0.025)
> mpos0ub = quantile ( subgroup_sex[,7], 0.975)
>
> mpre1lb = quantile ( subgroup_sex[,5] + subgroup_sex[,21], 0.025)
> mpre1ub = quantile ( subgroup_sex[,5] + subgroup_sex[,21], 0.975)
> mpos1lb = quantile ( subgroup_sex[,7] + subgroup_sex[,22], 0.025)
> mpos1ub = quantile ( subgroup_sex[,7] + subgroup_sex[,22], 0.975)
>
> # Print RRs
>
> round(exp (
+ rbind (      c(mpre0, mpre0lb, mpre0ub),
+                      c(mpos0, mpos0lb, mpos0ub),
+                      c(mpre1, mpre1lb, mpre1ub),
+                      c(mpos1, mpos1lb, mpos1ub))),2)

                         RR  2.5% 97.5%
[1,]                    1.07 0.81  1.33
[2,]                    0.84 0.60  1.04
[3,]                    1.02 0.82  1.09
[4,]                    1.05 0.92  1.15
```

```
# Winter

basemodel = gee (pneumonia ~
                       factor(quarter) +
                       I(dprenatalpm/predpmiqr)*winter +
                       I(postnatalpm/postpmiqr)*winter +
                       crowding +
                       birthweight +
                       pcv +
                       covid +
                       ma0fe1 +
                       sescat +
                       irc,
                       data= er,
                       id=hhid,
                       family=binomial(link="log"),
                       corstr="exchangeable")
```

© 2025 McCracken JP et al. *JAMA Network Open*.

```
set.seed(125)  # For reproducibility
n_bootstrap <- 1050
#bootstrap_results = replicate(n_bootstrap, boot_gee(er), simplify=FALSE)
#bootstrap_df <- do.call(rbind, bootstrap_results)

# Pre-allocate a list to store coefficients from successful fits
results_list <- vector("list", n_bootstrap)

for (i in seq_len(n_bootstrap)) {
  # 1) Resample clusters with replacement
  boothhid <- sample(uhhid, size = length(uhhid), replace = TRUE)
  hhidtable <- data.frame(hhid = boothhid, newhhid = seq_along(boothhid))
  resampled_data <- merge(er, hhidtable, by = "hhid", sort = FALSE)

  # 2) Fit the logistic regression (glm) and catch errors or non-convergence
  smodel <- try(
    suppressWarnings(
      glm ( pneumonia ~ factor(quarter) +
              I(dprenatalpm/predpmiqr)*winter +
              I(postnatalpm/postpmiqr)*winter +
              crowding +
              birthweight +
              pcv +
              covid +
              ma0fe1 +
              sescat +
              irc,
            data= resampled_data,
            family=binomial(link="log"))
    ),
    silent = TRUE
  )
  if (inherits(smodel, "try-error") || !isTRUE(smodel$converged)) {
    print(paste(i,
               if (inherits(smodel, "try-error")) "glm_error" else
 smodel$converged,
               NA,
               Sys.time()))
    results_list[[i]] <- NULL
    next
  }

  # 3) Fit the GEE model and catch errors or non-convergence
  model <- try(
    suppressWarnings(
      glmgee (pneumonia ~
               factor(quarter) +
               I(dprenatalpm/predpmiqr)*winter +
               I(postnatalpm/postpmiqr)*winter +
               crowding +
               birthweight +
               pcv +
               covid +
               ma0fe1 +
```

© 2025 McCracken JP et al. *JAMA Network Open*.

```
                sescat +
                irc,
              data= resampled_data,
              id=newhhid,
              family=binomial(link="log"),
              corstr="exchangeable")
    ),
    silent = TRUE
  )
  if (inherits(model, "try-error") ||
      !is.logical(model$converged) ||
      !isTRUE(model$converged)
  ) {
    print(paste(i,
                smodel$converged,
                if (inherits(model, "try-error")) "gee_error" else
 model$converged,
                Sys.time()))
    results_list[[i]] <- NULL
    next
  }

  # 4) On success, print iteration info and save coefficients
  print(paste(i, smodel$converged, model$converged, Sys.time()))
  results_list[[i]] <- as.numeric(model$coef)
}

# Combine all successful coefficient vectors into one data frame
bootstrap_df <- do.call(rbind, Filter(Negate(is.null), results_list))

subgroup_winter <- head(bootstrap_df, 1000)

mpre0          = basemodel$coef[5]
mpos0          = basemodel$coef[7]
mpre1          = basemodel$coef[5] + basemodel$coef[21]
mpos1          = basemodel$coef[7] + basemodel$coef[22]
mpre0lb        = quantile ( subgroup_winter[,5], 0.025)
mpre0ub        = quantile ( subgroup_winter[,5], 0.975)
mpos0lb        = quantile ( subgroup_winter[,7], 0.025)
mpos0ub        = quantile ( subgroup_winter[,7], 0.975)

mpre1lb= quantile ( subgroup_winter[,5] + subgroup_winter[,21], 0.025)
mpre1ub= quantile ( subgroup_winter[,5] + subgroup_winter[,21], 0.975)
mpos1lb= quantile ( subgroup_winter[,7] + subgroup_winter[,22], 0.025)
mpos1ub = quantile ( subgroup_winter[,7] + subgroup_winter[,22], 0.975)

round(exp (
rbind (       c(mpre0, mpre0lb, mpre0ub),
              c(mpos0, mpos0lb, mpos0ub),
              c(mpre1, mpre1lb, mpre1ub),
              c(mpos1, mpos1lb, mpos1ub))),2)
```

```
> subgroup_winter <- head(bootstrap_df, 1000)

> mpre0        = basemodel$coef[5]
```

© 2025 McCracken JP et al. *JAMA Network Open*.

```
> mpos0        = basemodel$coef[7]
> mpre1        = basemodel$coef[5] + basemodel$coef[21]
> mpos1        = basemodel$coef[7] + basemodel$coef[22]
> mpre0lb      = quantile ( subgroup_winter[,5], 0.025)
> mpre0ub      = quantile ( subgroup_winter[,5], 0.975)
> mpos0lb      = quantile ( subgroup_winter[,7], 0.025)
> mpos0ub      = quantile ( subgroup_winter[,7], 0.975)
>
> mpre1lb      = quantile ( subgroup_winter[,5] + subgroup_winter[,21],
0.025)
> mpre1ub      = quantile ( subgroup_winter[,5] + subgroup_winter[,21],
0.975)
> mpos1lb      = quantile ( subgroup_winter[,7] + subgroup_winter[,22],
0.025)
> mpos1ub      = quantile ( subgroup_winter[,7] + subgroup_winter[,22],
0.975)
>
> round(exp (
+ rbind (      c(mpre0, mpre0lb, mpre0ub),
+                      c(mpos0, mpos0lb, mpos0ub),
+                      c(mpre1, mpre1lb, mpre1ub),
+                      c(mpos1, mpos1lb, mpos1ub))),2)

                          RR  2.5% 97.5%
[1,]                     1.07 0.94  1.16
[2,]                     0.93 0.71  1.16
[3,]                     0.88 0.65  1.14
[4,]                     1.05 0.85  1.19
```

```
# PCV

# Pre-allocate a list to store coefficients from successful fits
results_list <- vector("list", n_bootstrap)

for (i in seq_len(n_bootstrap)) {
  if (i == 101) next   # Skip iteration 101

  # 1) Resample clusters with replacement
  boothhid <- sample(uhhid, size = length(uhhid), replace = TRUE)
  hhidtable <- data.frame(hhid = boothhid, newhhid = seq_along(boothhid))
  resampled_data <- merge(er, hhidtable, by = "hhid")

  # 2) Fit the logistic regression (glm) and catch errors or non-convergence
  smodel <- try(
    suppressWarnings(
      glm ( pneumonia ~
              factor(quarter) +
              I(dprenatalpm/predpmiqr)*pcv +
              I(postnatalpm/postpmiqr)*pcv +
              crowding +
              birthweight +
              winter +
              covid +
              ma0fe1 +
              sescat +
              irc,
```

© 2025 McCracken JP et al. *JAMA Network Open*.

```
            data= resampled_data,
            family=binomial(link="log"))
    ),
    silent = TRUE
  )
  if (inherits(smodel, "try-error") || !isTRUE(smodel$converged)) {
    print(paste(i,
                if (inherits(smodel, "try-error")) "glm_error" else
 smodel$converged,
                NA,
                Sys.time()))
    next
  }

  # 3) Fit the GEE model and catch errors or non-convergence
  model <- try(
    suppressWarnings(
      glmgee (pneumonia ~
                factor(quarter) +
                I(dprenatalpm/predpmiqr)*pcv +
                I(postnatalpm/postpmiqr)*pcv +
                crowding +
                birthweight +
                winter +
                covid +
                ma0fe1 +
                sescat +
                irc,
              data= resampled_data,
              id=newhhid, family=binomial(link="log"), corstr="exchangeable")
    ),
    silent = TRUE
  )
  if (inherits(model, "try-error") ||
      !is.logical(model$converged) ||
      !isTRUE(model$converged)
  ) {
    print(paste(i,
                smodel$converged,
                if (inherits(model, "try-error")) "gee_error" else
 model$converged,
                Sys.time()))
    next
  }

  # 4) On success, print iteration info and save coefficients
  print(paste(i, smodel$converged, model$converged, Sys.time()))
  results_list[[i]] <- as.numeric(model$coef)
}

# Combine all successful coefficient vectors into one data frame
bootstrap_df <- do.call(rbind, results_list)

subgroup_pcv <- head(bootstrap_df, 1000)

mpre0          = basemodel$coef[5]
mpos0          = basemodel$coef[7]
```

© 2025 McCracken JP et al. *JAMA Network Open*.

```
mpre1          = basemodel$coef[5] + basemodel$coef[21]
mpos1          = basemodel$coef[7] + basemodel$coef[22]
mpre0lb        = quantile ( subgroup_pcv[,5], 0.025)
mpre0ub        = quantile ( subgroup_pcv[,5], 0.975)
mpos0lb        = quantile ( subgroup_pcv[,7], 0.025)
mpos0ub        = quantile ( subgroup_pcv[,7], 0.975)

mpre1lb        = quantile ( subgroup_pcv[,5] + subgroup_pcv[,21], 0.025)
mpre1ub        = quantile ( subgroup_pcv[,5] + subgroup_pcv[,21], 0.975)
mpos1lb        = quantile ( subgroup_pcv[,7] + subgroup_pcv[,22], 0.025)
mpos1ub        = quantile ( subgroup_pcv[,7] + subgroup_pcv[,22], 0.975)

round(exp (
rbind (        c(mpre0, mpre0lb, mpre0ub),
               c(mpos0, mpos0lb, mpos0ub),
               c(mpre1, mpre1lb, mpre1ub),
               c(mpos1, mpos1lb, mpos1ub))),2)
```

```
> subgroup_pcv <- head(bootstrap_df, 1000)
>
> mpre0        = basemodel$coef[5]
> mpos0                = basemodel$coef[7]
> mpre1        = basemodel$coef[5] + basemodel$coef[21]
> mpos1                = basemodel$coef[7] + basemodel$coef[22]
> mpre0lb              = quantile ( subgroup_pcv[,5], 0.025)
> mpre0ub              = quantile ( subgroup_pcv[,5], 0.975)
> mpos0lb              = quantile ( subgroup_pcv[,7], 0.025)
> mpos0ub      = quantile ( subgroup_pcv[,7], 0.975)
>
> mpre1lb              = quantile ( subgroup_pcv[,5] + subgroup_pcv[,21],
0.025)
> mpre1ub              = quantile ( subgroup_pcv[,5] + subgroup_pcv[,21],
0.975)
> mpos1lb              = quantile ( subgroup_pcv[,7] + subgroup_pcv[,22],
0.025)
> mpos1ub      = quantile ( subgroup_pcv[,7] + subgroup_pcv[,22], 0.975)
>
> round(exp (
+ rbind (      c(mpre0, mpre0lb, mpre0ub),
+                      c(mpos0, mpos0lb, mpos0ub),
+                      c(mpre1, mpre1lb, mpre1ub),
+                      c(mpos1, mpos1lb, mpos1ub))),2)

                          RR   2.5% 97.5%
[1,]                     0.86 0.64  1.04
[2,]                     1.07 0.80  1.28
[3,]                     1.11 0.90  1.20
[4,]                     0.93 0.78  1.06
```

```
# SES

er$sesmedian=ifelse(er$ses >= quantile (
 aggregate(ses~hhid,data=er,FUN=mean)$ses, 0.5), 1, 0)
```

© 2025 McCracken JP et al. *JAMA Network Open*.

```
basemodel = gee (pneumonia ~
                              factor(quarter) +
                              I(dprenatalpm/predpmiqr)*sesmedian +
                              I(postnatalpm/postpmiqr)*sesmedian +
                              crowding +
                              birthweight +
                              winter +
                              covid +
                              ma0fe1 +
                              pcv +
                              irc,
                              data= er,
                              id=hhid,
                              family=binomial(link="log"),
                              corstr="exchangeable")



set.seed(125)  # For reproducibility
n_bootstrap <- 1050

# Pre-allocate a list to store coefficients from successful fits
results_list <- vector("list", n_bootstrap)

for (i in seq_len(n_bootstrap)) {
  if (i == 101) next   # Skip iteration 101

  # 1) Resample clusters with replacement
  boothhid <- sample(uhhid, size = length(uhhid), replace = TRUE)
  hhidtable <- data.frame(hhid = boothhid, newhhid = seq_along(boothhid))
  resampled_data <- merge(er, hhidtable, by = "hhid")

  # 2) Fit the logistic regression (glm) and catch errors or non-convergence
  smodel <- try(
    suppressWarnings(
      glm ( pneumonia ~
              factor(quarter) +
              I(dprenatalpm/predpmiqr)*sesmedian +
              I(postnatalpm/postpmiqr)*sesmedian +
              crowding +
              birthweight +
              winter +
              covid +
              ma0fe1 +
              pcv +
              irc,
            data= resampled_data,
            family=binomial(link="log"))
    ),
    silent = TRUE
  )
  if (inherits(smodel, "try-error") || !isTRUE(smodel$converged)) {
    print(paste(i,
                if (inherits(smodel, "try-error")) "glm_error" else
 smodel$converged,
                NA,
                Sys.time()))
```

© 2025 McCracken JP et al. *JAMA Network Open*.

```
    next
  }

  # 3) Fit the GEE model and catch errors or non-convergence
  model <- try(
    suppressWarnings(
      glmgee (pneumonia ~
                factor(quarter) +
                I(dprenatalpm/predpmiqr)*sesmedian +
                I(postnatalpm/postpmiqr)*sesmedian +
                crowding +
                birthweight +
                winter +
                covid +
                ma0fe1 +
                pcv +
                irc,
              data= resampled_data,
              id=newhhid,
              family=binomial(link="log"),
              corstr="exchangeable")
    ),
    silent = TRUE
  )
  if (inherits(model, "try-error") ||
      !is.logical(model$converged) ||
      !isTRUE(model$converged)
  ) {
    print(paste(i,
                smodel$converged,
                if (inherits(model, "try-error")) "gee_error" else
model$converged,
                Sys.time()))
    next
  }

  # 4) On success, print iteration info and save coefficients
  print(paste(i, smodel$converged, model$converged, Sys.time()))
  results_list[[i]] <- as.numeric(model$coef)
}

# Combine all successful coefficient vectors into one data frame
bootstrap_df <- do.call(rbind, results_list)

subgroup_ses <- head(bootstrap_df, 1000)

mpre0          = basemodel$coef[5]
mpos0          = basemodel$coef[7]
mpre1          = basemodel$coef[5] + basemodel$coef[19]
mpos1          = basemodel$coef[7] + basemodel$coef[20]
mpre0lb        = quantile ( subgroup_ses[,5], 0.025)
mpre0ub        = quantile ( subgroup_ses[,5], 0.975)
mpos0lb        = quantile ( subgroup_ses[,7], 0.025)
mpos0ub        = quantile ( subgroup_ses[,7], 0.975)

mpre1lb        = quantile ( subgroup_ses[,5] + subgroup_ses[,19], 0.025)
mpre1ub        = quantile ( subgroup_ses[,5] + subgroup_ses[,19], 0.975)
```

© 2025 McCracken JP et al. *JAMA Network Open*.

```
mpos1lb        = quantile ( subgroup_ses[,7] + subgroup_ses[,20], 0.025)
mpos1ub        = quantile ( subgroup_ses[,7] + subgroup_ses[,20], 0.975)

round(exp (
rbind (        c(mpre0, mpre0lb, mpre0ub),
               c(mpos0, mpos0lb, mpos0ub),
               c(mpre1, mpre1lb, mpre1ub),
               c(mpos1, mpos1lb, mpos1ub))),2)
```

```
> subgroup_ses <- head(bootstrap_df, 1000)
>
> mpre0        = basemodel$coef[5]
> mpos0        = basemodel$coef[7]
> mpre1        = basemodel$coef[5] + basemodel$coef[19]
> mpos1        = basemodel$coef[7] + basemodel$coef[20]
> mpre0lb      = quantile ( subgroup_ses[,5], 0.025)
> mpre0ub      = quantile ( subgroup_ses[,5], 0.975)
> mpos0lb      = quantile ( subgroup_ses[,7], 0.025)
> mpos0ub      = quantile ( subgroup_ses[,7], 0.975)
>
> mpre1lb      = quantile ( subgroup_ses[,5] + subgroup_ses[,19], 0.025)
> mpre1ub      = quantile ( subgroup_ses[,5] + subgroup_ses[,19], 0.975)
> mpos1lb      = quantile ( subgroup_ses[,7] + subgroup_ses[,20], 0.025)
> mpos1ub      = quantile ( subgroup_ses[,7] + subgroup_ses[,20], 0.975)
>
> round(exp (
+ rbind (      c(mpre0, mpre0lb, mpre0ub),
+                      c(mpos0, mpos0lb, mpos0ub),
+                      c(mpre1, mpre1lb, mpre1ub),
+                      c(mpos1, mpos1lb, mpos1ub))),2)

     I(dprenatalpm/predpmiqr) 2.5% 97.5%
[1,]                     1.06 0.92  1.16
[2,]                     0.96 0.82  1.08
[3,]                     0.75 0.43  1.01
[4,]                     1.02 0.67  1.20
```

```
## Andersen-Gill counting process
# For severe infant pneumonia

# Building the time-to-event (toe) dataset
# starting with the dataset obtained from the Data Management Core
# renamed er3_week_coxreg_20230728.sas7bdat to toe.sas7bdat

toe = read_sas("toe.sas7bdat")

# adding missing variables from er database
# m10sleep is number of people who sleep in the house
# ses is the socioeconomic wealth index
# sleep10 is 10+ people sleeping in house
# crowding is the m10sleep variable stratified into quartiles
# sescat is the ses variable stratified into quartiles

toe_conf  = aggregate ( m10_sleep ~ hhid, data=conf, FUN=unique)
toe_conf2 = aggregate ( ses ~ hhid, data=conf, FUN=unique)
```

© 2025 McCracken JP et al. *JAMA Network Open*.

```
toe_conf = merge(toe_conf, toe_conf2, by="hhid", all=T)
toe_conf$sleep10 = ifelse(toe_conf$m10_sleep>=10,1,0)
toe_conf$crowding = cut (toe_conf$m10_sleep,
                         breaks = quantile(toe_conf$m10_sleep,0:4/4, na.rm =
 TRUE),
                         include.lowest=T, right=T)
sesquart = quantile(toe_conf2$ses, c(0,0.25,0.5,0.75,1) )
toe_conf$sescat = cut (toe_conf$ses, breaks=sesquart, include.lowest=T)

# merge with additional variables
toe = merge ( toe[,-18], toe_conf, by="hhid")

# Subset original database
toe = subset(toe, select=c(hhid, irc, start, stop, HE3_pneumonia,
 HE3_IMCI_Severe, motherprenatalpm, motherprenatalco, cumexp, cumexpco,
 m10_sleep, sleep10, crowding, c30_sex, winter, COVID, Birthwt_by_protocol,
 ses, sescat, vaccine))

# rename variables
names(toe)=c("hhid","irc","start","stop","pneumonia","whosevere","dprenatalpm
 ","dprenatalco","postnatalpm","postnatalco","sleephouse","sleep10","crowding"
 ,"ma0fe1", "winter", "covid", "birthweight", "ses","sescat","pcv")

# sex variable coded 1 male 2 female
# recoding to 0 male 1 female
toe$ma0fe1=toe$ma0fe1-1

# Enumerate recurrent episodes with an ordinal number
enumtoe = toe[toe$pneumonia==1,]
zerotoe = toe[toe$pneumonia==0,]
enumtoe = as.data.frame(enumtoe %>%
               group_by(hhid) %>%
               mutate(enumpneumonia=row_number()))
zerotoe$enumpneumonia = 0
toe = rbind ( enumtoe, zerotoe)
toe = toe [ order(toe$hhid, toe$start),]


# Convert start and stop into R date variables
toe$start = as.Date(toe$start, format="%Y-%m-%d")
toe$stop = as.Date(toe$stop, format="%Y-%m-%d")

# Calculate start and stop in days
toe$tstart = toe$start - min(toe$start)
toe$tstop = toe$stop - min(toe$start)


# Data enumeration as a stratum
toe2=NULL
thhid = unique(toe$hhid)
for (i in 1:length(thhid))
       {
       toeid = toe [ toe$hhid == thhid[i],]
       enum = 1
       for (j in 1:dim(toeid)[1])
               {
               toeid$enum[j]=enum
```

© 2025 McCracken JP et al. *JAMA Network Open*.

```
                if (toeid$pneumonia[j]==1) enum=enum+1
                }
        toe2=rbind(toe2,toeid)
        print(i)
        }


# Remove enumpneumonia and just leave enum and the stratum enumerator
toe2 = subset(toe2, select=-c(enumpneumonia))


# Run A-G models for primary outcome (severe infant pneumonia)
# Uses the WLW method to include a robust variance estimator

model = coxph(Surv(tstart,tstop,pneumonia) ~
                       I(dprenatalpm/predpmiqr) +
                       I(postnatalpm/postpmiqr) +
                       ma0fe1 +
                       crowding +
                       sescat +
                       I(birthweight/1000) +
                       winter +
                       covid +
                       pcv +
                       irc+
                       strata(enum),
                       cluster=hhid,
                       data=toe2,
                       ties="breslow")
                       summary(model)

# Calculate HRs and 95% CI
x=summary(model)$coef
y=data.frame(RR=exp(x[,1]),LB95=exp(x[,1]-
 qnorm(0.975)*x[,4]),UB95=exp(x[,1]+ qnorm(0.975)*x[,4]))
round(y,4)
```

```
> model = coxph(Surv(tstart,tstop,pneumonia) ~
+                              I(dprenatalpm/predpmiqr) +
+                              I(postnatalpm/postpmiqr) +
+                              ma0fe1 +
+                              crowding +
+                              sescat +
+                              I(birthweight/1000) +
+                              winter +
+                              covid +
+                              pcv +
+                              irc+
+                              strata(enum),
+                              cluster=hhid,
+                              data=toe2,
+                              ties="breslow")
Warning message:
In agreg.fit(X, Y, istrat, offset, init, control, weights = weights,  :
  Loglik converged before variable  12 ; beta may be infinite.
>                              summary(model)
```

© 2025 McCracken JP et al. *JAMA Network Open*.

```
Call:
coxph(formula = Surv(tstart, tstop, pneumonia) ~ I(dprenatalpm/predpmiqr) +
    I(postnatalpm/postpmiqr) + ma0fe1 + crowding + sescat + I(birthweight/1000) +
    winter + covid + pcv + irc + strata(enum), data = toe2, ties = "breslow",
    cluster = hhid)

  n= 148745, number of events= 167
   (6314 observations deleted due to missingness)

                                  coef       exp(coef)        se(coef)
I(dprenatalpm/predpmiqr)    0.0553088160    1.0568669417    0.0627371001
I(postnatalpm/postpmiqr)   -0.0330652937    0.9674753875    0.0619984946
ma0fe1                     -0.0539430205    0.9474860922    0.1575631411
crowding(3,4]               0.0353641436    1.0359968918    0.2151414442
crowding(4,5]               0.3639332949    1.4389782238    0.2353015985
crowding(5,18]              0.0342458027    1.0348389417    0.2243574840
sescat(0.198,0.353]        -0.1354629942    0.8733114799    0.2094255283
sescat(0.353,0.529]        -0.4483752876    0.6386649560    0.2981772183
sescat(0.529,1]             0.2282751188    1.2564309456    0.4113129870
I(birthweight/1000)        -0.6349590503    0.5299571895    0.1915545725
winter                     -0.1859733061    0.8302957585    0.2782533494
covid                     -15.0274935141    0.0000002976 1539.2260157425
pcv                        -0.8069516924    0.4462161994    0.1742337798
ircIndia                   -2.7168758100    0.0660808815    0.4904399520
ircPeru                    -1.2267911506    0.2932320070    0.3551070645
ircRwanda                   0.2339205856    1.2635441446    0.2490523293
                             robust se        z             Pr(>|z|)
I(dprenatalpm/predpmiqr)    0.0430267088   1.285              0.19863
I(postnatalpm/postpmiqr)    0.0546339909  -0.605              0.54504
ma0fe1                      0.1653858727  -0.326              0.74430
crowding(3,4]               0.2226681362   0.159              0.87381
crowding(4,5]               0.2314544361   1.572              0.11586
crowding(5,18]              0.2237953996   0.153              0.87838
sescat(0.198,0.353]         0.2078835291  -0.652              0.51464
sescat(0.353,0.529]         0.3259379632  -1.376              0.16893
sescat(0.529,1]             0.3501283694   0.652              0.51442
I(birthweight/1000)         0.2276325152  -2.789              0.00528 **
winter                      0.3013413244  -0.617              0.53713
covid                       0.8434014942 -17.818 < 0.0000000000000002 ***
pcv                         0.1798836589  -4.486      0.0000072584857 ***
ircIndia                    0.4135118331  -6.570      0.0000000000502 ***
ircPeru                     0.3771358690  -3.253              0.00114 **
ircRwanda                   0.2693658977   0.868              0.38517
---
Signif. codes:  0 '***' 0.001 '**' 0.01 '*' 0.05 '.' 0.1 ' ' 1

                          exp(coef)    exp(-coef)     lower .95    upper
.95
I(dprenatalpm/predpmiqr) 1.0568669417       0.9462 0.97139510937
1.149859333
I(postnatalpm/postpmiqr) 0.9674753875       1.0336 0.86923136332
1.076823346
ma0fe1                   0.9474860922       1.0554 0.68516652054
1.310236078
crowding(3,4]            1.0359968918       0.9653 0.66961163509
1.602853809
```

© 2025 McCracken JP et al. *JAMA Network Open*.

```
crowding(4,5]             1.4389782238       0.6949 0.91419711954
2.265002027
crowding(5,18]            1.0348389417       0.9663 0.66738704739
1.604603565
sescat(0.198,0.353]       0.8733114799       1.1451 0.58105653281
1.312562372
sescat(0.353,0.529]       0.6386649560       1.5658 0.33715855942
1.209795554
sescat(0.529,1]           1.2564309456       0.7959 0.63257032724
2.495562395
I(birthweight/1000)       0.5299571895       1.8869 0.33921858643
0.827945855
winter                    0.8302957585       1.2044 0.45997117756
1.498770097
covid                     0.0000002976 3360141.0630 0.00000005698
0.000001554
pcv                       0.4462161994       2.2411 0.31363757318
0.634837512
ircIndia                  0.0660808815      15.1330 0.02938284995
0.148613321
ircPeru                   0.2932320070       3.4103 0.14002085950
0.614087146
ircRwanda                 1.2635441446       0.7914 0.74525646427
2.142274347

Concordance= 0.765  (se = 0.018 )
Likelihood ratio test= 118.2  on 16 df,   p=<0.0000000000000002
Wald test            = 394  on 16 df,   p=<0.0000000000000002
Score (logrank) test = 113.1  on 16 df,   p=<0.0000000000000002,   Robust =
94.68  p=0.0000000000003

  (Note: the likelihood ratio and score tests assume independence of
     observations within a cluster, the Wald and robust score tests do
not).
>
> # Calculate HRs and 95% CI
> x=summary(model)$coef
> y=data.frame(RR=exp(x[,1]),LB95=exp(x[,1]-
qnorm(0.975)*x[,4]),UB95=exp(x[,1]+ qnorm(0.975)*x[,4]))
> round(y,4)
                              RR   LB95   UB95
I(dprenatalpm/predpmiqr) 1.0569 0.9714 1.1499
I(postnatalpm/postpmiqr) 0.9675 0.8692 1.0768
ma0fe1                   0.9475 0.6852 1.3102
crowding(3,4]            1.0360 0.6696 1.6029
crowding(4,5]            1.4390 0.9142 2.2650
crowding(5,18]           1.0348 0.6674 1.6046
sescat(0.198,0.353]      0.8733 0.5811 1.3126
sescat(0.353,0.529]      0.6387 0.3372 1.2098
sescat(0.529,1]          1.2564 0.6326 2.4956
I(birthweight/1000)      0.5300 0.3392 0.8279
winter                   0.8303 0.4600 1.4988
covid                    0.0000 0.0000 0.0000
pcv                      0.4462 0.3136 0.6348
ircIndia                 0.0661 0.0294 0.1486
ircPeru                  0.2932 0.1400 0.6141
ircRwanda                1.2635 0.7453 2.1423
```

© 2025 McCracken JP et al. *JAMA Network Open*.

```
# Main model without birthweight

model = gee (pneumonia ~
                      factor(quarter) +
                      I(dprenatalpm/predpmiqr) +
                      I(postnatalpm/postpmiqr) +
                      crowding +
                      ma0fe1 +
                      winter +
                      covid +
                      sescat +
                      pcv +
                      irc,
                      data= er,
                      id=hhid,
                      family=binomial(link="log"),
                      corstr="exchangeable")

# Calculate RR and 95% CI
x=round(summary(model)$coef,4)
round(x,4)
y=data.frame(  RR=exp(x[,1]),
               LB95=exp(x[,1]- qnorm(0.975)*x[,4]),
               UB95=exp(x[,1]+ qnorm(0.975)*x[,4]))
round(y,2)
```

```
> model = gee (pneumonia ~
+                             factor(quarter) +
+                             I(dprenatalpm/predpmiqr) +
+                             I(postnatalpm/postpmiqr) +
+                             crowding +
+                             ma0fe1 +
+                             winter +
+                             covid +
+                             sescat +
+                             pcv +
+                             irc,
+                             data= er,
+                             id=hhid,
+                             family=binomial(link="log"),
+                             corstr="exchangeable")
Beginning Cgee S-function, @(#) geeformula.q 4.13 98/01/27
running glm to get initial regression estimate
             (Intercept)        factor(quarter)2        factor(quarter)3
            -2.961964695            -0.996782388            -1.122532729
        factor(quarter)4 I(dprenatalpm/predpmiqr) I(postnatalpm/postpmiqr)
            -0.831086621             0.060763778            -0.040549692
           crowding(3,4]           crowding(4,5]          crowding(5,18]
            -0.062512993             0.234689530             0.078100661
                  ma0fe1                  winter                   covid
            -0.001271906             0.621252726            -0.222480330
     sescat(0.198,0.353]     sescat(0.353,0.529]       sescat(0.529,1]
            -0.153201943            -0.582682601             0.070912120
```

© 2025 McCracken JP et al. *JAMA Network Open*.

```
                        pcv                      ircIndia                      ircPeru
               -0.553395069                  -2.477764283                 -1.513788652
                  ircRwanda
                0.507224917
>
> # Calculate RR and 95% CI
> x=round(summary(model)$coef,4)

> round(x,4)
                         Estimate Naive S.E. Naive z Robust S.E. Robust z
(Intercept)               -2.9365     0.3302 -8.8926      0.3648  -8.0491
factor(quarter)2          -0.9931     0.2066 -4.8060      0.2208  -4.4971
factor(quarter)3          -1.1220     0.2248 -4.9917      0.2564  -4.3764
factor(quarter)4          -0.8361     0.2163 -3.8649      0.2534  -3.2990
I(dprenatalpm/predpmiqr)   0.0595     0.0635  0.9365      0.0600   0.9920
I(postnatalpm/postpmiqr)  -0.0432     0.0595 -0.7255      0.0610  -0.7078
crowding(3,4]             -0.0526     0.2062 -0.2552      0.2232  -0.2358
crowding(4,5]              0.2337     0.2232  1.0473      0.2329   1.0036
crowding(5,18]             0.0947     0.2094  0.4523      0.2268   0.4177
ma0fe1                    -0.0065     0.1493 -0.0437      0.1609  -0.0406
winter                     0.6187     0.2216  2.7922      0.2462   2.5127
covid                     -0.1997     0.1664 -1.2004      0.2047  -0.9757
sescat(0.198,0.353]       -0.1626     0.1976 -0.8229      0.2104  -0.7731
sescat(0.353,0.529]       -0.6043     0.2839 -2.1284      0.3254  -1.8573
sescat(0.529,1]            0.0571     0.3872  0.1473      0.3504   0.1628
pcv                       -0.5721     0.1949 -2.9354      0.2114  -2.7056
ircIndia                  -2.4709     0.4789 -5.1599      0.4498  -5.4939
ircPeru                   -1.5103     0.3227 -4.6807      0.3180  -4.7486
ircRwanda                  0.4912     0.2552  1.9245      0.3022   1.6250

> y=data.frame(        RR=exp(x[,1]),
+                      LB95=exp(x[,1]- qnorm(0.975)*x[,4]),
+                      UB95=exp(x[,1]+ qnorm(0.975)*x[,4]))
> round(y,2)
                           RR LB95 UB95
(Intercept)              0.05 0.03 0.11
factor(quarter)2         0.37 0.24 0.57
factor(quarter)3         0.33 0.20 0.54
factor(quarter)4         0.43 0.26 0.71
I(dprenatalpm/predpmiqr) 1.06 0.94 1.19
I(postnatalpm/postpmiqr) 0.96 0.85 1.08
crowding(3,4]            0.95 0.61 1.47
crowding(4,5]            1.26 0.80 1.99
crowding(5,18]           1.10 0.70 1.71
ma0fe1                   0.99 0.72 1.36
winter                   1.86 1.15 3.01
covid                    0.82 0.55 1.22
sescat(0.198,0.353]      0.85 0.56 1.28
sescat(0.353,0.529]      0.55 0.29 1.03
sescat(0.529,1]          1.06 0.53 2.10
pcv                      0.56 0.37 0.85
ircIndia                 0.08 0.03 0.20
ircPeru                  0.22 0.12 0.41
ircRwanda                1.63 0.90 2.96
```

© 2025 McCracken JP et al. *JAMA Network Open*.

```
## Table 1
##
conf2 = aggregate ( momage ~ hhid, data=hapinxtra, FUN=max)
conf3 = conf2
conf2 = aggregate ( momht ~ hhid, data=hapinxtra, FUN=max)
conf3 = merge(conf3, conf2,by="hhid", all=T)
conf2 = aggregate ( garandom ~ hhid, data=hapinxtra, FUN=max)
conf3 = merge(conf3, conf2,by="hhid", all=T)
fies = aggregate ( fies ~ hhid, data=conf, FUN=max)
conf3 = merge(conf3, fies, by="hhid", all=T)

zhap6=merge(er, conf3, by="hhid", all=T)
zhap6 = subset ( zhap6, hhid %in% uhhid )

zhap7 = subset(zhap6, select=c(hhid, dprenatalpm, dprenatalpmcat, momage,
 momht, garandom, ma0fe1, sleephouse, fies, ses))

# collapse into 1 row per child for prenatal
zhap7 = unique(zhap7)

# sample size
table(zhap7$dprenatalpmcat)
table(zhap6$postnatalpmcat, zhap6$quarter)

# momage
round(aggregate ( momage ~ dprenatalpmcat, data = zhap7, FUN=mean)$momage,1)
round(aggregate ( momage ~ dprenatalpmcat, data = zhap7, FUN=sd)$momage,1)

round(aggregate ( momage ~ postnatalpmcat + quarter, data = zhap6,
 FUN=mean)$momage,1)
round(aggregate ( momage ~ postnatalpmcat + quarter, data = zhap6,
 FUN=sd)$momage,1)

# momht
round(aggregate ( momht ~ dprenatalpmcat, data = zhap7, FUN=mean)$momht,1)
round(aggregate ( momht ~ dprenatalpmcat, data = zhap7, FUN=sd)$momht,1)

round(aggregate ( momht ~ postnatalpmcat + quarter, data = zhap6,
 FUN=mean)$momht,1)
round(aggregate ( momht ~ postnatalpmcat + quarter, data = zhap6,
 FUN=sd)$momht,1)

# garandom
round(aggregate ( garandom ~ dprenatalpmcat, data = zhap7,
 FUN=mean)$garandom,1)
round(aggregate ( garandom ~ dprenatalpmcat, data = zhap7,
 FUN=sd)$garandom,1)

round(aggregate ( garandom ~ postnatalpmcat + quarter, data = zhap6,
 FUN=mean)$garandom,1)
round(aggregate ( garandom ~ postnatalpmcat + quarter, data = zhap6,
 FUN=sd)$garandom,1)

# sex
aggregate ( ma0fe1 ~ dprenatalpmcat, data = zhap7, FUN=sum)
```

© 2025 McCracken JP et al. *JAMA Network Open*.

```
aggregate ( ma0fe1 ~ postnatalpmcat + quarter, data = zhap6, FUN=sum)

# sleep
q25=function(x){quantile(x,0.25,na.rm=T)}
q75=function(x){quantile(x,0.75,na.rm=T)}

aggregate ( sleephouse ~ dprenatalpmcat, data = zhap7, FUN=median)
aggregate ( sleephouse ~ dprenatalpmcat, data = zhap7, FUN=q25)
aggregate ( sleephouse ~ dprenatalpmcat, data = zhap7, FUN=q75)

aggregate ( sleephouse ~ postnatalpmcat + quarter, data = zhap6, FUN=median)
aggregate ( sleephouse ~ postnatalpmcat + quarter, data = zhap6, FUN=q25)
aggregate ( sleephouse ~ postnatalpmcat + quarter, data = zhap6, FUN=q75)

# fies
round(aggregate ( fies ~ dprenatalpmcat, data = zhap7, FUN=mean)$fies,1)
round(aggregate ( fies ~ dprenatalpmcat, data = zhap7, FUN=sd)$fies,1)

round(aggregate ( fies ~ postnatalpmcat + quarter, data = zhap6,
 FUN=mean)$fies,1)
round(aggregate ( fies ~ postnatalpmcat + quarter, data = zhap6,
 FUN=sd)$fies,1)

# ses
round(aggregate ( ses ~ dprenatalpmcat, data = zhap7, FUN=mean)$ses,2)
round(aggregate ( ses ~ dprenatalpmcat, data = zhap7, FUN=sd)$ses,2)

round(aggregate ( ses ~ postnatalpmcat + quarter, data = zhap6,
 FUN=mean)$ses,2)
round(aggregate ( ses ~ postnatalpmcat + quarter, data = zhap6,
 FUN=sd)$ses,2)



## Review of numbers in the paper
#

# The 3,061 infants had 13,910 (75·7%) prenatal and post-natal personal
 exposures to PM2·5 out of a possible 18,366 assessments.
sum(is.na(hap2$echildpm)==F)
#[1] 13910
3061*6
#[1] 18366

#While there were 3,892 (54·7%) missing infant exposures to PM2·5 out of
 7,110 attempted assessments during the post-natal period; this number was
 reduced to 873 (12.3%)
sum(is.na(hap2$childpm[hap2$visit=="B1" | hap2$visit=="B2" |
 hap2$visit=="B4"]))
#[1] 3892

length(hap2$childpm[hap2$visit=="B1" | hap2$visit=="B2" | hap2$visit=="B4"])
# [1] 7110

sum(is.na(hap2$echildpm[hap2$visit=="B1" | hap2$visit=="B2" |
 hap2$visit=="B4"]))
#[1] 873
```

© 2025 McCracken JP et al. *JAMA Network Open*.

```
#Spearman correlation between personal exposures to PM2·5 obtained from the
 mother and the infant personal exposure in the same visit was 0.89 (95% CI
 0.88 to 0.90).
spearman.ci(hap2$mompm, hap2$childpm)
#
#       Spearman's rank correlation
#data:  hap2$mompm and hap2$childpm
#1000 replicates
#
#95 percent confidence interval:
# 0.877471 0.901961
#sample estimates:
#      rho
#0.8908717


#A total of 3025 (98.8%) infants out of the 3,061 had prenatal exposures to
 PM2.5
whap4 = subset(whap3, hhid %in% uhhid)
sum(is.na(whap4$dprenatalpm)==F)
#[1] 3025

#with a mean (± SD) of 88.6 ± 81.1 µg/m3 and ranging between 10.7 and 1090
 µg/m3;
round(mean(whap4$dprenatalpm,na.rm=T),1)
#[1] 88.6
round(sd(whap4$dprenatalpm,na.rm=T),1)
#[1] 81.1
round(min(whap4$dprenatalpm,na.rm=T),1)
#[1] 10.7
round(max(whap4$dprenatalpm,na.rm=T),1)
#[1] 1089.8

# 2879 (94%) infants had post-natal personal exposures to PM2.5 (B1-B4
 visits) with a mean of 67.5 ± 92.1 µg/m3 and ranging between 5.4 and 1182
 µg/m3
dim(hap6)
#[1] 2879    2
round(mean(hap6$postnatalpm,na.rm=T),1)
#[1] 67.5
round(sd(hap6$postnatalpm,na.rm=T),1)
#[1] 92.1
round(min(hap6$postnatalpm,na.rm=T),1)
#[1] 5.4
round(max(hap6$postnatalpm,na.rm=T),1)
#[1] 1181.9

# A total of 3037 (99.2%) infants had a prenatal exposure to CO with a mean
 of 1.9 ± 2.9 ppm and ranging from 0 to 46.4 ppm
sum(is.na(whap4$dprenatalco)==F)
# [1] 3037
round(mean(whap4$dprenatalco,na.rm=T),1)
#[1] 1.9
round(sd(whap4$dprenatalco,na.rm=T),1)
#[1] 2.9
round(min(whap4$dprenatalco,na.rm=T),1)
```

© 2025 McCracken JP et al. *JAMA Network Open*.

```
#[1] 0
round(max(whap4$dprenatalco,na.rm=T),1)
#[1] 46.4

# 2822 (92.2%) infants had a post-natal exposure to CO with a mean 1.7 ± 3.5
 ppm and ranging from 0 to 70 ppm.
hap8 = aggregate ( echildco ~ hhid, data = hap5, FUN=mean)
names(hap8)[2]="postnatalco"
dim(hap8)
round(mean(hap8$postnatalco,na.rm=T),1)
round(sd(hap8$postnatalco,na.rm=T),1)
round(min(hap8$postnatalco,na.rm=T),1)
round(max(hap8$postnatalco,na.rm=T),1)

# Infants with at least one episode of severe infant pneumonia had similar
 prenatal (101[100] vs. 88[80] µg/m3, respectively, for a difference of 13
 µg/m3, 95% CI -3 to 29 µg/m3; p=0.11) and post-natal exposures to PM2.5
 (70[787] vs. 67[93] µg/m3, respectively, for a difference of 2 µg/m3, 95% CI
 -11 to 17 µg/m3; p=0.68) than did infants without severe pneumonia.

y=aggregate (pneumonia ~ hhid, data=er, FUN=max)
# Data.frame whap4 contains mean prenatal PM
y=merge(y,whap4[,c(1,17)],by="hhid",all=T)
# Data.frame hap6 contains mean postnatal PM
y=merge(y,hap6,by="hhid",all=T)

t.test(y$dprenatalpm[y$pneumonia==1], y$dprenatalpm[y$pneumonia==0])
mean(y$dprenatalpm[y$pneumonia==1],na.rm=T) -
 mean(y$dprenatalpm[y$pneumonia==0], na.rm=T)

mean(y$dprenatalpm[y$pneumonia==1],na.rm=T)
sd(y$dprenatalpm[y$pneumonia==1],na.rm=T)
mean(y$dprenatalpm[y$pneumonia==0], na.rm=T)
sd(y$dprenatalpm[y$pneumonia==0], na.rm=T)

t.test(y$postnatalpm[y$pneumonia==1], y$postnatalpm[y$pneumonia==0])
mean(y$postnatalpm[y$pneumonia==1],na.rm=T) -
 mean(y$postnatalpm[y$pneumonia==0], na.rm=T)

mean(y$postnatalpm[y$pneumonia==1],na.rm=T)
sd(y$postnatalpm[y$pneumonia==1],na.rm=T)
mean(y$postnatalpm[y$pneumonia==0], na.rm=T)
sd(y$postnatalpm[y$pneumonia==0], na.rm=T)

# Infants with at least one episode of severe IMCI infant pneumonia had
 higher prenatal (104 vs. 88 µg/m3; p=0.03) and similar post-natal exposures
 (71 vs. 67 µg/m3; p=0.59) to PM2.5 when compared to the exposures in infants
 without severe IMCI infant pneumonia.

y=aggregate (whosevere ~ hhid, data=er, FUN=max)
# Data.frame whap4 contains mean prenatal PM
y=merge(y,whap4[,c(1,17)],by="hhid",all=T)
# Data.frame hap6 contains mean postnatal PM
y=merge(y,hap6,by="hhid",all=T)

t.test(y$dprenatalpm[y$whosevere==1], y$dprenatalpm[y$whosevere==0])
```

© 2025 McCracken JP et al. *JAMA Network Open*.

```
mean(y$dprenatalpm[y$whosevere==1],na.rm=T) -
 mean(y$dprenatalpm[y$whosevere==0], na.rm=T)

mean(y$dprenatalpm[y$whosevere==1],na.rm=T)
sd(y$dprenatalpm[y$whosevere==1], na.rm = TRUE)
mean(y$dprenatalpm[y$whosevere==0], na.rm=T)
sd(y$dprenatalpm[y$whosevere==0], na.rm = TRUE)

t.test(y$postnatalpm[y$whosevere==1], y$postnatalpm[y$whosevere==0])
mean(y$postnatalpm[y$whosevere==1],na.rm=T) -
 mean(y$postnatalpm[y$whosevere==0], na.rm=T)

mean(y$postnatalpm[y$whosevere==1],na.rm=T)
sd(y$postnatalpm[y$whosevere==1], na.rm = TRUE)
mean(y$postnatalpm[y$whosevere==0], na.rm=T)
sd(y$postnatalpm[y$whosevere==0], na.rm = TRUE)

# Main model without birthweight
model = gee (pneumonia ~
                      factor(quarter) +
                      I(dprenatalpm/predpmiqr) +
                      I(postnatalpm/postpmiqr) +
                      crowding +
                      ma0fe1 +
                      winter +
                      covid +
                      sescat +
                      pcv +
                      irc,
                      data= er,
                      id=hhid,
                      family=binomial(link="log"),
                      corstr="exchangeable")

# Calculate RR and 95% CI
x=round(summary(model)$coef,4)
round(x,4)
y=data.frame(  RR=exp(x[,1]),
               LB95=exp(x[,1]- qnorm(0.975)*x[,4]),
               UB95=exp(x[,1]+ qnorm(0.975)*x[,4]))
round(y,2)


## Figures
## Visualizations co-authored with Mingling Yang

# Figure 1
# df is database for figure
df=er

# Custom function for split violin plot
GeomSplitViolin <- ggproto("GeomSplitViolin", GeomViolin,
                           draw_group = function(self, data, ...,
 draw_quantiles = NULL) {
                     # Original function by Jan Gleixner (@jan-glx)
                     # Adjustments by Wouter van der Bijl (@Axeman)
```

© 2025 McCracken JP et al. *JAMA Network Open*.

```
                              data <- transform(data, xminv = x - violinwidth
 * (x - xmin), xmaxv = x + violinwidth * (xmax - x))
                              grp <- data[1, "group"]
                              newdata <- plyr::arrange(transform(data, x = if
 (grp %% 2 == 1) xminv else xmaxv), if (grp %% 2 == 1) y else -y)
                              newdata <- rbind(newdata[1, ], newdata,
 newdata[nrow(newdata), ], newdata[1, ])
                              newdata[c(1, nrow(newdata) - 1, nrow(newdata)),
 "x"] <- round(newdata[1, "x"])
                              if (length(draw_quantiles) > 0 &
 !scales::zero_range(range(data$y))) {
                                stopifnot(all(draw_quantiles >= 0),
 all(draw_quantiles <= 1))
                                quantiles <-
 create_quantile_segment_frame(data, draw_quantiles, split = TRUE, grp = grp)
                                aesthetics <- data[rep(1, nrow(quantiles)),
 setdiff(names(data), c("x", "y")), drop = FALSE]
                                aesthetics$alpha <- rep(1, nrow(quantiles))
                                both <- cbind(quantiles, aesthetics)
                                quantile_grob <- GeomPath$draw_panel(both,
 ...)
                                ggplot2::ggname("geom_split_violin",
 grid::grobTree(GeomPolygon$draw_panel(newdata, ...), quantile_grob))
                              }
                              else {
                                ggplot2:::ggname("geom_split_violin",
 GeomPolygon$draw_panel(newdata, ...))
                              }
                            }
)

create_quantile_segment_frame <- function(data, draw_quantiles, split =
 FALSE, grp = NULL) {
  dens <- cumsum(data$density) / sum(data$density)
  ecdf <- stats::approxfun(dens, data$y)
  ys <- ecdf(draw_quantiles)
  violin.xminvs <- (stats::approxfun(data$y, data$xminv))(ys)
  violin.xmaxvs <- (stats::approxfun(data$y, data$xmaxv))(ys)
  violin.xs <- (stats::approxfun(data$y, data$x))(ys)
  if (grp %% 2 == 0) {
    data.frame(
      x = ggplot2:::interleave(violin.xs, violin.xmaxvs),
      y = rep(ys, each = 2), group = rep(ys, each = 2)
    )
  } else {
    data.frame(
      x = ggplot2:::interleave(violin.xminvs, violin.xs),
      y = rep(ys, each = 2), group = rep(ys, each = 2)
    )
  }
}

geom_split_violin <- function(mapping = NULL, data = NULL, stat = "ydensity",
 position = "identity", ...,
                              draw_quantiles = NULL, trim = TRUE, scale =
 "area", na.rm = FALSE,
                              show.legend = NA, inherit.aes = TRUE) {
```

© 2025 McCracken JP et al. *JAMA Network Open*.

```
  layer(data = data, mapping = mapping, stat = stat, geom = GeomSplitViolin,
 position = position,
        show.legend = show.legend, inherit.aes = inherit.aes,
        params = list(trim = trim, scale = scale, draw_quantiles =
 draw_quantiles, na.rm = na.rm, ...))
}

# Set common y-axis limits
y_limits <- c(3, 3000)

# Create prenatal PM2.5 violin plot
prenatal_plot <- ggplot(df, aes(x = factor(quarter), y = dprenatalpm, fill =
 factor(pneumonia))) +
  geom_split_violin(trim = FALSE, color = "black", adjust = 2, draw_quantiles
 = c(0.25, 0.5, 0.75)) +
  scale_fill_manual(values = c("0" = "lightblue", "1" = "#fca27e"),
                    labels = c("No", "Yes"),
                    name = "Pneumonia") +
  scale_y_continuous(trans = scales::log_trans(base = exp(1)), # Apply
 natural log transformation
                     breaks = c(10, 25, 50, 100, 200, 500, 1000, 2500),
                     labels =  function(x) round(x, 0), # Use rounded actual
 values for labels
                     limits = y_limits) +  # Set common y-axis limits
  labs(y = expression("Prenatal PM"[2.5]~"(μg/m"^3*")")) +
  theme_bw() +
  theme(panel.border = element_blank(),
        panel.grid.major = element_blank(),
        panel.grid.minor = element_blank(),
        legend.position = "none",
        axis.text = element_text(size = 12),
        axis.text.x = element_blank(),
        axis.title.x = element_blank(),
        axis.title.y = element_text(size = 14, margin = margin(t = 15)),
        axis.ticks.x = element_blank(),
        axis.line.y = element_line(color = "black", size = 0.2))

# Create postnatal PM2.5 violin plot
postnatal_plot <- ggplot(df, aes(x = factor(quarter), y = postnatalpm, fill =
 factor(pneumonia))) +
  geom_split_violin(trim = FALSE, color = "black", adjust = 2, draw_quantiles
 = c(0.25, 0.5, 0.75)) +
  scale_fill_manual(values = c("0" = "lightblue", "1" = "#fca27e"),
                    labels = c("No", "Yes"),
                    name = "Pneumonia") +
  scale_y_continuous(trans = scales::log_trans(base = exp(1)), # Apply
 natural log transformation
                     breaks = c(10, 25, 50, 100, 200, 500, 1000, 2500),
                     labels =  function(x) round(x, 0), # Use rounded actual
 values for labels
                     limits = y_limits) +  # Set common y-axis limits
  labs(y = expression("Post-natal PM"[2.5]~"(μg/m"^3*")")) +
  theme_bw() +
  theme(panel.border = element_blank(),
        panel.grid.major = element_blank(),
        panel.grid.minor = element_blank(),
        legend.position = "none",
```

© 2025 McCracken JP et al. *JAMA Network Open*.

```
        axis.text = element_text(size = 12),
        axis.text.x = element_blank(),
        axis.title.x = element_blank(),
        axis.title.y = element_text(size = 14, margin = margin(t = 15)),
        axis.ticks.x = element_blank(),
        axis.line.y = element_line(color = "black", size = 0.2))

# Create a custom axis label grob
custom_x_labels <- textGrob(
  label = c("Pneumonia", "No", "Yes", "", "No", "Yes", "", "No", "Yes", "",
 "No", "Yes"),
  x = unit(c(0.068, 0.205, 0.25, 0.29, 0.417, 0.462, 0.53, 0.63, 0.674,
 0.783, 0.841, 0.885), "npc"),
  y = unit(0.6, "npc"),
  just = "center",
  gp = gpar(fontsize = 12)
)

custom_quarter_labels <- textGrob(
  label = c("Quarter", "1", "", "", "2", "", "", "3", "", "", "4", ""),
  x = unit(c(0.05, 0.226, 0.21, 0.29, 0.437, 0.45, 0.53, 0.650, 0.69, 0.77,
 0.861, 0.933), "npc"),
  y = unit(0.6, "npc"),
  just = "center",
  gp = gpar(fontsize = 12)
)

# Arrange plots with custom labels
Figure1 <- grid.arrange(
  prenatal_plot,
  custom_x_labels,
  custom_quarter_labels,
  postnatal_plot,
  ncol = 1,
  heights = c(1, 0.1, 0.1, 1)
)

ggsave("./Figure1.tiff", plot = figure1, device = "tiff", width = 8, height =
 7, dpi = 600)


# Figure S1

# df is database for figure
df <- er
df1 <- xhap2

# Load necessary libraries
library(ggplot2)
library(gridExtra) # For arranging multiple plots
library(grid) # For creating text labels in plots
library(tidyr)
library(patchwork) # For combining ggplots

x=subset(df1,select=c("blpm","p1pm","p2pm","b1pm","b2pm","b4pm"))
names(x)=c("BL","P1","P2","B1","B2","B4")
```

© 2025 McCracken JP et al. *JAMA Network Open*.

```
# Save the plot as TIFF
tiff("FigureS1.tiff", width = 8, height = 7, units = "in", res = 600)
corrplot(cor(x,use="pairwise.complete.obs",method="spearman"),type="upper",me
thod="ellipse",tl.srt=0,tl.offset=1,diag=F,addCoef.col="black")
dev.off()

# Figure S2
# df is database for figure
df=er

options(scipen = 999)
set.seed(1234567)
df2 <- df %>%
  mutate(
    predecile = cut(dprenatalpm, breaks = quantile(dprenatalpm, probs = 0:10
/ 10, na.rm = TRUE), include.lowest = TRUE,
                        dig.lab = 4),
    postdecile = cut(postnatalpm, breaks = quantile(postnatalpm, probs = 0:10
/ 10, na.rm = TRUE), include.lowest = TRUE,
                         dig.lab = 4)
  )

# Create tables for pneumonia status by deciles
pre_table <- table(df2$predecile, df2$pneumonia)
post_table <- table(df2$postdecile, df2$pneumonia)

# Convert to dataframes for ggplot
pre_df <- as.data.frame(pre_table) %>%
  group_by(Var1) %>%
  summarise(
    Percentage = round(100 * 4 * Freq[Var2 == 1] / (Freq[Var2 == 1] +
Freq[Var2 == 0]), 6)
  )

post_df <- as.data.frame(post_table) %>%
  group_by(Var1) %>%
  summarise(
    Percentage = round(100 * 4 * Freq[Var2 == 1] / (Freq[Var2 == 1] +
Freq[Var2 == 0]), 6)
  )

# Combine prenatal and postnatal data for faceting
combined_df <- bind_rows(
  pre_df %>% mutate(Phase = "Prenatal"),
  post_df %>% mutate(Phase = "Post-natal")
) %>%
  mutate(Phase = factor(Phase, levels = c("Prenatal", "Post-natal")))

# Create ggplot with faceting
facet_plot <- ggplot(combined_df, aes(x = Percentage, y = Var1)) +
  geom_point(size = 3) +
  facet_wrap(~ Phase, scales = "free", ncol = 2) +
  labs(
    x = "Risk of severe pneumonia (episodes per 100 infant-years)",
    y = expression("Personal exposure to PM"[2.5]*" (\u00b5g/m"^3*")")
  )  +
```

```
  scale_x_continuous(breaks = seq(0, 10, 2), labels = seq(0, 10, 2), limits =
 c(0, 10)) +  # Fixed x-axis limits
  theme_linedraw() + # Set plot theme
  theme(plot.title = element_text(size = 16, hjust = 0.5, vjust = 2),
        panel.grid.major = element_blank(),  # Remove major grid lines
        panel.grid.minor = element_blank(),
        axis.ticks.length = unit(5, "pt"),
        axis.minor.ticks.length = rel(0.5),
        axis.title.x = element_text(margin = margin(t = 15)),
        axis.title = element_text(size = 14),
        strip.text = element_text(size = 14, face = "bold", color = "black"),
 # Facet title as white
        strip.background = element_rect(fill = "white"),  # Background color
 for facet titles
        aspect.ratio = 1)

# Create ggplot with swapped axes and faceting
facet_plot <- ggplot(combined_df, aes(x = Var1, y = Percentage)) +
  geom_point(size = 3) +
  facet_wrap(~ Phase, scales = "free", ncol = 2) +
  labs(
    x = expression("Personal exposure to PM"[2.5]*" (\u00b5g/m"^3*")"),
    y = "Risk of severe pneumonia (episodes per 100 infant-years)"
  ) +
  scale_y_continuous(breaks = seq(0, 10, 2), labels = seq(0, 10, 2), limits =
 c(0, 10)) +  # Fixed y-axis limits
  theme_linedraw() +  # Set plot theme
  theme(
    plot.title = element_text(size = 16, hjust = 0.5, vjust = 2),
    panel.grid.major = element_blank(),  # Remove major grid lines
    panel.grid.minor = element_blank(),
    axis.ticks.length = unit(5, "pt"),
    axis.minor.ticks.length = rel(0.5),
    axis.title.x = element_text(margin = margin(t = 15)),
    axis.title = element_text(size = 14),
    axis.text.x = element_text(angle = 45, hjust = 1),  # Rotate x-axis
 labels
    strip.text = element_text(size = 14, face = "bold", color = "black"),  #
 Facet title as white
    strip.background = element_rect(fill = "white"),  # Background color for
 facet titles
    aspect.ratio = 1
  )

# Save the plot
ggsave("figureS2.tiff", plot = facet_plot, width = 12, height = 6, dpi = 600)
```

© 2025 McCracken JP et al. *JAMA Network Open*.
