# Supplementary material for: Exposure to Household Air Pollution From Biomass Cooking and Severe Pneumonia in Infants
Source: JAMA Netw Open. 2025 Oct 29;8(10):e2538721. doi: 10.1001/jamanetworkopen.2025.38721 (PMC12573034; doi:10.1001/jamanetworkopen.2025.38721)
Supplement: Supplement 2. — Nonauthor Collaborators [file jamanetwopen-e2538721-s002.pdf]

Supplemental Online Content: Nonauthor Collaborators
*First name, last name, and suffix (if applicable) are required and will appear in PubMed.

*Group Name(s): Household Air Pollution Intervention Network (HAPIN)							
***First Name and Middle Initial(s)**	***Last Name**	***Suffix (eg, Jr, III)**	Academic Degrees	Institution	Location (city, state/province, country)	Role or Contribution, eg, chair, principal investigator	Group (if more than 1 Group listed in the byline) and/or Subgroup (eg, Steering Committee)
Dana B	Barr		PhD	Emory University	Atlanta, GA, US	Lead Investigator - Biomarker Core	
Vanessa	Burrowes		PhD, MSPH	Johns Hopkins University	Baltimore, MD, US	Doctoral student	
Alejandra	Bussalleu		MSc	Asociación Benéfica PRISMA; Universidad Peruana Cayetano Heredia	Lima, Peru	Peru Site study manager	
Devan	Campbell		PhD, MPH	University of Georgia	Athens, GA, US	Doctoral student	
Eduardo	Canuz		BS	Universidad del Valle de Guatemala; Asociación Benéfica PRISMA	Guatemala City, Guatemala; Lima, Peru	Field staff; doctoral student	
Yunyun	Chen		MSPH	Emory University	Atlanta, GA, US	Statistician	
Maggie L	Clark		PhD	Colorado State University	Fort Collins, CO, US	Investigator - Biomarker Core	
Carmen L	Contreras		MSc	Universidad del Valle de Guatemala	Guatemala City, Guatemala	Field staff	
Rachel	Craik		BSc	University of Oxford	Oxford, UK	Reproductive health assistant	
Victor G	Davila-Roman		MD	Washington University in St. Louis	St. Louis, MO, US	Lead Investigator - Sonography	
Lisa	de las Fuentes		MD, MS	Washington University in St. Louis	St. Louis, MO, US	Investigator - Sonography	
Oscar	De León		PhD	Universidad del Valle de Guatemala; Emory University	Guatemala City, Guatemala; Atlanta, GA, US	Field staff; doctoral student	
Priya E	D'Souza		MPH	Emory University	Atlanta, GA, US	Central laboratory lead	
Lisa	Elon		MS, MPH	Emory University	Atlanta, GA, US	Statistician	
Juan G	Espinoza		BBA	Asociación Benéfica PRISMA	Lima, Peru	Field staff	

*First name, last name, and suffix (if applicable) are required and will appear in PubMed.

*First Name and Middle Initial(s)	*Last Name	*Suffix (eg, Jr, III)	Academic Degrees	Institution	Location (city, state/province, country)	Role or Contribution, eg, chair, principal investigator	Group (if more than 1 Group listed in the byline) and/or Subgroup (eg, Steering Committee)
Ahana	Ghosh		MS	Berkeley Air Monitoring Group	Berkeley, CA, US	Exposure core staff	
Dina	Goodman-Palmer		MPH, PhD	Johns Hopkins University	Baltimore, MD, US	Doctoral student	
Savannah	Gupton		MS	Emory University	Atlanta, GA, US	Central laboratory staff	
Sarah	Hamid		MPH, PhD	Emory University	Atlanta, GA, US	Doctoral student	
Steven A	Harvey		PhD	Johns Hopkins University	Baltimore, MD, US	Co-Lead Investigator - Behavioral Core	
Mayari	Hengstermann		PhD, MA	Universidad del Valle de Guatemala	Guatemala City, Guatemala	Pilot study investigator	
Ian	Hennessee		MPH, PhD	Emory University	Atlanta, GA, US	Doctoral student	
Phabiola M	Herrera		MD	Johns Hopkins University	Baltimore, MD, US	Research coordinator	
Marjorie	Howard		MSPH	Emory University	Atlanta, GA, US	Statistician	
Penelope P	Howards		PhD	Emory University	Atlanta, GA, US	Reproductive health associate	
Katherine	Kearns		PhD	University of Georgia	Athens, GA, US	Doctoral student	
Jacob	Kremer		PhD	University of Georgia	Athens, GA, US	Doctoral student	
Margaret A	Laws		MPH	Johns Hopkins University	Baltimore, MD, US	Peru Site study manager	
Grace E	Lee		MPH	Emory University	Atlanta, GA, US	Central laboratory staff	
Patricia M	Lenzen		M.Ed., RDCS	Washington University in St. Louis	St. Louis, MO, US	Sonography Core staff	
Jiawen	Liao		PhD	Emory University	Atlanta, GA, US	Doctoral Student	
Amy E	Lovvorn		MPH	Emory University	Atlanta, GA, US	Central study manager	
Julia N	McPeek		MPH	Emory University	Atlanta, GA, US	Central laboratory staff	
Rachel M	Meyers		M.A., RDCS	Washington University in St. Louis	St. Louis, MO, US	Sonography core staff	

Supplemental Online Content: Nonauthor Collaborators
*First name, last name, and suffix (if applicable) are required and will appear in PubMed.

***First Name and Middle Initial(s)**	***Last Name**	***Suffix (eg, Jr, III)**	Academic Degrees	Institution	Location (city, state/province, country)	Role or Contribution, eg, chair, principal investigator	Group (if more than 1 Group listed in the byline) and/or Subgroup (eg, Steering Committee)
J Jaime	Miranda		MD, PhD, MSc, FFPH	Universidad Peruana Cayetano Heredia	Lima, Peru	Pilot study investigator	
Erick	Mollinedo		MsC	Universidad del Valle de Guatemala; University of Georgia	Guatemala City, Guatemala; Athens, GA, US	Doctoral student	
Libny	Monroy		RN	Universiad del Valle de Guatemala	Guatemala City, Guatemala	Field staff	
Lawrence H	Moulton		PhD	Johns Hopkins University	Baltimore, MD, US	Investigator; lead statistician	
Durairaj	Natesan		PhD	Sri Ramachandra Institute of Higher Education and Research	Chennai, India	Site investigator	
Azhar	Nizam		MS	Emory University	Atlanta, GA, US	Statistician	
Jean de Dieu	Ntivuguruzwa		MS	Eagle Research Center	Kigali, Rwanda	Site data manager	
Parinya	Panuwet		PhD	Emory University	Atlanta, GA, US	Central laboratory staff	
Aris T	Papageorghiou		MD	University of Oxford	Oxford, UK	Lead Investigator - Reproductive Health	
Irma S	Piñeda Fuentes		MD MS	Universidad del Valle de Guatemala	Guatemala City, Guatemala	Project coordinator	
Ricardo	Piedrahita		PhD	Berkeley Air Monitoring Group	Berkeley, CA, US	Exposure core staff	
Naveen	Puttaswamy		PhD	Sri Ramachandra Institute of Higher Education and Research	Chennai, India	Investigator; India Site laboratory manager	
Elisa	Puzzolo		PhD	Global LPG Partnership; University of Liverpool	New York, NY, US; Liverpool, UK	Consultant, dissemination	
Karthikeyan D	Rajamani		PhD	Sri Ramachandra Institute of Higher Education and Research	Chennai, India	Site investigator	
Sarah	Rajkumar		PhD	Colorado State University	Fort Collins, CO, US	Post-doc	
Usha	Ramakrishnan		PhD	Emory University	Atlanta, GA, US	Investigator	

Supplemental Online Content: Nonauthor Collaborators
*First name, last name, and suffix (if applicable) are required and will appear in PubMed.

***First Name and Middle Initial(s)**	***Last Name**	***Suffix (eg, Jr, III)**	Academic Degrees	Institution	Location (city, state/province, country)	Role or Contribution, eg, chair, principal investigator	Group (if more than 1 Group listed in the byline) and/or Subgroup (eg, Steering Committee)
Rengaraj	Ramasami		MSc, MPhil	Sri Ramachandra Institute of Higher Education and Research	Chennai, India	Site investigator	
Alexander	Ramirez		BS	Universidad del Valle de Guatemala	Guatemala City, Guatemala	Guatemala Site data manager	
P. Barry	Ryan		PhD	Emory University	Atlanta, GA, US	Investigator, central laboratory	
Sudhakar	Saidam		MSc	Sri Ramachandra Institute of Higher Education and Research	Chennai, India	Site investigator	
Saritha	Sendhil		ME	Sri Ramachandra Institute of Higher Education and Research	Chennai, India	Site investigator	
Sheela S.	Sinharoy		PhD, MPH	Emory University	Atlanta, GA, US	Post-doc	
Kirk R.	Smith		PhD, MPH	University of California, Berkeley	Berkeley, CA, US	Investigator	
Damien	Swearing		MSc	University of Georgia	Athens, GA, US	Doctoral student	
Ashley K.	Toenjes		RDCS	Washington University in St. Louis	St. Louis, MO, US	Sonography core staff	
Viviane	Valdes		PhD, MPH	Emory University	Atlanta, GA, US	Doctoral student	
Amit	Verma		MSPH	Emory University	Atlanta, GA, US	Statistician	
Megan	Warnock		MSPH	Emory University	Atlanta, GA, US	Statistician	
Wenlu	Ye		MDP, PhD	Emory University	Atlanta, GA, US; Berkeley, CA, US	Doctoral student	
Bonnie N.	Young		PhD, MPH	Colorado State University	Collins, CO, US	Post-doc	
Ashley	Younger		PhD	University of California - San Francisco	San Francisco, CA, US	Doctoral student	
