# Supplementary material for: Exposure to Household Air Pollution From Biomass Cooking and Severe Pneumonia in Infants
Source: JAMA Netw Open. 2025 Oct 29;8(10):e2538721. doi: 10.1001/jamanetworkopen.2025.38721 (PMC12573034; doi:10.1001/jamanetworkopen.2025.38721)
Supplement: Supplement 3. — Data Sharing Statement [file jamanetwopen-e2538721-s003.pdf]

Data Sharing Statement

McCracken. Exposure to Household Air Pollution From Biomass Cooking and Severe Pneumonia in Infants. *JAMA Netw Open*. Published October 29, 2025.
doi:10.1001/jamanetworkopen.2025.38721

Data
Data available: Yes
Data types: Deidentified participant data
How to access data: Email wcheckl1@jhmi.edu for access
When available: With publication

Supporting Documents
Document types: Statistical/analytic code
How to access documents: Statistical code available with publication
When available: With publication

Additional Information
Who can access the data: Statistical code available on Online Suplemment
Types of analyses: All
Mechanisms of data availability: After approval of proposal
